# START: A Versatile Platform for Bacterial Ligand Sensing with Programmable Performances

**DOI:** 10.1002/advs.202402029

**Published:** 2024-07-29

**Authors:** Jeongwon Kim, Minchae Seo, Yelin Lim, Jongmin Kim

**Affiliations:** ^1^ Department of Life Sciences Pohang University of Science and Technology Pohang 37673 South Korea

**Keywords:** aptamers, biosensors, genetic circuit construction, riboswitches, synthetic riboregulators

## Abstract

Recognition of signaling molecules for coordinated regulation of target genes is a fundamental process for biological systems. Cells often rely on transcription factors to accomplish these intricate tasks, yet the subtle conformational changes of protein structures, coupled with the complexity of intertwined protein interaction networks, pose challenges for repurposing these for bioengineering applications. This study introduces a novel platform for ligand‐responsive gene regulation, termed START (Synthetic Trans‐Acting Riboswitch with Triggering RNA). Inspired by the bacterial ligand sensing system, riboswitch, and the synthetic gene regulator, toehold switch, the START platform enables the implementation of synthetic biosensors for various ligands. Rational sequence design with targeted domain optimization yields high‐performance STARTs with a dynamic range up to 67.29‐fold and a tunable ligand sensitivity, providing a simple and intuitive strategy for sensor engineering. The START platform also exhibits modularity and composability to allow flexible genetic circuit construction, enabling seamless implementation of OR, AND, and NOT Boolean logic gates for multiple ligand inputs. The START design principle is capable of broadening the suite of synthetic biosensors for diverse chemical and protein ligands, providing a novel riboregulator chassis for synthetic biology and bioengineering applications.

## Graphical abstract

This study presents a novel platform for bacterial ligand sensing, termed START. The ligand‐responsive aptamer elements are integrated with synthetic RNA regulatory devices to create flexible circuit architectures with diverse ligand inputs, tunable sensitivity, and dynamic ranges. The plug‐and‐play features with logic processing capability further underscores the broad application of START platform for engineering synthetic biological sensors.

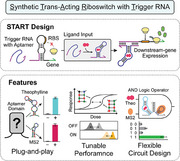

## 1 Introduction

Biological systems, in their essence, represent a complex network of meticulously coordinated interactions that span molecular, cellular, and organismal levels.^[^ ^1^ ^]^ Central to the performance of these dynamical biological regulatory networks is the ability to sense the whole range of environmental and intracellular changes and to coordinate gene expression programs to achieve the optimal outputs.^[^ ^2^ ^]^ Protein regulatory factors, such as transcription factors, typically serve as molecular sentinels that modulate gene expression in response to internal and external cues.^[^ ^3^ ^]^ These protein‐based mechanisms are foundational to our understanding of biological regulation, and therefore, largely harnessed for bioengineering and synthetic biology.^[^ ^4^ ^]^ Nonetheless, the complexity of protein structures and the intricate networks of protein interactions continue to pose challenges for implementing protein‐based regulators as designable synthetic biological building blocks.^[^ ^5^ ^]^

In this respect, RNA‐based regulatory mechanisms have emerged as a promising frontier to expand the horizon of engineered biological systems with desired specifications. The structural flexibility and sequence specificity of RNA molecules allow fine‐tuning of protein expression in a variety of biological systems,^[^ ^6^ ^]^ performing key regulatory roles in fundamental processes such as metabolism,^[^ ^7^ ^]^ development,^[^ ^8^ ^]^ and immune response.^[^ ^9^ ^]^ To capitalize on the versatility of RNA, researchers have extensively investigated natural riboregulators^[^ ^10^ ^]^ and their synthetic alternatives,^[^ ^11^ ^]^ striving to harness their potential across diverse disciplines including metabolic engineering,^[^ ^12^ ^]^ synthetic gene circuit construction,^[^ ^13^ ^]^ and biosensor development.^[^ ^14^ ^]^ In recent years, significant advances in computational tools led to a more accurate and systematic approach for riboregulator research.^[^ ^15^ ^]^ By employing sophisticated prediction algorithms for the secondary structure and thermodynamic properties of RNA sequences, RNA devices came forth as programmable synthetic biological toolkits.^[^ ^16^ ^]^

**Figure 1:**
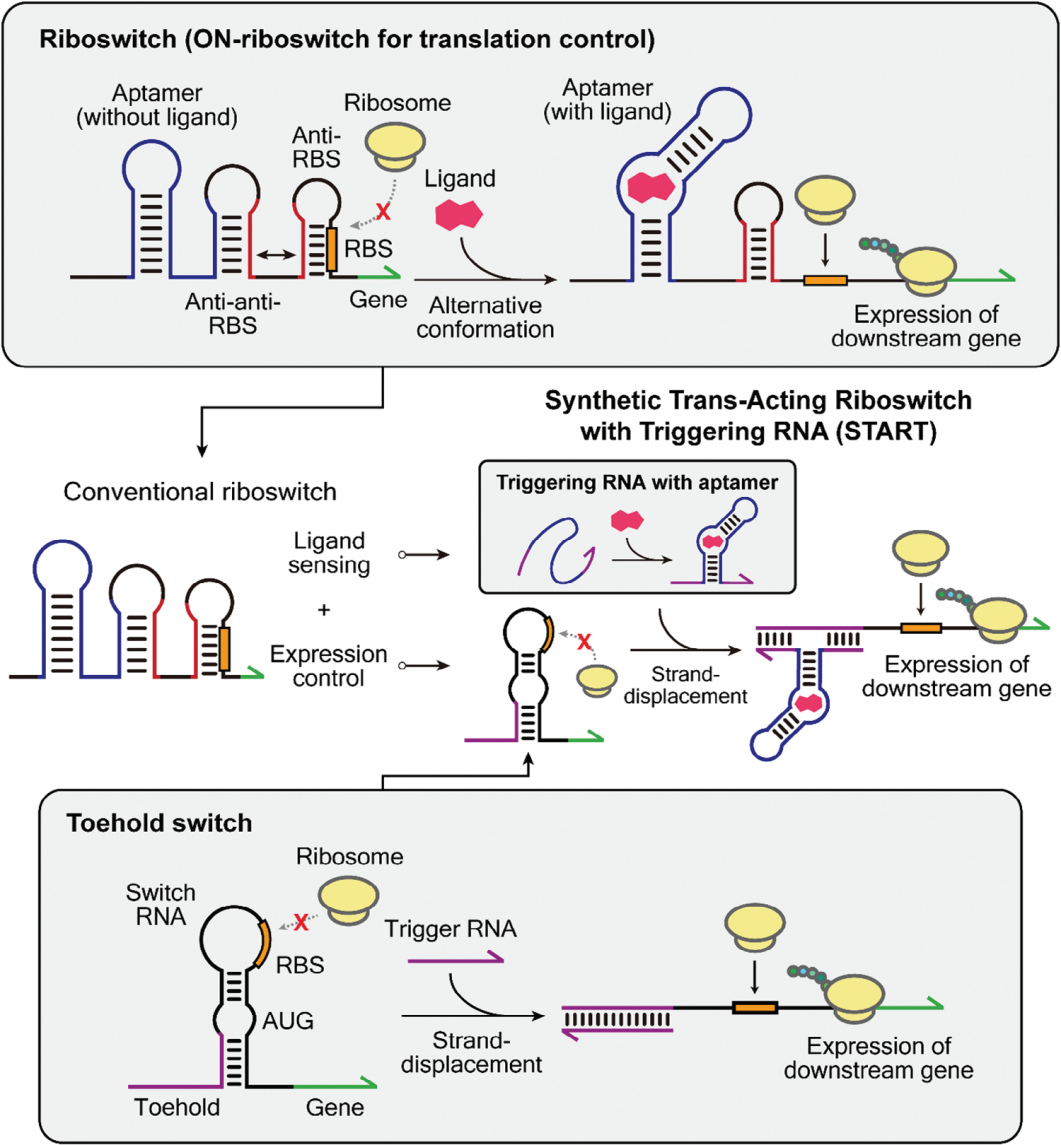
Schematic illustration of START design in relation to conventional riboswitch and toehold switch. (Top panel) Mechanism of translational ON‐riboswitch. The riboswitch adopts a specific secondary structure due to the interaction between the aptamer domain (blue) and the anti‐sequence domain (red), which in turn controls the downstream gene expression. Ligand binding within the aptamer domain induces a cascade of alternative folding to allow gene expression. (Bottom panel) Mechanism of the toehold switch.^[^ ^28^ ^]^ Switch RNA is designed to repress downstream gene expression by sequestering the RBS and start codon. Complementary trigger RNA initiates RNA‐RNA interactions via the toehold domain (purple), inducing strand‐displacement and the active translation of the downstream gene. (Middle panel) Schematic of START design. The riboswitch function is engrafted to the toehold switch architecture by incorporating aptamer domain within the triggering RNA sequence, which in turn controls the expression of switch RNA. By separating the ligand sensing aptamer sequence from the expression control module, this trans‐acting architecture may provide design flexibility to each functional domain of riboswitches.

The riboswitch is one of the notable riboregulators that reflects the advantages of RNA molecules in regulatory mechanisms. Commonly found at the 5′ untranslated region (5′ UTR) of bacterial mRNA,^[^ ^10b^ ^]^ riboswitches regulate downstream gene expression by conformational changes upon binding to specific target ligands (**Figure** **1**, top panel). Riboswitches mainly consist of two functional modules: a ligand sensing module where the target ligand is recognized by the aptamer sequence, and an expression control module that participates in conformational switching of the key regulatory elements.^[^ ^17^ ^]^ These two modules are interconnected with complementary sequences termed anti‐sequences for regulatory targets such as ribosome binding site (RBS)^[^ ^18^ ^]^ and rho‐independent terminator.^[^ ^19^ ^]^ Ligand binding stabilizes the conformation of aptamer sequence domain to direct alternative base‐pairing interactions of the anti‐sequences, which in turn switches the configuration of the expression control module of the mRNA.^[^ ^20^ ^]^ Since the discovery of thiamine pyrophosphate riboswitch 20 years ago,^[^ ^21^ ^]^ natural riboswitches that respond to different types of ligands such as enzyme cofactors,^[^ ^22^ ^]^ nucleotide precursors,^[^ ^10^, ^23^ ^]^ or metal ions^[^ ^24^ ^]^ have been extensively characterized, and demonstrated their potential in diverse applications including biosensor development,^[^ ^25^ ^]^ metabolic flux engineering,^[^ ^26^ ^]^ and therapeutics.^[^ ^27^ ^]^

Inspired by natural and engineered riboswitches, we developed a synthetic riboregulator platform, named START (Synthetic Trans‐Acting Riboswitch with Triggering RNA). This aims to provide a versatile engineering platform that senses target ligands and regulates downstream gene expression, by addressing the sequence dependency of conventional riboswitches originating from the use of anti‐sequences and aptamers in the same RNA transcript by separating the aptamer sequence from the expression control module (Figure 1, middle panel). Still, the conformation change induced by ligand binding can be similarly achieved by a separate input RNA with an inserted aptamer sequence. We incorporated this design principle into de novo designed synthetic riboregulators, toehold switches^[^ ^28^ ^]^ (Figure 1, bottom panel). The incorporation of riboswitch function into toehold switches allowed us to leverage the remarkable sequence flexibility and orthogonality of toehold switches and to implement complex genetic circuits using a library of toehold switches and ribocomputing devices.^[^ ^29^ ^]^ We demonstrated the applicability of our design principle by employing theophylline, tetracycline, and MS2 coat protein aptamers to obtain high‐performance STARTs with a maximum fold change of 67.29‐fold in *Escherichia coli* (*E. coli*). Characterization of these novel riboregulators demonstrated controllable expression levels, dynamic ranges, and operational ranges by adjusting key design elements while maintaining robust functionality for a number of sequence contexts. START designs also can be integrated to perform complex logical regulation of gene expression with multiple input ligands, indicating that our design approach could be a starting point for genetic circuit constructions and enrich the application area of synthetic riboregulators.

## 2 Results

### 2.1 Design Strategy for START Platform

Inspired by natural riboswitches, a wide variety of aptamer‐based synthetic regulators were utilized to reprogram cellular signaling from ligand input to gene expression.^[^ ^30^ ^]^ These synthetic riboswitches often leverage aptamer sequences to control gene expression in a cis‐manner at the 5′ UTR of the mRNA.^[^ ^10b^ ^]^ These cis‐regulatory designs that involve a direct interaction between the ligand‐sensing aptamer and the expression control module typically require careful adjustment of sequence elements and screening for optimization.^[^ ^31^ ^]^ An alternative approach utilized synthetic regulators containing aptamer domains acting in trans, targeting specific elements in a ligand responsive manner.^[^ ^32^ ^]^ For instance, Liu et al. demonstrated synthetic regulators that combine an antisense RNA and an aptamer domain, to control gene expression in a multiplexed fashion.^[^ ^33^ ^]^

**Figure 2:**
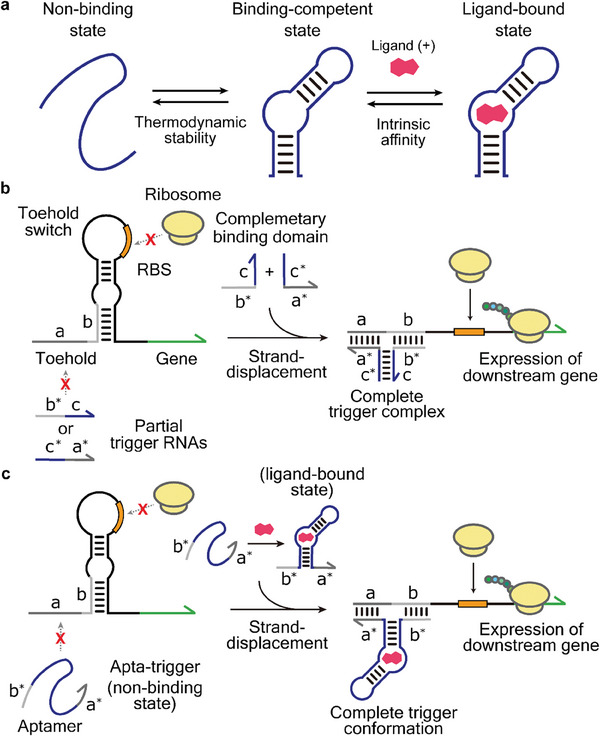
Design of START platform. a) Schematic illustration of population shift mechanism for an aptamer. The presence of ligand can shift the equilibrium state of aptamer towards binding‐competent conformation. b) Toehold switch‐based AND logic circuit. The trigger RNA sequence is divided into two separate strands with a complementary domain for self‐assembly, allowing the switch activation only when both trigger RNAs are present to form a complete trigger complex. c) Proposed mechanism of START. The aptamer sequence (blue) is inserted between the two partial trigger domains (a* and b*) to form an Apta‐trigger. In the absence of ligand, the aptamer domain is presumably unstructured such that the two partial trigger domains are not in proximity to allow for switch activation. With the ligand present, the ligand‐aptamer complex can stabilize the conformation of Apta‐trigger, which in turn activates switch RNA for downstream gene expression. The asterisks (*) denote domains with complementary sequences.

To take advantage of design flexibility afforded by trans‐acting designs, while at the same time to tap into the fast‐growing repertoire of synthetic RNA regulatory devices, we aimed to integrate the ligand responsive aptamer elements into synthetic RNA regulators. To design a ligand‐responsive RNA device, we first focused on the population shift mechanism of aptamer sequence, often employed for the rational design of molecular switches.^[^ ^34^ ^]^ In this model, a structure‐switching molecule, such as an aptamer, interconverts between two states: a non‐binding state that does not readily interact with ligand, and a binding‐competent state with a high affinity for ligand (**Figure** **2**a). In the absence of the ligand, the aptamer is in equilibrium between these two states as dictated by the thermodynamic stability of each conformation. In the presence of ligand, ligand binding to the aptamer shifts the equilibrium toward the ligand‐aptamer complex with aptamer taking its binding‐competent conformation.^[^ ^35^ ^]^ Preferential ligand binding to certain aptamer states can lead to an equilibrium shift, inducing further conformational changes downstream as commonly found in conventional riboswitches.

Here, we aimed to embed this structural switching capability of aptamer into synthetic input RNA molecules that in turn control the translation states of synthetic riboregulators, toehold switches.^[^ ^28^ ^]^ In the toehold switch library, a switch RNA contains a hairpin structure that blocks translation by sequestering the RBS and start codon, which in turn can be resolved via the strand displacement by an input RNA molecule to allow translation of downstream gene (Figure 1, bottom panel). Utilizing the fact that a cognate input RNA contains both the toehold‐binding domain a* that targets the single‐stranded region of switch RNA for initial binding and the strand‐displacement domain b* that disrupts the otherwise stable hairpin structure, an AND logic was previously demonstrated by dividing the toehold‐binding domain and strand‐displacement domain into separate molecules with respective complementary domains c‐c* for complex formation (Figure 2b).^[^ ^29^ ^]^ Thus, each partial trigger RNA (b*c or c*a*) cannot activate the switch RNA since neither sequence alone is able to unwind the hairpin structure effectively, while a complex formed by both partial triggers presents the two domains a* and b* in proximity such that strand displacement occurs much like a regular input RNA given that the interaction between the complementary domains c and c* is sufficiently stable.

Taking inspiration from the AND gate circuit, we devised an aptamer‐containing trigger RNA (referred to as Apta‐trigger), where the formation of a stable trigger RNA conformation is regulated by the aptamer‐ligand interactions that stabilize the otherwise unstructured aptamer domain (Figure 2c). We reasoned that the aptamer sequence can be engineered to predominantly adopt a non‐binding state in the absence of ligand such that the two partial trigger domains a* and b* surrounding the aptamer would not be in close proximity. This would result in ineffective strand displacement of cognate switch RNA much like having a weak c‐c* domain for complex formation in the AND gate. On the other hand, with the target ligand present, the ligand‐aptamer complex adopts a stable conformation where the two partial trigger domains are in close proximity to allow active translation from the switch RNA. Note that in this proposed mechanism, unlike conventional riboswitches, the aptamer sequence is engrafted in a separate input RNA strand, the trigger RNA, such that a direct interaction of the aptamer with the expression control module, the switch RNA, can be minimized. Therefore, this approach could minimize the intricate interdependency of several sequence domains typical for cis‐regulatory riboswitches, providing ways to devise novel platform for ligand sensing and gene regulation with simple design rules.

### 2.2 Implementation of START with Theophylline Aptamer

**Figure 3:**
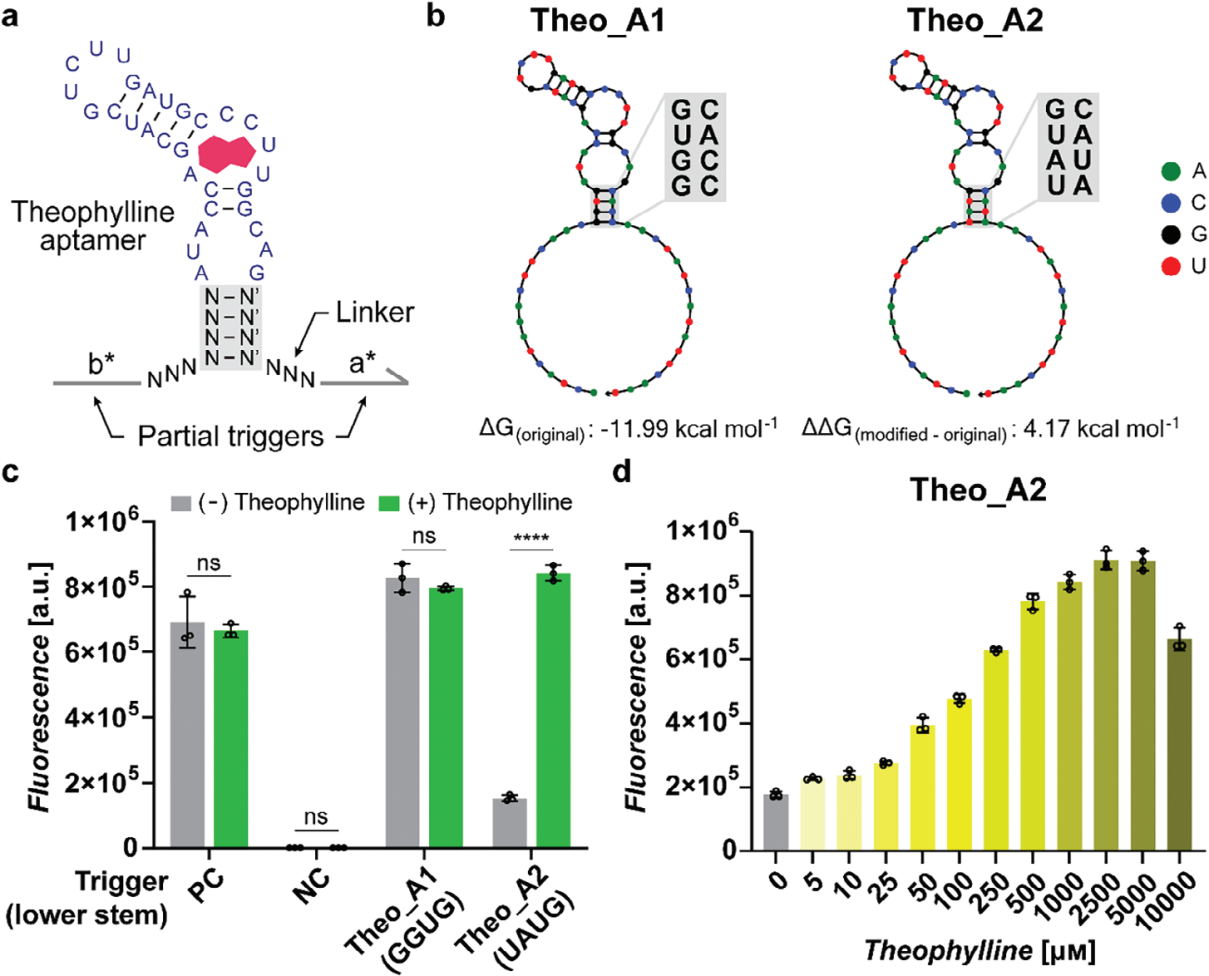
In vivo characterization of theophylline Apta‐triggers for START. a) Detailed schematic of theophylline Apta‐trigger design. The theophylline aptamer is inserted between the two partial trigger domains (a* and b*) with 3‐base linker sequences on both sides. The lower stem region of the theophylline aptamer subject to engineering is marked by a gray box. b) Predicted secondary structure and free‐energy of theophylline Apta‐trigger Theo_A1 (left) and Theo_A2 (right) with lower stem sequence modification. c) GFP fluorescence of toehold switch A and trigger RNA pairs in the absence (gray bars) and presence (green bars) of 5 mм theophylline. Positive control (PC) used the toehold switch trigger A without the aptamer sequence, while negative control (NC) used the decoy RNA without any predicted interactions. d) GFP fluorescence output for switch A and Theo_A2 pair for different concentrations of theophylline inputs. P‐values were determined by an unpaired *t*‐test, with P > 0.05 designated by “ns” and P ≤ 0.0001 designated by “****”. Error bars are the standard deviation (SD) from three biological replicates.

To test the feasibility of START design principles, we chose the theophylline aptamer^[^ ^36^ ^]^ as an ideal candidate due to the well‐established mechanistic understanding of the aptamer and its wide adoption in synthetic riboregulators (**Figure** **3**a). We constructed a theophylline Apta‐trigger by incorporating the optimized theophylline aptamer sequence^[^ ^37^ ^]^ with the partial trigger sequences for the toehold switch with a GFP output (Theo_A1 in Figure 3b). For different Apta‐trigger variants, we chose a naming convention as follows: the target ligand of aptamer followed by the cognate toehold switch for the partial triggers and the version of sequence variants (e.g., Theo_A1 trigger contains theophylline aptamer and partial triggers for toehold switch A with the first sequence variant). In vivo characterization showed high fluorescence signals regardless of the ligand treatment for Theo_A1 trigger, possibly due to the aptamer sequence within Theo_A1 mainly adopting a binding‐competent state even in the absence of the ligand (Figure 3c). To improve the dynamic range, we sought to reduce the thermodynamic stability of the binding‐competent state for Theo_A1 trigger through modulation of the aptamer sequence. In order to minimize the impact on the aptamer's affinity to its target ligand,^[^ ^38^ ^]^ the lower stem sequences of the aptamer, which presumably serve a structure‐stabilizing function, were targeted for sequence modifications (indicated as a gray box in Figure 3a). A modified theophylline Apta‐trigger with a relatively weak lower stem sequence (Theo_A2 in Figure 3b) exhibited a low GFP fluorescence in the absence of the theophylline, but showed a high fluorescence level with the theophylline input (Figure 3c). Alternative control trigger RNA designs with the same length of linker sequence as aptamer domain but without ligand binding activity showed no significant change in fluorescence output upon theophylline treatment (Figure S1, Supporting Information). The GFP output expression levels of toehold switch A with Theo_A2 construct were continuously adjusted as the theophylline input levels were increased, indicating that the Theo_A2 trigger conformations were responsive to theophylline input concentrations as desired (Figure 3d). However, when theophylline is treated at a high concentration (10 mм), a slight reversal in fluorescence activation was observed, likely due to growth changes upon high theophylline ligand treatment (Figure S2, Supporting Information).^[^ ^39^ ^]^ On the other hand, the Theo_A1 trigger and toehold switch A pairs showed little change in fluorescence output for a wide range of theophylline concentrations (Figure S3, Supporting Information). These results indicate that the START design scheme that combines the toehold switches and Apta‐triggers operates in *E. coli*, and that the rational modification of the aptamer domains that serve a structure stabilizing function could be an effective strategy for enhancing the dynamic range.

### 2.3 Systematic Analysis and Sequence Optimization of START

**Figure 4:**
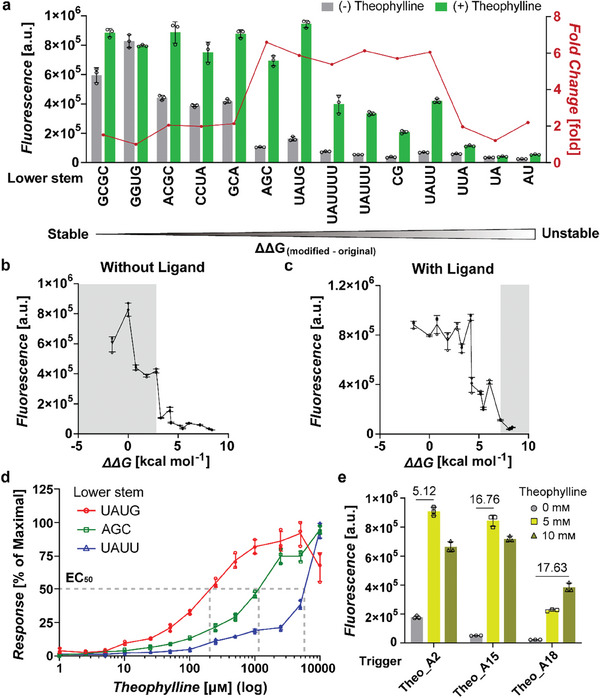
Thermodynamic analysis and optimization of theophylline Apta‐triggers. a) GFP fluorescence output of toehold switch A with theophylline Apta‐trigger variants, in the absence (gray bars) and presence (green bars) of 5 mм theophylline. The fold changes of GFP fluorescence outputs in the absence and presence of theophylline for different Apta‐trigger variants are plotted in red. The lower stem sequence of the theophylline aptamer on the 5′ side are indicated below (e.g., GGUG for Theo_A1 and UAUG for Theo_A2; The complete sequences for Theo_A1 to Theo_A14 are listed in Table S3, Supporting Information). b, c) GFP fluorescence without theophylline (b) and with 5 mм theophylline (c) for Apta‐trigger variants ordered by the free‐energy difference with respect to the original theophylline aptamer (ΔΔG). Design variants with high background fluorescence or low signal activation are marked in gray. d) Dose‐response curves of theophylline Apta‐trigger variants with large shift in the activation thresholds. Response curves without normalization are shown in Figure S4 (Supporting Information). e) Characterization of optimized theophylline Apta‐triggers. The maximum fold changes are indicated above the bar graph for each design variant. Error bars represent the SD from three biological replicates.

Systematic adjustment of the structural stability of aptamer domains could help elucidate the design principles for Apta‐triggers. Thus, we constructed a number of sequence variants that target the lower stem domain of theophylline aptamer embedded within theophylline Apta‐triggers. The calculated thermodynamic energies for the lower stem domain using NUPACK analysis tool^[^ ^40^ ^]^ (Note S1, Supporting Information) spanned from more stable than the original aptamer (ΔΔG < 0 kcal mol^−1^) to unstable (ΔΔG > 0 kcal mol^−1^) (**Figure** **4**a). Based on the results on Theo_A1 and Theo_A2 constructs, we reasoned that slightly weaker lower stem domain variants compared to the unmodified theophylline aptamer may provide additional functional Apta‐triggers. In vivo characterization revealed that several Apta‐trigger designs exhibited switch‐like behavior in response to ligand treatment with up to 7‐fold changes. In particular, the sequence variants with intermediate stability (3 kcal mol^−1^ < ΔΔG < 7 kcal mol^−1^) tended to show the largest dynamic range among the sequence variants. A general trend was observed where the constructs with weaker lower stem sequence showed generally lower background signals (Figure 4b), whereas those with strong lower stem sequence tended to have higher fluorescence intensity in the presence of the target ligand (Figure 4c). These results support the proposed mechanism of conformational change of aptamer domains upon ligand binding, which in turn affect the proximity of split trigger domains, ultimately controlling the activation of target switch RNAs. Balancing the stability of the aptamer lower stem is one of the important considerations to optimize the dynamic range of START designs, where the strong and weak constructs with limited structure‐switching capability upon ligand input could negatively impact the performance (gray regions in Figure 4b,c). The rationale for START designs with thermodynamic analysis and empirical optimization steps are further described in Supporting Information (Note S2, Supporting Information).

Interestingly, adjusting the aptamer lower stem domain also shifted the ligand sensitivity of Apta‐triggers where the half maximal effective concentrations (EC50) for the Apta‐trigger with a relatively weak UAUU stem was 30‐fold higher compared to the trigger with a stronger UAUG stem (Figure 4d; Figure S4, Supporting Information). This indicates that the START platform can be amenable to rational sequence modification for desired ligand sensing performance, both in terms of the dynamic range and the operational range.

Next, the effect of the insertion position of aptamer domain and the surrounding partial trigger lengths of Apta‐triggers were investigated (Figure S5, Supporting Information). The designs with the aptamer insertion at the midpoint was apparently near optimal and no further improvement was observed for different insertion positions. On the other hand, trimming the partial trigger domains reduced the leaky expression due to spontaneous activation of switch RNA in the absence of ligand. Applying these new findings resulted in improved dynamic range of the theophylline Apta‐triggers (Figure 4e; Figures S6 and S7, Supporting Information). Trimming both partial trigger domains yielded a theophylline Apta‐trigger with a high ON‐state expression and a dynamic range of 16.76‐fold (Theo_A15 in Figure 4e), and an additional substitution of the lower stem with a weak base pair improved the fold‐change up to 17.63‐fold (Theo_A18 in Figure 4e). Additionally, we compared the performance of theophylline START constructs with a previously reported cis‐acting theophylline riboswitch.^[^ ^41^ ^]^ Under the same experimental conditions, the cis‐acting riboswitch exhibited a slightly better sensitivity, whereas the optimized START constructs showed higher fluorescence outputs and increased fold‐changes (Figure S8, Supporting Information).

### 2.4 Characterization of Design Space for START Platform

To demonstrate the engineering versatility and broad applicability of the START platform, we conducted several investigations to address various aspects of its functionality. First, to test the robustness of START designs for a diverse set of sequence context, the performance was evaluated for the toehold switch and the corresponding Apta‐trigger variants. The toehold switch variants with different promoters^[^ ^42^ ^]^ and RBS library^[^ ^43^ ^]^ maintained desired response to cognate theophylline Apta‐triggers (Figures S9 and S10, Supporting Information). Additionally, introducing different toehold switches and their cognate partial trigger sequences to realize a different set of START designs still retained functional response to theophylline ligand (Figure S11, Supporting Information), suggesting the design flexibility and modularity of the START platform.

Next, we investigated the impact of the molecular ratio of the trans‐acting Apta‐trigger and the toehold switch mRNA on the system performance. By comparing the response of theophylline STARTs for trigger RNAs on three different plasmid backbones, we found that higher expression levels of Apta‐trigger RNA correlated with increased basal and activated expression levels as well as reduced activation threshold for ligand inputs (Figure S12, Supporting Information). This indicates that the trans‐acting RNA expression levels provide an additional tuning knob for START performance. To further assess the applicability of START platform, a theophylline START was introduced into a probiotic *E. coli* Nissle 1917 strain. This experiment showed a 30‐fold increase in fluorescence output upon theophylline treatment, similar to the results observed in other *E. coli* strains (Figure S13, Supporting Information).

**Figure 5:**
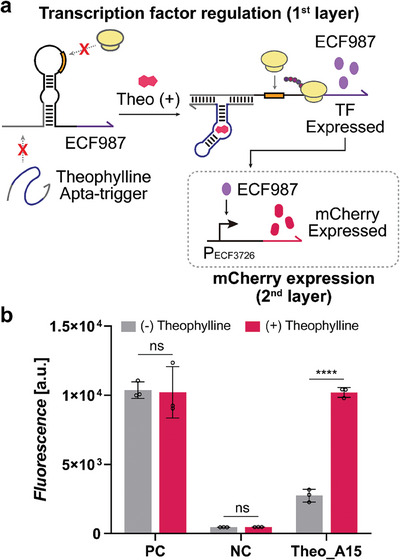
Two‐layered circuit construction with START. a) Schematic of two‐layered regulatory circuit with transcription factor and START. Synthetic transcription factor, ECF987, is regulated by theophylline START, which in turn drives the expression of mCherry reporter gene in the second layer with its cognate synthetic promoter, P_ECF3726_. b) mCherry fluorescence output of the two‐layered regulatory circuit in the absence (gray bars) and presence (red bars) of 5 mм theophylline. Positive control (PC) used the toehold switch trigger A without the aptamer sequence, and negative control (NC) used the decoy RNA without any predicted interations. P‐values were determined by an unpaired *t*‐test, with P > 0.05 designated by “ns” and p ≤ 0.0001 designated by “****”. Error bars represent the SD from three biological replicates.

We then explored the use of STARTs for synthetic gene circuit architecture by designing and implementing a multi‐layered circuit incorporating transcription factors (**Figure** **5**a). In this setup, the expression of transcription factor ECF987^[^ ^44^ ^]^ is under the control of theophylline START in the first layer, whereas the mCherry reporter expression is regulated by a cognate promoter for ECF987 in the second layer. Upon theophylline treatment, the layered circuit showed proper activation of mCherry reporter expression, demonstrating that START designs could be utilized as design elements for synthetic gene circuits (Figure 5b).

**Figure 6:**
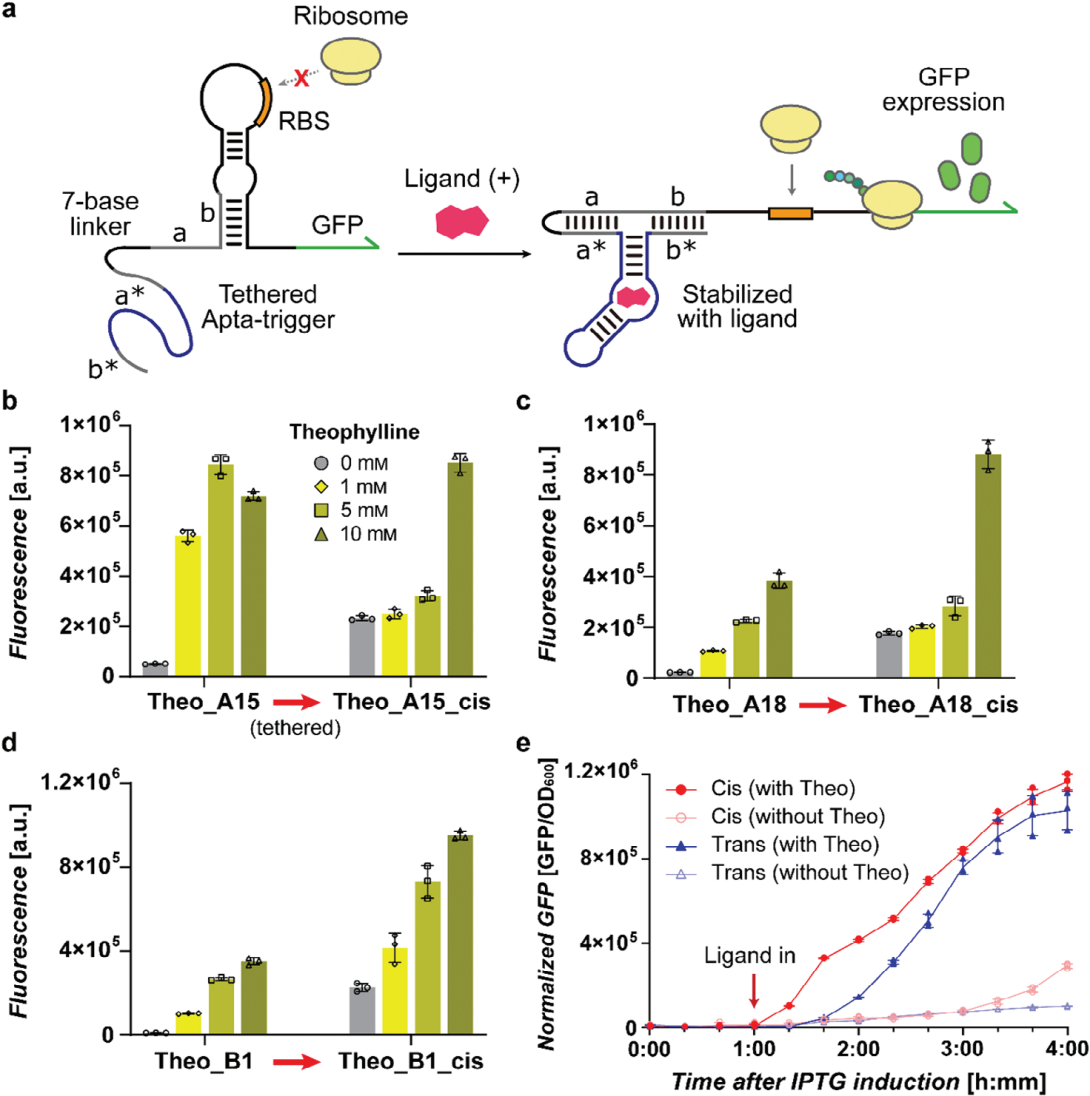
Design and characterization of cis‐acting START platform. a) Schematic of cis‐acting START device. A linear linker sequence of 7‐bases connects the Apta‐trigger to its cognate switch RNA, enabling strand displacement to occur in cis upon ligand binding. b‐d) GFP fluorescence outputs of trans‐ and cis‐acting START designs. e) Time‐course measurements of fluorescence outputs for trans‐ and cis‐acting START designs (Theo_A18). Theophylline was treated at 10 mм one hour after induction by IPTG that allow expression of START components, and normalized fluorescence outputs were monitored. Error bars represent the SD from three biological replicates.

Finally, to test the impact of colocalizing sensor domains with switches much like many natural riboswitch designs, a cis‐regulatory form of START was constructed by tethering the Apta‐trigger to its cognate toehold switch (**Figure** **6**a). Notably, all of the cis‐regulatory form of STARTs showed response to their respective ligands (Figure 6b–d). In these cis‐regulatory designs, the background signals in the absence of ligands were increased possibly due to the increased local concentration of sensor domains that negatively impacted the overall dynamic range. In terms of response time, the cis‐acting designs showed faster response of output fluorescence upon ligand treatment when compared to the trans‐acting designs (Figure 6e). These results indicate that both designs could be applicable for synthetic circuit designs with the cis‐acting designs having the advantage of faster response time and the trans‐acting designs having the advantage of tighter leakage control.

### 2.5 Expansion of the Sensing Repertoire through Aptamer Sequence Substitution

**Figure 7:**
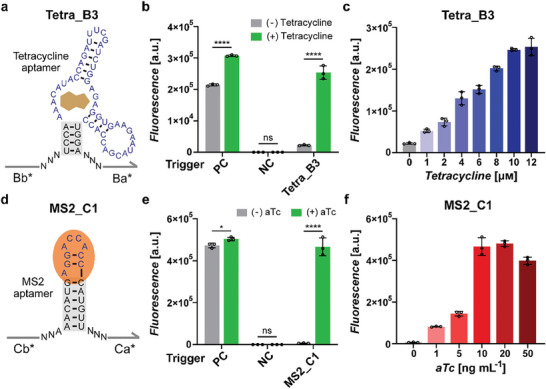
Design and characterization of STARTs for tetracycline and MS2 coat protein. a) Detailed schematics of the tetracycline Apta‐trigger, Tetra_B3. The lower stem region of tetracycline aptamer subject to engineering is marked by a gray box. b) GFP fluorescence of toehold switch B and trigger RNA pairs in the absence (gray bars) and presence (green bars) of 12 µм tetracycline. Positive control (PC) used the toehold switch trigger B without the aptamer sequence, while negative control (NC) used the decoy RNA without any predicted interactions. c) GFP fluorescence output for switch B and Tetra_B3 pair for different concentrations of tetracycline inputs. d) Detailed schematics of the MS2 coat protein Apta‐trigger, MS2_C1. The lower stem region of MS2 aptamer subject to engineering is marked by a gray box. e) GFP fluorescence of toehold switch C and trigger RNA pairs without (gray bars) and with (green bars) MS2 induction, by 10 ng mL^−1^ anhydrotetracycline (aTc) treatment. PC used the toehold switch trigger C, and NC used the decoy RNA. f) GFP fluorescence output for switch C and MS2_C1 pair for different induction levels for MS2 expression. P‐values were determined by an unpaired *t*‐test, with P > 0.05 designated by “ns”, P ≤ 0.05 designated by “*”, and P ≤ 0.0001 designated by “****”. Error bars are the SD from three biological replicates.

Since the START design can function for arbitrary aptamer sequences in principle, we constructed additional Apta‐triggers using alternative aptamer sequences building on the same design principles. We examined the tetracycline and its aptamer sequence,^[^ ^45^ ^]^ for incorporation into the START designs. First, the potential sequence interaction of the tetracycline aptamer and trigger domains were found to be negligible. Next, the strong lower stem region of known tetracycline aptamer was weakened by adjusting the domain lengths and sequences analogous to the theophylline Apta‐trigger design (**Figure** **7**a; Figure S14a, Supporting Information). Although the fluorescence measurements tended to show a slight increase following treatment with tetracycline (Figure S15, Supporting Information), the modified tetracycline Apta‐triggers exhibited a significant difference in downstream gene expression depending on the presence of the target ligand (Figure 7b,c), with different dynamic and operating ranges dependent on the stability of lower stem region following the general trend observed for the theophylline cases (Figure S14b,c, Supporting Information).

Encouraged by this finding, we further investigated the feasibility of START for the aptamers specific to protein ligands in addition to small molecules. We chose the MS2 coat protein aptamer,^[^ ^46^ ^]^ widely used for live‐cell imaging of mRNA (Figure S16, Supporting Information).^[^ ^47^ ^]^ MS2 Apta‐trigger constructed with the reported aptamer sequence, MS2_C1, exhibited a large dynamic range of 67.29‐fold (Figure 7d–f). The lower stem trimming approach to further weaken the aptamer structure resulted in a number of MS2 Apta‐triggers with considerable fold‐activation (Figure S17, Supporting Information). For those functional Apta‐trigger constructs with different aptamer domains, we found that an aptamer lower stem with 3 to 5 bases mainly consisting of weak A‐U base pairs was suitable for functionality, consistent with previous studies on structure switching aptamers.^[^ ^32^, ^35^ ^]^

For the MS2 Apta‐triggers, interestingly, constructs with trimmed lower stem sequence exhibited increased leakage in the absence of MS2 coat protein expression. It is unclear why the leakage level was increased upon aptamer lower stem trimming although there could be specific sequence features and unpredicted structure for the unmodified aptamer that limited stochastic switch activation. Nevertheless, the START for MS2 protein was demonstrated to function robustly in a variety of sequence contexts (Figure S18, Supporting Information). These results suggest that the START platform can offer a simple and convenient strategy for expanding the repertoire of biosensors for both small chemicals and protein ligands, by simply adopting the target aptamer sequence into the toehold switch library.

### 2.6 Evaluation of START Components Orthogonality

**Figure 8:**
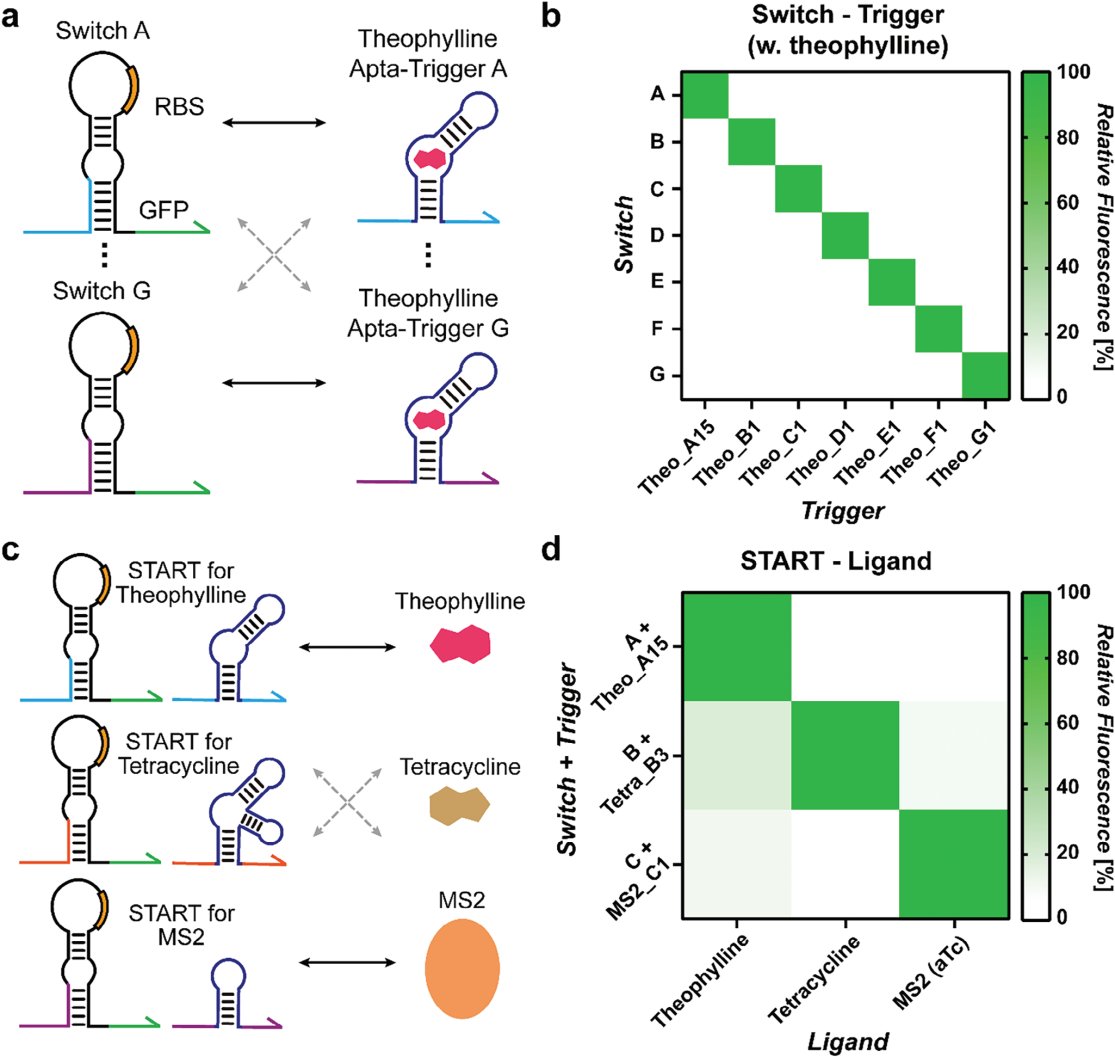
Orthogonality assessment between STARTs. a) Experimental scheme to test the orthogonality of switch and theophylline Apta‐trigger pairs. b) Crosstalk measured by flow cytometry for all switch‐trigger combinations. Relative fluorescence was determined by taking the GFP output measured for a given trigger‐switch combination and dividing it by the GFP output measured for the switch with its cognate Apta‐trigger (diagonal). Theophylline was treated at a concentration of 10 mм. c) Experimental scheme to test orthogonality for different ligand inputs. d) Crosstalk measured by flow cytometry for all switch‐trigger pairs and input ligands. Relative fluorescence was determined by taking the GFP output for a given Apta‐trigger‐switch pair and ligand combination and dividing it by the GFP output measured for the Apta‐trigger‐switch pair with its cognate input ligand (diagonal). Theophylline was treated at 10 mм, tetracycline was treated at 12 µм, and aTc was treated at 10 ng mL^−1^. Relative fluorescence value for each condition represents the mean of three biological replicates.

The orthogonality of regulatory components is a prerequisite to implement complex regulatory circuits, and therefore, we evaluated the orthogonality of secured START components. First, a number of theophylline Apta‐triggers that contain different trigger domain sequences were chosen together with their corresponding switch RNAs (**Figure** **8**a). The fluorescence outputs were high only when the theophylline Apta‐triggers were paired with the cognate switches with background level outputs for all the non‐matching Apta‐trigger and switch pairs (Figure 8b; Figure S19, Supporting Information). This indicates the characteristic orthogonality of toehold switch library can be maintained for Apta‐trigger designs such that unintended crosstalk between the components of START platform is minimized. Next, the specificity of different cognate Apta‐trigger and switch pairs toward input ligands was tested (Figure 8c). The Apta‐trigger and switch pairs were activated only in the presence of its cognate input ligand (Figure 8d). The presence of non‐cognate ligands resulted in low fluorescence output signals similar to the controls without the ligand input (Figure S20, Supporting Information). Together, the START design demonstrated good orthogonality for the trigger sequences and input ligands by taking advantage of the orthogonality of toehold switch library and the specificity of aptamers, providing a chassis for implementing more complex genetic circuits.

### 2.7 Construction of Boolean Logic Circuits with START

Based on the characteristic sequence flexibility and orthogonality, the START design may provide a synthetic riboregulator platform for more intricate genetic circuits with multiple ligands as input signals. As a proof of concept, we designed and tested Boolean logic gates with the START components for theophylline and MS2 coat protein in *E. coli*.

**Figure 9:**
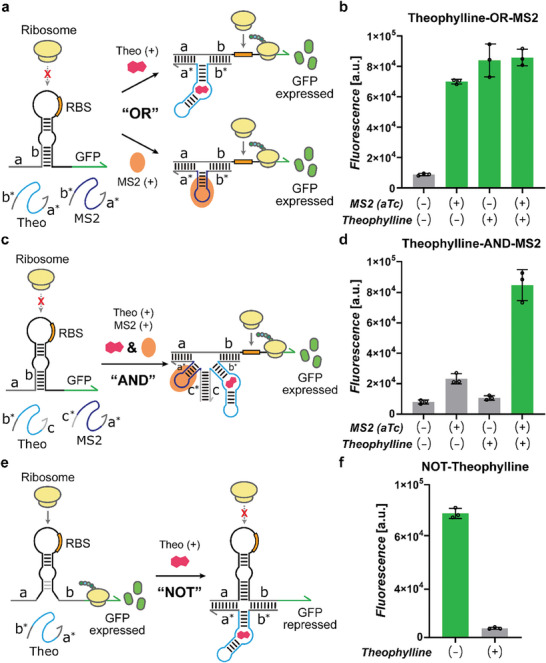
Construction of Boolean logic circuits. a) Design of the two‐input OR gate for theophylline and MS2. b) GFP fluorescence output of the two‐input OR gate for different input combinations. c) Design of the two‐input AND gate for theophylline and MS2. d) GFP fluorescence output of the two‐input AND gate for different input combinations. e) Design of the NOT gate for theophylline using a 3WJ repressor. f) GFP fluorescence of the NOT gate in the absence and presence of theophylline input. Theophylline was treated at a concentration of 10 mм, and aTc was treated at a concentration of 10 ng mL^−1^. Error bars represent the SD from three biological replicates.

To implement an OR gate, we employed theophylline and MS2 Apta‐triggers with the same partial trigger sequences that target the switch B sequence (Theo_B1 and MS2_B1). In this design, the presence of theophylline or MS2 protein input would stabilize the otherwise unstructured aptamer domains within the triggers such that either trigger RNA could induce strand displacement of switch RNA in the presence of its cognate ligand to allow translation of the reporter protein (**Figure** **9**a). While the expression level was low without any ligand, the presence of either theophylline or MS2 protein led to high reporter gene expression, indicating a successful implementation of an OR logic gate with theophylline and MS2 protein as inputs (Figure 9b).

For an AND gate design, a modified Apta‐trigger design was used analogous to the previous AND logic circuit (Figure 2b).^[^ ^29^ ^]^ Theophylline and MS2 Apta‐triggers were repurposed to serve the role of each partial trigger: for instance, theophylline aptamer was flanked by one part of partial trigger (b*) and a complementary sequence (c) to the modified MS2 Apta‐trigger (Figure 9c; Figure S21a, Supporting Information). In this design, while a complex containing both theophylline and MS2 Apta‐triggers could be formed via c‐c* domain interaction, the partial trigger sequences (a* and b*) would not be in proximity due to the lack of stable aptamer structure formation in the absence of the respective cognate ligands. Hence, both ligands would be required to stabilize both sides of aptamers such that the partial trigger sequences would be co‐localized to effectively induce strand displacement in the target switch RNA. Indeed, the fluorescence output was high when both theophylline and MS2 protein inputs were present, confirming the successful implementation of an AND logic circuit (Figure 9d).

For a NOT logic circuit, we used a modified Apta‐trigger design together with a previously reported 3‐way junction (3WJ) repressor.^[^ ^48^ ^]^ The 3WJ repressor switch RNA takes the form of a toehold switch with an unstable hairpin structure such that the RBS and start codon are accessible to ribosome. However, upon binding of a cognate trigger RNA, the switch‐trigger RNA complex adopts a stable 3WJ structure, sequestering the RBS. We modified the partial trigger domains of the theophylline Apta‐trigger to take the corresponding parts of the 3WJ trigger RNA sequences such that the stable switch‐trigger complex can suppress the translation of downstream gene in the presence of theophylline input (Figure 9e; Figure S21b, Supporting Information). In vivo characterization revealed that the expression of GFP was strongly suppressed in the presence of theophylline as desired (Figure 9f).

In summary, we successfully demonstrated Boolean logic operations with START platform, suggesting that this design approach can be seamlessly integrated into synthetic biological circuit designs and ribocomputing devices.

## 3 Discussion

The START design can offer several advantageous features in comparison with the conventional riboswitches. First, START demonstrates the design flexibility with little sequence constraints, making it compatible with a diverse set of genetic contexts. Second, a number of aptamer sequences can be embedded in Apta‐trigger designs for controlling synthetic toehold switches with little to no sequence adjustments, simplifying the overall design process while harnessing the large library of synthetic riboregulators. Third, START provides tunable dynamic and operational ranges for the target ligand via rational domain engineering without an extensive screening process. Finally, Apta‐trigger designs could be compatible with other RNA‐based regulatory systems in principle to regulate synthetic transcriptional switches^[^ ^49^ ^]^ and conditional guide RNAs.^[^ ^50^ ^]^

Despite the advantages in design flexibility, modularity, and tunability of START design presented here, there are certain limitations for the trans‐acting design architecture. First, increasing the number of circuit components in the trans‐acting design can escalate the host burden to realize synthetic gene circuits via the consumption of cellular resources and sharing of essential machinery.^[^ ^51^ ^]^ The circuit‐host interaction is an important aspect for the START platform, where transcription of switch and trigger RNAs and translation regulated by toehold switch are intricately intertwined for system performance, requiring further characterization and optimization. Second, the sensor module and the effector module are not co‐localized in the trans‐acting system, leading to a delay in response time without sufficiently high local concentrations.^[^ ^52^ ^]^ Third, trans‐acting components have potential off‐target effects via unintended interactions with biomolecular networks of host,^[^ ^52^ ^]^ requiring careful consideration to minimize such crosstalks. The cis‐acting design of START (Figure 6) may provide an alternative to alleviate these potential challenges. With further optimization in the linker and partial trigger sequence domains, the cis‐acting design variants may also provide a promising biosensor platform capitalizing on the benefits of cis‐acting devices.

While a trend was observed for START designs where a slightly weaker stem modification of aptamer domains led to functional Apta‐triggers, challenges remain for a general application of design and optimization for STARTs with different aptamers. One of the difficulties arises due to the availability of multiple thermodynamic parameters and in silico prediction tools, where it is challenging to provide a standard when the predicted values have discrepancy. As an example, tetracycline aptamers showed a relatively large discrepancy for calculated thermodynamic parameters (Note S2, Supporting Information). In particular, these in silico tools could be less accurate for those aptamers with pseudoknots and G‐quadruplexes. Further, the binding constants for aptamer and ligand interaction, or interaction domains are poorly defined in some cases. Thus, some empirical optimization steps are likely necessary for adopting new aptamer domains for START designs. Still, the advancement in in silico analysis tools as well as experimental techniques for selecting structure‐switching aptamers with modified SELEX^[^ ^53^ ^]^ and screening with structures analogous to aptamer‐beacons,^[^ ^54^ ^]^ could provide useful tools for expediting the development of suitable aptamers for application in START designs.

Natural and synthetic riboswitches are widely employed in diverse disciplines encompassing conditional gene regulation,^[^ ^55^ ^]^ biosensor development,^[^ ^56^ ^]^ metabolic flux engineering,^[^ ^57^ ^]^ strain selection,^[^ ^58^ ^]^ and therapeutics.^[^ ^59^ ^]^ The characteristic design flexibility and tunability of the START design presented here could provide a new platform to engineer riboswitches with designable dynamic and operating range for a broad range of target ligands in a plug‐and‐play manner. Together with the rapid development of synthetic RNA regulatory elements and expanding aptamer repertoires by the SELEX technique, the START designs could be repurposed to regulate a number of synthetic RNA‐based circuits.

## 4 Conclusion

In this study, we devised a synthetic biosensor platform with programmable performances, termed START, by encoding an aptamer sequence within input RNA sequences for synthetic riboregulators. This trans‐acting design applied to a toehold switch library demonstrated novel biosensors that respond to theophylline, tetracycline, and MS2 coat protein for a number of different sequence contexts. Systematic sequence analysis allowed rational adjustments of dynamic range and threshold for the START designs. We further implemented OR, AND and NOT Boolean logic circuits for multiple ligand inputs, highlighting the characteristic design flexibility and modularity of STARTs. We anticipate that our platform will present a new starting point for synthetic biosensor development, paving the way for versatile applications in RNA synthetic biology and bioengineering.

## 5 Experimental Section

### Bacterial Strains and Chemicals

The following *E. coli* strains were used in this study: DH5α (endA1 recA1 gyrA96 thi‐1 glnV44 relA1 hsdR17 (rK‐ mK+) λ‐) for cloning plasmids, and BL21 DE3 (F‐ ompT hsdSB (rB‐ mB‐) gal dcm [DE3]) for protein expression. All strains were grown in LB medium at 37 °C with appropriate antibiotics: ampicillin (100 µg mL^−1^) (Gold Biotechnology, USA, A‐301‐5), kanamycin (50 µg mL^−1^) (Gold Biotechnology, USA, K‐120‐25), spectinomycin (100 µg mL^−1^) (Gold Biotechnology, USA, S‐140‐25), and chloramphenicol (25 µg mL^−1^) (Gold Biotechnology, USA, C‐105‐5). The following chemicals were used in this study as ligands: theophylline (Sigma‐Aldrich, USA, T1633), tetracycline (Sigma‐Aldrich, USA, T3258), and aTc (Takara, Japan, 631310). Theophylline was dissolved in LB medium at a concentration of 10 mм, filtered, and diluted in fresh LB medium for immediate use. The stock solution of tetracycline was prepared at a concentration of 12 mg mL^−1^ in pure ethanol, filtered, and stored at −20 °C. The stock solution of aTc was provided at a concentration of 2 mg mL^−1^ by the vendor, and stored at −20 °C.

### In Silico Design and Analysis

For the computer‐based structural prediction and sequence design of RNA constructs, NUPACK^[^ ^40^ ^]^ 4.0 Python module and Cloud tools were utilized. For design and analysis of RNA sequences, the free energies were calculated with the following parameters: a temperature of 37 °C, free energy parameters of Mathews^[^ ^60^ ^]^ and Lu^[^ ^61^ ^]^ with additional parameters including coaxial stacking and dangle stacking,^[^ ^60^, ^62^ ^]^ 1.0 м Na^+^ and 0 м Mg^2+^. The RNA sequences, desired secondary structures for the complexes of the toehold switches and triggers were specified according to the reported toehold switch library.^[^ ^29^ ^]^ Structures of the aptamers were specified based on previous reports,^[^ ^36^, ^37^, ^45^, ^46^ ^]^ and further verified with the secondary structure prediction of NUPACK analysis tool. Calculated free‐energy of the original aptamers with 3‐bases of linear linker sequences were defined as ΔG_original_, and the free‐energy difference with sequence‐modified aptamers (ΔΔG = ΔG_modified_ – ΔG_original_) was employed for thermodynamic analysis. Sample codes for NUPACK designs with detailed descriptions were reported in Note S1 (Supporting Information).

### Plasmid Construction

Plasmids used in this study were constructed using PCR and Gibson assembly.^[^ ^63^ ^]^ The DNA sequences coding for switch and trigger RNAs were PCR amplified with partially overlapped single‐stranded DNAs as templates, and the plasmid backbones were taken from the commercial vectors pET15b, pCOLADuet, pCDFDuet, and pACYCDuet (Merck Millipore, Germany). All the PCR primers were purchased from Bionics (Korea), and PCR was performed using 2X Pfu PCR Master Mix (Coregen, Korea, PPI21S02). The PCR products were digested with DpnI (Enzynomics, Korea, R054) to remove plasmid templates, and further purified using a PCR clean‐up kit (GeneAll, Korea, 103‐102). The resulting DNAs were then Gibson‐assembled using 20–30 bp homology domains via Gibson assembly with 2X Gibson Assembly Master Mix (New England Biolabs, USA, E2611). The tetM expression plasmid for tetracycline resistance^[^ ^64^ ^]^ was purchased from Addgene (pMflT‐o4, #101312), and the plasmid was used without further modification. The MS2 protein coding sequence was PCR amplified using the bacteriophage MS2 coat protein expression plasmid from Addgene (pHMM, #67717) as a template and inserted into a pACYCDuet plasmid with an aTc inducible promoter sequence via Gibson assembly.^[^ ^65^ ^]^ All plasmids were cloned in *E. coli* DH5α strain and validated through DNA sequencing (Bionics, Korea). Plasmid sequences used in this study were provided in Tables S1–S3 (Supporting Information).

### Evaluation of STARTs

For in vivo characterization of STARTs, plasmids encoding switch RNA, trigger RNA, and other accessory plasmids were co‐transformed into *E. coli* BL21 DE3 strain. Unless otherwise noted, the switch and trigger RNAs were expressed with the T7 RNA polymerase induced with the addition of IPTG. Cells were screened on LB‐agar plates with appropriate antibiotics, and three colonies were picked for each condition. Each colony was grown overnight in 6 mL LB medium with antibiotics with shaking at 250 rpm and 37 °C. Cells were then diluted 1/100‐fold into fresh 1 mL LB medium with antibiotics in a 96‐well plate with specific concentration of target ligands (i.e., theophylline, tetracycline, or aTc), and returned to the shaker (1000 rpm, 37 °C). After 80 minutes, the transcription of the switch and trigger RNAs were induced with 0.1 mм IPTG. Cells were returned to the shaker (1000 rpm, 37 °C), until measurements on the cell cultures were taken 3 hours and 30 minutes after the addition of IPTG. For tetracycline input, measurements were taken 5 hours and 30 minutes after the IPTG addition to compensate for reduced growth rate due to antibiotic ligand treatment.

### Flow Cytometry Measurements and Analysis

A 200 µL of induced cells were fixed with 2% paraformaldehyde in 1X phosphate‐buffered saline (PBS),^[^ ^66^ ^]^ washed, and diluted in 1X PBS (typically ≈ 1/5‐fold). Flow cytometry was performed using a CytoFLEX LX (Beckman Coulter, USA) equipped with a standard well plate loader. Forward scatter (FSC) and side scatter (SSC) were used as primary and secondary trigger respectively, with AND logic operator, and ≈ 50000 individual cells per each biological replicate were recorded. Cell populations were gated according to their FSC and SSC distributions as described previously,^[^ ^67^ ^]^ and the geometric mean was employed to extract the average fluorescence from the approximately log‐normal fluorescence distribution. Gating and fluorescence data analysis were conducted using CytExpert software (Beckman Coulter, USA). Errors were analyzed with standard deviations calculated from the three biological replicates.

### Plate Reader Measurements and Analysis

For the time‐course measurements of STARTs with constitutive promoters, BioTek Synergy H1 microplate reader (Agilent, USA) measurement was used instead. The overnight culture was diluted 1/100‐fold for plate reader measurement. The culture volume was 200 µL in a 96‐well plate (SPL Life Sciences, Korea, 33396), and the number of replicates was three for each condition with the addition of inducers after 2 hours. At 15 minute intervals, the cell growth was monitored by absorbance at 600 nm (OD_600_) and GFP fluorescence was measured using a monochromator with excitation wavelength at 479 nm and emission wavelength at 520 nm. Sample temperature was maintained at 37 °C. Fluorescence data were normalized by dividing GFP fluorescence with OD_600_ value. Errors were analyzed with standard deviations calculated from the three biological replicates.

## Conflict of Interest

The authors declare no conflict of interest.

## Supporting information

Supporting Information

## Data Availability

The data that support the findings of this study are available from the corresponding author upon reasonable request.

## References

[1] G. M. Edelman , J. A. Gally , Proc. Natl. Acad. Sci. USA 2001, 98, 13763. 11698650 10.1073/pnas.231499798 PMC61115

[2] J. J. Tyson , K. C. Chen , B. Novak , Curr. Opin. Cell Biol. 2003, 15, 221. 12648679 10.1016/s0955-0674(03)00017-6

[3] a) D. S. Latchman , Int. J. Biochem. Cell Biol. 1997, 29, 1305; 9570129 10.1016/s1357-2725(97)00085-x

[4] a) H. Alper , J. Moxley , E. Nevoigt , G. R. Fink , G. Stephanopoulos , Science 2006, 314, 1565; 17158319 10.1126/science.1131969

[5] M. Leisola , O. Turunen , Appl. Microbiol. Biotechnol. 2007, 75, 1225. 17404726 10.1007/s00253-007-0964-2

[6] a) M. E. Fris , E. R. Murphy , Front Cell Infect Microbiol 2016, 6, 2. 26858941 10.3389/fcimb.2016.00002 PMC4728522

[7] a) J. Krützfeldt , M. Stoffel , Cell Metab. 2006, 4, 9; 16814728 10.1016/j.cmet.2006.05.009

[8] a) P. P. Amaral , J. S. Mattick , Mamm Genome 2008, 19, 454; 18839252 10.1007/s00335-008-9136-7

[9] a) S. Carpenter , D. Aiello , M. K. Atianand , E. P. Ricci , P. Gandhi , L. L. Hall , M. Byron , B. Monks , M. Henry‐Bezy , J. B. Lawrence , L. A. O'Neill , M. J. Moore , D. R. Caffrey , K. A. Fitzgerald , Science 2013, 341, 789; 23907535 10.1126/science.1240925 PMC4376668

[10] a) J. Mulhbacher , D. A. Lafontaine , Nucleic Acids Res. 2007, 35, 5568; 17704135 10.1093/nar/gkm572 PMC2018637

[11] a) F. J. Isaacs , D. J. Dwyer , C. Ding , D. D. Pervouchine , C. R. Cantor , J. J. Collins , Nat. Biotechnol. 2004, 22, 841; 15208640 10.1038/nbt986

[12] J. T. Stevens , J. M. Carothers , ACS Synth. Biol. 2015, 4, 107. 25314371 10.1021/sb400201u

[13] E. A. Davidson , A. D. Ellington , Nat. Chem. Biol. 2007, 3, 23. 17173026 10.1038/nchembio846

[14] a) C. C. Fowler , Y. Li , Methods Protoc 2014, 1103, 177; 10.1007/978-1-62703-730-3_14 24318895

[15] a) C. Laing , T. Schlick , Curr. Opin. Struct. Biol. 2011, 21, 306; 21514143 10.1016/j.sbi.2011.03.015 PMC3112238

[16] K. Sato , M. Akiyama , Y. Sakakibara , Nat. Commun. 2021, 12, 941. 33574226 10.1038/s41467-021-21194-4 PMC7878809

[17] R. R. Breaker , Cold Spring Harb Perspect Biol 2018, 10, a032797. 29844057 10.1101/cshperspect.a032797 PMC6211393

[18] R. Rieder , K. Lang , D. Graber , R. Micura , ChemBioChem 2007, 8, 896. 17440909 10.1002/cbic.200700057

[19] K. Hollands , S. Proshkin , S. Sklyarova , V. Epshtein , A. Mironov , E. Nudler , E. A. Groisman , Proc. Natl. Acad. Sci. USA 2012, 109, 5376. 22431636 10.1073/pnas.1112211109 PMC3325659

[20] A. D. Garst , A. L. Edwards , R. T. Batey , Cold Spring Harb. Perspect. Biol. 2011, 3, 003533. 10.1101/cshperspect.a003533 PMC3098680 20943759

[21] J. Miranda‐Ríos , M. Navarro , M. Soberón , Proc. Natl. Acad. Sci. USA 2001, 98, 9736. 11470904 10.1073/pnas.161168098 PMC55522

[22] a) W. C. Winkler , A. Nahvi , N. Sudarsan , J. E. Barrick , R. R. Breaker , Nat Struct Biol 2003, 10, 701; 12910260 10.1038/nsb967

[23] J. N. Kim , A. Roth , R. R. Breaker , Proc. Natl. Acad. Sci. USA 2007, 104, 16092. 17911257 10.1073/pnas.0705884104 PMC1999398

[24] a) K. Furukawa , A. Ramesh , Z. Zhou , Z. Weinberg , T. Vallery , W. C. Winkler , R. R. Breaker , Mol. Cell 2015, 57, 1088; 25794617 10.1016/j.molcel.2015.02.009 PMC4667775

[25] E. M. Aghdam , M. S. Hejazi , A. Barzegar , Gene 2016, 592, 244. 27432066 10.1016/j.gene.2016.07.035

[26] L. B. Zhou , A. P. Zeng , ACS Synth. Biol. 2015, 4, 729. 25575181 10.1021/sb500332c

[27] J. Mulhbacher , P. St‐Pierre , D. A. Lafontaine , Curr. Opin. Pharmacol. 2010, 10, 551. 20685165 10.1016/j.coph.2010.07.002

[28] A. A. Green , P. A. Silver , J. J. Collins , P. Yin , Cell. 2014, 159, 925. 25417166 10.1016/j.cell.2014.10.002 PMC4265554

[29] A. A. Green , J. Kim , D. Ma , P. A. Silver , J. J. Collins , P. Yin , Nature 2017, 548, 117. 28746304 10.1038/nature23271 PMC6078203

[30] H. Ge , M. A. Marchisio , Life 2021, 11, 248. 33802772 10.3390/life11030248 PMC8002509

[31] A. Peselis , A. Serganov , Biochim. Biophys. Acta 2014, 1839, 908. 24583553 10.1016/j.bbagrm.2014.02.012 PMC4643838

[32] a) T. S. Bayer , C. D. Smolke , Nat. Biotechnol. 2005, 23, 337; 15723047 10.1038/nbt1069

[33] Y. Liu , J. Li , Z. Chen , W. Huang , Z. Cai , Elife 2018, 7, e31936. 29319503 10.7554/eLife.31936 PMC5788502

[34] a) J. Lloyd , C. H. Tran , K. Wadhwani , C. Cuba Samaniego , H. K. K. Subramanian , E. Franco , ACS Synth. Biol. 2018, 7, 30; 29028334 10.1021/acssynbio.7b00277

[35] A. T. Catherine , S. N. Shishido , G. A. Robbins‐Welty , A. Diegelman‐Parente , FEBS Open Bio 2014, 4, 788. 10.1016/j.fob.2014.08.008 PMC4209343 25352996

[36] A. Wrist , W. Sun , R. M. Summers , ACS Synth. Biol. 2020, 9, 682. 32142605 10.1021/acssynbio.9b00475

[37] G. R. Zimmermann , C. L. Wick , T. P. Shields , R. D. Jenison , A. Pardi , RNA 2000, 6, 659. 10836787 10.1017/s1355838200000169 PMC1369946

[38] H. Gouda , I. D. Kuntz , D. A. Case , P. A. Kollman , Biopolymers 2003, 68, 16. 12579577 10.1002/bip.10270

[39] J. Horne , E. Beddingfield , M. Knapp , S. Mitchell , L. Crawford , S. B. Mills , A. Wrist , S. Zhang , R. M. Summers , ACS Omega 2020, 5, 32250. 33376862 10.1021/acsomega.0c03909 PMC7758883

[40] J. N. Zadeh , C. D. Steenberg , J. S. Bois , B. R. Wolfe , M. B. Pierce , A. R. Khan , R. M. Dirks , N. A. Pierce , J. Comput. Chem. 2011, 32, 170. 20645303 10.1002/jcc.21596

[41] Y. Nakahira , A. Ogawa , H. Asano , T. Oyama , Y. Tozawa , Plant Cell Physiol. 2013, 54, 1724. 23969558 10.1093/pcp/pct115

[42] a) D. F. Browning , R. E. Godfrey , K. L. Richards , C. Robinson , S. J. W. Busby , Biochem. Soc. Trans. 2019, 47, 755; 30971435 10.1042/BST20190059

[43] a) C. Rangel‐Chavez , E. Galan‐Vasquez , A. Martinez‐Antonio , Mol. BioSyst. 2017, 13, 665; 28256660 10.1039/c6mb00789a

[44] V. A. Rhodius , T. H. Segall‐Shapiro , B. D. Sharon , A. Ghodasara , E. Orlova , H. Tabakh , D. H. Burkhardt , K. Clancy , T. C. Peterson , C. A. Gross , C. A. Voigt , Mol Syst Biol 2013, 9, 702. 24169405 10.1038/msb.2013.58 PMC3817407

[45] a) S. Hanson , G. Bauer , B. Fink , B. Suess , RNA 2005, 11, 503; 15769877 10.1261/rna.7251305 PMC1370739

[46] L. Qi , J. B. Lucks , C. C. Liu , V. K. Mutalik , A. P. Arkin , Nucleic Acids Res. 2012, 40, 5775. 22383579 10.1093/nar/gks168 PMC3384300

[47] a) E. Bertrand , P. Chartrand , M. Schaefer , S. M. Shenoy , R. H. Singer , R. M. Long , Mol. Cell 1998, 2, 437; 9809065 10.1016/s1097-2765(00)80143-4

[48] J. Kim , Y. Zhou , P. D. Carlson , M. Teichmann , S. Chaudhary , F. C. Simmel , P. A. Silver , J. J. Collins , J. B. Lucks , P. Yin , A. A. Green , Nat. Chem. Biol. 2019, 15, 1173. 31686032 10.1038/s41589-019-0388-1 PMC6864284

[49] a) J. Chappell , A. Westbrook , M. Verosloff , J. B. Lucks , Nat. Commun. 2017, 8, 1051; 29051490 10.1038/s41467-017-01082-6 PMC5648800

[50] a) M. H. Hanewich‐Hollatz , Z. Chen , L. M. Hochrein , J. Huang , N. A. Pierce , ACS Cent. Sci. 2019, 5, 1241; 31403072 10.1021/acscentsci.9b00340 PMC6661866

[51] a) R. F. Harvey , T. S. Smith , T. Mulroney , R. M. L. Queiroz , M. Pizzinga , V. Dezi , E. Villenueva , M. Ramakrishna , K. S. Lilley , A. E. Willis , Wiley Interdiscip Rev RNA 2018, 9, e1465; 29341429 10.1002/wrna.1465 PMC5947564

[52] G. Domin , S. Findeiß , M. Wachsmuth , S. Will , P. F. Stadler , M. Mörl , Nucleic Acids Res. 2017, 45, 4108. 27994029 10.1093/nar/gkw1267 PMC5397205

[53] a) A. A. Sanford , A. E. Rangel , T. A. Feagin , R. G. Lowery , H. S. Argueta‐Gonzalez , J. M. Heemstra , Chem. Sci. 2021, 12, 11692; 34659704 10.1039/d1sc02715h PMC8442683

[54] a) N. Hamaguchi , A. Ellington , M. Stanton , Anal. Biochem. 2001, 294, 126; 11444807 10.1006/abio.2001.5169

[55] M. M. Rudolph , M. P. Vockenhuber , B. Suess , Methods Enzymol 2015, 550, 283. 25605391 10.1016/bs.mie.2014.10.036

[56] S. Manna , J. Truong , M. C. Hammond , ACS Synth. Biol. 2021, 10, 566. 33646758 10.1021/acssynbio.0c00583 PMC7985839

[57] H. G. Hwang , M. H. Noh , M. A. G. Koffas , S. Jang , G. Y. Jung , Metab. Eng. 2021, 67, 417. 34416365 10.1016/j.ymben.2021.08.003

[58] a) S. Jang , J. Yang , S. W. Seo , G. Y. Jung , Methods Enzymol 2015, 550, 341; 25605394 10.1016/bs.mie.2014.10.039

[59] Z. J. Tickner , M. Farzan , Pharmaceuticals 2021, 14, 554. 34200913 10.3390/ph14060554 PMC8230432

[60] D. H. Mathews , J. Sabina , M. Zuker , D. H. Turner , J. Mol. Biol. 1999, 288, 911. 10329189 10.1006/jmbi.1999.2700

[61] Z. J. Lu , D. H. Turner , D. H. Mathews , Nucleic Acids Res. 2006, 34, 4912. 16982646 10.1093/nar/gkl472 PMC1635246

[62] a) T. Xia , J. SantaLucia Jr. , M. E. Burkard , R. Kierzek , S. J. Schroeder , X. Jiao , C. Cox , D. H. Turner , Biochemistry 1998, 37, 14719; 9778347 10.1021/bi9809425

[63] D. G. Gibson , L. Young , R. Y. Chuang , J. C. Venter , C. A. 3rd Hutchison , H. O. Smith , Nat. Methods 2009, 6, 343. 19363495 10.1038/nmeth.1318

[64] D. Matteau , M. E. Pepin , V. Baby , S. Gauthier , M. Arango Giraldo , T. F. Knight , S. Rodrigue , Appl. Environ. Microbiol. 2017, 83, e03374. 28115382 10.1128/AEM.03374-16 PMC5359490

[65] R. Lutz , H. Bujard , Nucleic Acids Res. 1997, 25, 1203. 9092630 10.1093/nar/25.6.1203 PMC146584

[66] M. M. Varela , H. M. van Aken , E. Sintes , T. Reinthaler , G. J. Herndl , Environ. Microbiol. 2011, 13, 1524. 21418496 10.1111/j.1462-2920.2011.02457.x

[67] A. I. Konokhova , A. A. Gelash , M. A. Yurkin , A. V. Chernyshev , V. P. Maltsev , Cytometry, Part A 2013, 83, 568. 10.1002/cyto.a.22294 23568828

