# Supplementary material for: START: A Versatile Platform for Bacterial Ligand Sensing with Programmable Performances
Source: Adv Sci (Weinh). 2024 Jul 29;11(36):2402029. doi: 10.1002/advs.202402029 (PMC11423158; doi:10.1002/advs.202402029)
Supplement: Supplementary file 1 — Supporting Information [file ADVS-11-2402029-s001.pdf]

ADVANCED SCIENCE
Open Access

Supporting Information

for *Adv. Sci.*, DOI 10.1002/advs.202402029

START: A Versatile Platform for Bacterial Ligand Sensing with Programmable Performances

*Jeongwon Kim, Minchae Seo, Yelin Lim and Jongmin Kim**

Supporting Information

START: A Versatile Platform for Bacterial Ligand Sensing with Programmable Performances

*Jeongwon Kim, Minchae Seo, Yelin Lim, and Jongmin Kim**

* jongmin.kim@postech.ac.kr

Supplementary Notes 2

Note S1. In silico sequence analysis and design with NUPACK 2

Note S2. Design and optimization of START constructs with thermodynamic analysis 4

Supplementary Tables 6

Table S1. Construct architectures 6

Table S2. Examples of construct DNA sequences 8

Table S3. Module sequences 12

Supplementary Figures 21

Figure S1. Comparative analysis with nonsense RNA construct 21

Figure S2. Growth curves of *E. coli* strain BL21 with ligand treatment 21

Figure S3. GFP fluorescence with theophylline Apta-trigger Theo_A1 22

Figure S4. Dose-response curves of stem-modified theophylline Apta-triggers 22

Figure S5. Design optimization of partial trigger domains for dynamic range improvement 23

Figure S6. Optimized theophylline Apta-triggers and their dynamic range 24

Figure S7. Flow cytometry histograms with the optimized theophylline Apta-triggers 25

Figure S8. Comparative analysis with cis-acting theophylline riboswitch 26

Figure S9. START characterization with different promoters 27

Figure S10. START characterization with different ribosome binding sites 28

Figure S11. Theophylline START constructs utilizing toehold switch library 29

Figure S12. Characterization of theophylline Apta-triggers with different plasmid copy number 29

Figure S13. START characterization in *E. coli* strain Nissle 1917 30

Figure S14. Design and characterization of tetracycline STARTs 31

Figure S15. GFP positive control with tetracycline treatment 32

Figure S16. In vivo expression of MS2 coat protein 33

Figure S17. Design and characterization of MS2 STARTs 34

Figure S18. Characterization of MS2 START constructs across different genetic contexts 35

Figure S19. Orthogonality between switch RNA and partial trigger domains 36

Figure S20. Orthogonality between STARTs and ligands 37

Figure S21. Predicted secondary structures of logic gate complex 37

References 38

Supplementary Notes

Note S1. In silico sequence analysis and design with NUPACK

In this study, in silico structure prediction, free-energy calculation, and sequence design of RNA constructs were conducted with NUPACK[1] 4.0 Python module in Linux and Web version cloud tools (https://nupack.org/). The following parameters were used identically for sequence analysis and design: a temperature of 37 °C, rna06-free energy parameters of Mathews et al.[2] and Lu et al.[3] with additional parameters including coaxial stacking and dangle stacking,[2, 4] 1.0 M Na^{+} and 0 M Mg^{2+}.

The design of Apta-trigger went through the following process. First, we selected target aptamers whose sequence and structure were reported in the literature, and verified its secondary structure with NUPACK analysis tool to identify binding pocket and lower stem domains. Then, we simulated sequence interactions between the aptamer and switch-trigger among the toehold switch library[5] to select proper candidates without unwanted interactions. The aptamer sequence was inserted at the position that bisects the 26-base trigger sequence into 13-bases, and additional 3-base linear linker sequences were inserted between each partial trigger and lower stem sequence. Since the NUPACK cannot predict ligand-induced conformational change, both structures with and without stable lower stem duplex were assumed to be the target structures. The following script provides an example of theophylline Apta-trigger design with the lower stem sequences of 1-bp of G-C pairing and 3-bp of A-U pairing.

```
from nupack import *
from datetime import datetime
import time

config.parallelism = True

# Define physical model
my_model = Model(material='rna06', celsius=37)

# Define sequence domains
start = Domain('G', name='start')
tr_stemloop = Domain('AUACACAUAGAAUCAUGUGUAU', name='tr_stem')
tr_linA = Domain('AAC', name='tr_linA')
tr_A = Domain('ACUACUAAACUUC', name='tr_A')
apt_linA = Domain('R3', name='apt_linA')
theo_stem = Domain('S1W3', name='theo_stem')
theo_apt = Domain('AUACCAGCAUCGUCUUGAUGCCCUUGGCAG', name='theo_apt')
apt_linB = Domain('R3', name='apt_linB')
tr_B = Domain('UAUCAUAUUCAAU', name='tr_B')
tr_linB = Domain('CAC', name='tr_linB')

print('domain setting completed\n')

# Define strands
apta_tr = TargetStrand([start, tr_stemloop, tr_linA, tr_A, apt_linA, theo_stem, theo_apt,
~theo_stem, apt_linB, tr_B, tr_linB], name='apta_tr')

print('strand setting completed\n')

# Define 'target' complex
APTA_TR = TargetComplex([apta_tr],
'.((((((((......))))))))...................((((...((((((((.....)))))...)))...))))...................', name='APTA_TR')
```

```
APTA_TR_NOSTEM = TargetComplex([apta_tr],
'.((((((((......))))))))..........................((((((((.....)))))...)))..........................', name='APTA_TR')

print('complex setting completed\n')

# Define prevent pattern
pattern1 = Pattern(['A5', 'C5', 'G5', 'U5', 'M6', 'K6', 'W6', 'S6', 'R6', 'Y6'])

print('options setting completed\n')

# Define a target test tube
tube_APTA_TR = TargetTube(on_targets = {APTA_TR: 1e-7}, off_targets =
SetSpec(max_size=2, exclude=[APTA_TR_NOSTEM]), name='tube_APTA_TR')

print('test_tube setting completed\n')

# Tube weight setting
my_tubes = [tube_APTA_TR]
weights = Weights(my_tubes)

weights[:, :, :, tube_APTA_TR] = 200

trial_n = 1

current_time = time.time()
seed_int = int(current_time)
my_options = DesignOptions(seed=seed_int)
my_design = tube_design(tubes=my_tubes, model=my_model, soft_constraints=[pattern1],
defect_weights=weights)
my_jobs = my_design.launch(trials=trial_n)
my_jobs.wait()
my_final_results = my_jobs.final_results()

for i in range(trial_n):
    print('\n#############number %d#############\n' % (i+1))
    print('\n---- Domain & Sequences ----\n')
    print(my_final_results[i].to_analysis)
    print('\n---- On_target & Off_target complex concentrations ----\n')
    print(my_final_results[i].concentrations)
    print('\n')

print("design done")

print('JOB DONE\n')
```

Note S2. Design and optimization of START constructs with thermodynamic analysis

To start, we briefly review the mechanism of toehold switch and trigger interactions, which form the basis of START constructs. For toehold switch designs, a switch RNA suppresses gene expression by sequestering the RBS and start codon within its stem-loop structure. When a trigger RNA binds to the toehold switch through a complementary sequence domain, it induces strand-displacement, releasing the RBS and start codon to allow active translation of the downstream gene. Previous studies have reported that the strand displacement and activation of toehold switch RNA rarely occurs when each partial cognate trigger is present, while the strand-displacement proceeds efficiently when they form a stable trigger complex through complementary sequence domains.[5] Inserting an aptamer domain within the split trigger RNA may still suppress strand-displacement by keeping each part of trigger in a distance, whereas a conformational change upon ligand binding can stabilize partial triggers in close proximity for effective activation of the switch RNA. Thus, the aptamer domain should ideally form a stable ligand-bound structure, bringing the partial trigger domains into close proximity in the presence of the ligand while remaining largely unstructured in the absence of ligand input.

For new aptamer candidates to be incorporated in START designs, broadly, there can be two failure modes. In one scenario, the switch would be activated by Apta-triggers in the absence of ligand, indicating a stable aptamer structure formation in the absence of ligand. In the other scenario, the switch would not be activated by Apta-triggers even in the presence of ligand, indicating an unstructured aptamer unable to change conformation. In the second case of weak aptamer structure, the aptamer domains could be extended to stabilize the structure. Still, there is no guarantee that the stabilized structure would be capable of structural switching. For well-characterized aptamers, such as theophylline and tetracycline aptamers, we observed that the native aptamer structure was already sufficiently stable, corresponding to active Apta-triggers even in the absence of ligand. For these cases, the approach to destabilize the structure-stabilizing lower stem could be attempted without affecting the affinity of aptamer domains to ligands as described in the main text. A gradual decrease in the stability of aptamer lower stem domain could render the domain more unstable in the absence of ligand, reducing the formation of a stochastic binding-competent state. However, if the aptamer structure becomes too unstable, the active conformation may be insufficient to drive expression from toehold switch RNAs, potentially compromising the dynamic ranges (Figure 4a-c).

The important question for optimization of START performance, then, is to what extent the stability of aptamer domains should be tuned. To address this issue, we analyzed calculated thermodynamic energies for multiple stem-modified aptamer sequences using a number of in silico prediction tools.[1, 4d, 6] We observed that the predicted thermodynamic parameters and minimum-free-energy structures varied depending on the prediction tool used (Table N1). Notably, the predicted thermodynamic parameters differed by several kcal mol^{-1} for the tetracycline aptamer. This indicates that the thermodynamic parameters for the rather complex configurations of aptamer sequences involving stems, loops, and bulges still require further adjustments and improvements for accurate prediction in in silico tools. Moreover, the predicted minimum free-energy (MFE) structure and the ligand-bound conformation we specified based on the literature were different from each other with sometimes large energy gaps (Table N1 and Figure N1). The unique characteristics of each aptamer, such as G-quadruplex formation and pseudoknot interactions, could further reduce the accuracy of common in silico tools for RNA structure analysis. For these and other reasons, it is still challenging to provide a general guide for the use of in silico design tools and a standard energy range for optimal START performance.

Empirically, an aptamer lower stem with 3 to 5 bases mainly consisting of weak A-U base pairs was suitable for functional Apta-trigger designs. This empirical result correlates with other previous studies on modifying aptamers for structure switching capability.[7] While several in silico tools are available, we mainly used NUPACK for thermodynamic calculation, since NUPACK employs recently updated thermodynamic parameters and allows the calculation of energies for specified

structures. Further, NUPACK allows analysis for complexes with multiple strands, with the additional advantage of providing design functions that can be flexibly modified for design of new RNA devices such as Apta-triggers in STARTs.

Table N1. Free-energy prediction of stem-modified aptamers with in silico tools

[kcal mol^{-1}] Theophylline	NUPACK[1]	ViennaRNA[6a]	Mfold[4d]	RNAstructure[6b]
GCGC	-15.84* (-15.84)**	-16.00 (-16.00)	-16.50 (-16.50)	-16.20 (-16.20)
GGUG	-14.24 (-14.24)	-14.40 (-14.40)	-14.90 (-14.90)	-14.60 (-14.60)
UAUG	-10.07 (-10.07)	-10.30 (-10.30)	-10.20 (-10.20)	-10.30 (-10.30)
UAUU	-8.17 (-8.17)	-8.40 (-8.40)	-8.30 (-8.30)	-8.40 (-8.40)
UUA	-8.03 (-7.07)	-7.60 (-7.30)	-7.20 (-7.20)	-7.60 (-7.30)
UA	-8.03 (-6.17)	-7.60 (-6.40)	-7.10 (-6.30)	-7.60 (N/A)†
Tetracycline				
GGCCUA	-14.82 (-14.82)	-19.20 (-19.00)	-21.60 (-21.60)	-18.50 (-18.50)
GGCA	-13.06 (-10.22)	-15.10 (-14.40)	-17.70 (-17.70)	-14.10 (-13.90)
UCCA	-13.06 (-8.10)	-12.90 (-12.20)	-15.40 (-15.40)	-12.00 (-11.50)
CAUUA	-13.01 (-6.41)	-11.90 (-10.60)	-13.30 (-13.30)	-11.90 (N/A)
CUA	-13.01 (-4.41)	-11.90 (-8.60)	-12.70 (N/A)	-11.90 (N/A)
MS2				
AACAUG	-7.72 (-7.72)	-7.20 (-7.20)	-7.20 (-7.20)	-7.20 (-7.20)
ACAUG	-6.82 (-6.82)	-6.30 (-6.30)	-6.30 (-6.30)	-6.30 (-6.30)
AACAUU	-5.32 (-5.32)	-4.80 (-4.80)	-4.80 (-4.80)	-4.80 (-4.80)
AACAU	-4.42 (-4.42)	-3.90 (-3.90)	-3.90 (-3.90)	-3.90 (-3.90)
AAUG	-3.42 (-3.42)	-2.90 (-2.90)	-2.90 (-2.90)	-2.90 (-2.90)

* Minimum free-energy (MFE) prediction

** Free-energy prediction for specified ligand-bound state

(N/A)†: Unable to calculate free-energy for the specified structure

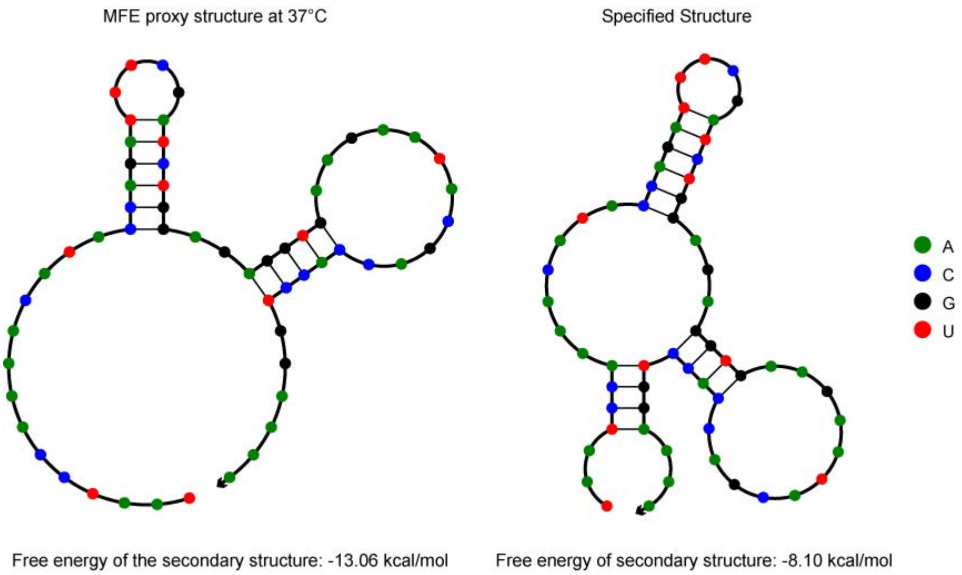

Figure N1. Secondary structure prediction of stem-modified tetracycline aptamer with NUPACK.

Predicted secondary structures and associated free-energies of stem-modified tetracycline aptamer sequence, Tetra_B3, were obtained with NUPACK analysis tool for minimum free-energy structure (left) and structure-specified ligand-bound conformation (right).

Supplementary Tables

Table S1. Construct architectures

Architectures of switch & trigger constructs used in this study. Abbreviations are as follows: THS = Toehold switch, Theo = Theophylline Apta-trigger, Tetra = Tetracycline Apta-trigger, MS2 = MS2 protein Apta-trigger, ScD = Scrambled decoy trigger, T7term = T7 terminator, 3WJ = 3-way junction repressor, KanR = Kanamycin resistance gene, AmpR = Ampicillin resistance gene, SpR = Spectinomycin resistance gene, ChlR = Chloramphenicol resistance gene, ColA = ColA replication origin, pBR322 = pBR322 replication origin, p15A = p15A replicate origin, CloDF13 = CloDF13 replication origin. For THS, six switch-trigger pairs were chosen based on their high fold-change values reported: ACTS_TypeII_N3 (THS A), ACTS_TypeII_N2 (THS B), ACTS_TypeII_N1 (THS C), ACTS_TypeII_N7 (THS D), ACTS_TypeI_N1 (THS F), ACTS_TYPEI_N2 (THS G).[5] Switches were renamed in the order they appeared in the main text. For 3WJ, single switch-trigger pair was chosen based on its high fold-change values reported: 3WJrep_N19 (3WJ A).[8]

Name	Plasmid architecture	Figure
**Switch**		
THS A	pT7-THS A-GFPmut3b-T7term-KanR-ColA-LacI	3, 4, 6, 8, 9, S1, S3, S4, S5, S6, S7, S8, S10, S11, S12, S19, S20, S21
THS A_B0030	pT7-THS A_B0030-GFPmut3b-T7term-KanR-ColA-LacI	S10
THS A_B0033	pT7-THS A_B0033-GFPmut3b-T7term-KanR-ColA-LacI	S10
THS A_B0034	pT7-THS A_B0034-GFPmut3b-T7term-KanR-ColA-LacI	S10
THS A_J61100	pT7-THS A_J61100-GFPmut3b-T7term-KanR-ColA-LacI	S10
THS A_J61125	pT7-THS A_J61125-GFPmut3b-T7term-KanR-ColA-LacI	S10
THS A_J61132	pT7-THS A_J61132-GFPmut3b-T7term-KanR-ColA-LacI	S10
THS A_ECF987	pT7-THS A-ECF987-T7term-KanR-ColA-LacI	5
THS B	pT7-THS B-GFPmut3b-T7term-KanR-ColA-LacI	6, 7, 8, 9, S9, S11, S14, S18, S19, S20
THS B_pLlacO	pLlacO-THS B-GFPmut3b-T7term-KanR-ColA-LacI	S9
THS B_pJ23119	pJ23119-THS B-GFPmut3b-T7term-KanR-ColA-LacI	S9, S13
THS C	pT7-THS C-GFPmut3b-T7term-KanR-ColA-LacI	7, 8, S11, S17, S18, S19, S20
THS C_pLlacO	pLlacO-THS C-GFPmut3b-T7term-KanR-ColA-LacI	S18
THS C_pJ23119	pJ23119-THS C-GFPmut3b-T7term-KanR-ColA-LacI	S18
THS C_mCherry	pT7-THS C-mCherry-T7term-KanR-ColA-LacI	S18
THS C_YFP	pT7-THS C-YFP-T7term-KanR-ColA-LacI	S18
THS C_Cerulean	pT7-THS C-Cerulean-T7term-KanR-ColA-LacI	S18
THS D	pT7-THS D-GFPmut3b-T7term-KanR-ColA-LacI	8, S11, S19

THS E	pT7-THS E-GFPmut3b-T7term-KanR-ColA-LacI	8, S19
THS F	pT7-THS F-GFPmut3b-T7term-KanR-ColA-LacI	8, S19
THS G	pT7-THS G-GFPmut3b-T7term-KanR-ColA-LacI	8, S19
3WJ A	pT7-3WJ A-GFPmut3b-T7term-KanR-ColA-LacI	9, S21
**THS trigger**		
THS Trigger A	pT7-THS Trigger A-T7term-AmpR-pBR322-LacI	3, 5, S1
THS Trigger A_nonsense	pT7-Nonsense-T7term-AmpR-pBR322-LacI	S1
THS Trigger B	pT7-THS Trigger B-T7term-AmpR-pBR322-LacI	7, S14
THS Trigger C	pT7-THS Trigger C-T7term-AmpR-pBR322-LacI	7, S17, S18
ScD	pT7-ScD-T7term-AmpR-pBR322-LacI	3, 5, 7, S1, S14, S17, S18
ScD_pLlacO	pLlacO-ScD-T7term-AmpR-pBR322-LacI	S9, S18
ScD_pJ23119	pJ23119-ScD-T7term-AmpR-pBR322-LacI	S9, S13, S18
**Apta-trigger**		
Theo_A1	pT7-Theo_A1-T7term-AmpR-pBR322-LacI	3, S3
Theo_A2	pT7-Theo_A2-T7term-AmpR-pBR322-LacI	3, S1, S4, S5, S6, S7, S8, S10
Theo_A2_shift1	pT7-Theo_A2_shift1-T7term-AmpR-pBR322-LacI	S5
Theo_A2_shift2	pT7-Theo_A2_shift2-T7term-AmpR-pBR322-LacI	S5
Theo_A2_shift3	pT7-Theo_A2_shift3-T7term-AmpR-pBR322-LacI	S5
Theo_A2_shift4	pT7-Theo_A2_shift4-T7term-AmpR-pBR322-LacI	S5
Theo_A2_shift5	pT7-Theo_A2_shift5-T7term-AmpR-pBR322-LacI	S5
Theo_A2_shift6	pT7-Theo_A2_shift6-T7term-AmpR-pBR322-LacI	S5
Theo_A2_trim1	pT7-Theo_A2_trim1-T7term-AmpR-pBR322-LacI	S5
Theo_A2_trim2	pT7-Theo_A2_trim2-T7term-AmpR-pBR322-LacI	S5
Theo_A2_trim3	pT7-Theo_A2_trim3-T7term-AmpR-pBR322-LacI	S5
Theo_A2_trim4	pT7-Theo_A2_trim4-T7term-AmpR-pBR322-LacI	S5
Theo_A3	pT7-Theo_A3-T7term-AmpR-pBR322-LacI	4
Theo_A4	pT7-Theo_A4-T7term-AmpR-pBR322-LacI	4
Theo_A5	pT7-Theo_A5-T7term-AmpR-pBR322-LacI	4
Theo_A6	pT7-Theo_A6-T7term-AmpR-pBR322-LacI	4
Theo_A7	pT7-Theo_A7-T7term-AmpR-pBR322-LacI	4, S4, S8
Theo_A8	pT7-Theo_A8-T7term-AmpR-pBR322-LacI	4
Theo_A9	pT7-Theo_A9-T7term-AmpR-pBR322-LacI	4
Theo_A10	pT7-Theo_A10-T7term-AmpR-pBR322-LacI	4
Theo_A11	pT7-Theo_A11-T7term-AmpR-pBR322-LacI	4, S4, S8
Theo_A12	pT7-Theo_A12-T7term-AmpR-pBR322-LacI	4
Theo_A13	pT7-Theo_A13-T7term-AmpR-pBR322-LacI	4
Theo_A14	pT7-Theo_A14-T7term-AmpR-pBR322-LacI	4
Theo_A15	pT7-Theo_A15-T7term-AmpR-pBR322-LacI	4, 5, 6, 8, S6, S7, S8, S12, S19, S20
Theo_A15_pCDF	pT7-Theo_A15-T7term-SpR-CloDF13-LacI	S12
Theo_A15_pACYC	pT7-Theo_A15-T7term-ChlR-p15A-LacI	S12
Theo_A16	pT7-Theo_A16-T7term-AmpR-pBR322-LacI	S6
Theo_A17	pT7-Theo_A17-T7term-AmpR-pBR322-LacI	S6
Theo_A18	pT7-Theo_A18-T7term-AmpR-pBR322-LacI	4, 6, S6, S7, S8, S11
Theo_B1	pT7-Theo_B1-T7term-AmpR-pBR322-LacI	6, 8, 9, S11, S19
Theo_B1_pLlacO	pLlacO-Theo_B1-T7term-AmpR-pBR322-LacI	S9
Theo_B1_pJ23119	pJ23119-Theo_B1-T7term-AmpR-pBR322-LacI	S9, S13
Theo_C1	pT7-Theo_C1-T7term-AmpR-pBR322-LacI	8, S11, S19

Theo_D1	pT7-Theo_D1-T7term-AmpR-pBR322-LacI	8, S11, S19
Theo_E1	pT7-Theo_E1-T7term-AmpR-pBR322-LacI	8, S19
Theo_F1	pT7-Theo_F1-T7term-AmpR-pBR322-LacI	8, S19
Theo_G1	pT7-Theo_G1-T7term-AmpR-pBR322-LacI	8, S19
Tetra_B1	pT7-Tetra_B1-T7term-AmpR-pBR322-LacI	S14
Tetra_B2	pT7-Tetra_B2-T7term-AmpR-pBR322-LacI	S14
Tetra_B3	pT7-Tetra_B3-T7term-AmpR-pBR322-LacI	7, 8, S14, S20
Tetra_B4	pT7-Tetra_B4-T7term-AmpR-pBR322-LacI	S14
Tetra_B5	pT7-Tetra_B5-T7term-AmpR-pBR322-LacI	S14
MS2_B1	pT7-MS2_B1-T7term-AmpR-pBR322-LacI	9, S18
MS2_C1	pT7-MS2_C1-T7term-AmpR-pBR322-LacI	7, 8, S17, S18, S20
MS2_C1_pLlacO	pLlacO-MS2_C1-T7term-AmpR-pBR322-LacI	S18
MS2_C1_pJ23119	pJ23119-MS2_C1-T7term-AmpR-pBR322-LacI	S18
MS2_C2	pT7-MS2_C2-T7term-AmpR-pBR322-LacI	S17
MS2_C3	pT7-MS2_C3-T7term-AmpR-pBR322-LacI	S17
MS2_C4	pT7-MS2_C4-T7term-AmpR-pBR322-LacI	S17
MS2_C5	pT7-MS2_C5-T7term-AmpR-pBR322-LacI	S17
Theo_A_AND	pT7-Theo_A_AND-T7term-SpR-CloDF13-LacI	9, S21
MS2_A_AND	pT7-MS2_A_AND-T7term-AmpR-pBR322-LacI	9, S21
Theo_3WJ_NOT	pT7-Theo_3WJ_NOT-T7term-SpR-CloDF13-LacI	9, S21
**Other constructs**		
pECF3726_mCherry	pECF3726-mCherry-T7term-SpR-CloDF13-LacI	5
Theo_Riboswitch_ENYC3	pLlacO-Theo_Riboswitch_ENYC3-GFPmut3b-T7term-KanR-ColA-LacI	S8
T7_GFP_positive	pT7-GFPmut3b-T7term-KanR-ColA-LacI	S15
Theo_A15_cis	pT7-Theo_A15_cis-GFPmut3b-T7term-KanR-ColA-LacI	6
Theo_A18_cis	pT7-Theo_A18_cis-GFPmut3b-T7term-KanR-ColA-LacI	6
Theo_B1_cis	pT7-Theo_B1_cis-GFPmut3b-T7term-KanR-ColA-LacI	6

Table S2. Examples of construct DNA sequences

DNA sequence examples of switch & trigger constructs. Yellow – promoter. Blue – switch or trigger. Green – fluorescent protein. Pink – terminator. Gray – backbone elements.

Name (Architecture)	Sequence
**Switch**	
THS A (pT7-THS A-GFPmut3b-T7term-KanR-ColA-LacI)	TAATACGACTCACTATAGGGATTGAATATGATAGAAGTTTAGTAGTAG<br>ACAATAGAACAGAGGAGATATTGATGACTACTAAACTAAACCTGGCG<br>GCAGCGCAAAAGATGCGTAAAGGAGAAGAACTTTTCACTGGAGTTGT<br>CCCAATTCTTGTTGAATTAGATGGTGATGTTAATGGGCACAAATTTTCT<br>GTCAGTGGAGAGGGTGAAGGTGATGCAACATACGGAAAACTTACCCT<br>TAAATTTATTTGCACTACTGGAAAACTACCTGTTCCGTGGCCAACACT<br>TGTCACTACTTTCGGTTATGGTGTTCAATGCTTTGCGAGATACCCAGAT<br>CACATGAAACAGCATGACTTTTTCAAGAGTGCCATGCCCGAAGGTTAC<br>GTACAGGAAAGAACTATATTTTTCAAAGATGACGGGAACTACAAGAC<br>ACGTGCTGAAGTCAAGTTTGAAGGTGATACCCTTGTTAATAGAATCGA<br>GTTAAAAGGTATTGATTTTAAAGAAGATGGAAACATTCTTGGACACAA<br>ATTGGAATACAACTATAACTCACACAATGTATACATCATGGCAGACAA<br>ACAAAAGAATGGAATCAAAGTTAACTTCAAAATTAGACACAACATTG

	AAGATGGAAGCGTTCAACTAGCAGACCATTATCAACAAAATACTCCG<br>ATTGGCGATGGCCCTGTCCTTTTACCAGACAACCATTACCTGTCCACA<br>CAATCTGCCCTTTCGAAAGATCCCAACGAAAAGAGAGACCACATGGT<br>CCTTCTTGAGTTTGTAACCGCTGCTGGGATTACACATGGCATGGATGA<br>ACTATACAAAAGGCCTGCAGCAAACGACGAAAACTACGCTGCATCAG<br>TTTAATAAGATAAACCAGAGCGGCACGGCAAGCAGAGTATACGAGAT<br>TCGGTAGCCACCGCTGAGCAATAACTAGCATAACCCCTTGGGGCCTCT<br>AAACGGGTCTTGAGGGGTTTTTTGCTGAAACCTCAGGCATTTGAGAAG<br>CACACGGTCACACTGCTTCCGGTAGTCAATAAACCGGTAAACCAGCA<br>ATAGACATAAGCGGCTATTTAACGACCCTGCCCTGAACCGACGACAA<br>GCTGACGACCGGGTCTCCGCAAGTGGCACTTTTCGGGGAAATGTGCGC<br>GGAACCCCTATTTGTTTATTTTTCTAAATACATTCAAATATGTATCCGC<br>TCATGAATTAATTCTTAGAAAAACTCATCGAGCATCAAATGAAACTGC<br>AATTTATTCATATCAGGATTATCAATACCATATTTTTGAAAAAGCCGTT<br>TCTGTAATGAAGGAGAAAACTCACCGAGGCAGTTCCATAGGATGGCA<br>AGATCCTGGTATCGGTCTGCGATTCCGACTCGTCCAACATCAATACAA<br>CCTATTAATTTCCCCTCGTCAAAAATAAGGTTATCAAGTGAGAAATCA<br>CCATGAGTGACGACTGAATCCGGTGAGAATGGCAAAAGTTTATGCATT<br>TCTTTCCAGACTTGTTCAACAGGCCAGCCATTACGCTCGTCATCAAAA<br>TCACTCGCATCAACCAAACCGTTATTCATTCGTGATTGCGCCTGAGCG<br>AGACGAAATACGCGGTCGCTGTTAAAAGGACAATTACAAACAGGAAT<br>CGAATGCAACCGGCGCAGGAACACTGCCAGCGCATCAACAATATTTT<br>CACCTGAATCAGGATATTCTTCTAATACCTGGAATGCTGTTTTCCCGG<br>GGATCGCAGTGGTGAGTAACCATGCATCATCAGGAGTACGGATAAAA<br>TGCTTGATGGTCGGAAGAGGCATAAATTCCGTCAGCCAGTTTAGTCTG<br>ACCATCTCATCTGTAACATCATTGGCAACGCTACCTTTGCCATGTTTCA<br>GAAACAACTCTGGCGCATCGGGCTTCCCATACAATCGATAGATTGTCG<br>CACCTGATTGCCCGACATTATCGCGAGCCCATTTATACCCATATAAAT<br>CAGCATCCATGTTGGAATTTAATCGCGGCCTAGAGCAAGACGTTTCCC<br>GTTGAATATGGCTCATACTCTTCCTTTTTCAATATTATTGAAGCATTTA<br>TCAGGGTTATTGTCTCATGAGCGGATACATATTTGAATGTATTTAGAA<br>AAATAAACAAATAGGCATGCTAGCGCAGAAACGTCCTAGAAGATGCC<br>AGGAGGATACTTAGCAGAGAGACAATAAGGCCGGAGCGAAGCCGTTT<br>TTCCATAGGCTCCGCCCCCCTGACGAACATCACGAAATCTGACGCTCA<br>AATCAGTGGTGGCGAAACCCGACAGGACTATAAAGATACCAGGCGTT<br>TCCCCCTGATGGCTCCCTCTTGCGCTCTCCTGTTCCCGTCCTGCGGCGT<br>CCGTGTTGTGGTGGAGGCTTTACCCAAATCACCACGTCCCGTTCCGTG<br>TAGACAGTTCGCTCCAAGCTGGGCTGTGTGCAAGAACCCCCCGTTCAG<br>CCCGACTGCTGCGCCTTATCCGGTAACTATCATCTTGAGTCCAACCCG<br>GAAAGACACGACAAAACGCCACTGGCAGCAGCCATTGGTAACTGAGA<br>ATTAGTGGATTTAGATATCGAGAGTCTTGAAGTGGTGGCCTAACAGAG<br>GCTACACTGAAAGGACAGTATTTGGTATCTGCGCTCCACTAAAGCCAG<br>TTACCAGGTTAAGCAGTTCCCCAACTGACTTAACCTTCGATCAAACCG<br>CCTCCCCAGGCGGTTTTTTCGTTTACAGAGCAGGAGATTACGACGATC<br>GTAAAAGGATCTCAAGAAGATCCTTTACGGATTCCCGACACCATCACT<br>CTAGATTTCAGTGCAATTTATCTCTTCAAATGTAGCACCTGAAGTCAG<br>CCCCATACGATATAAGTTGTAATTCTCATGTTAGTCATGCCCCGCGCC<br>CACCGGAAGGAGCTGACTGGGTTGAAGGCTCTCAAGGGCATCGGTCG<br>AGATCCCGG`TGCCTAATGAGTGAGCTAACTTACATTAATTGCGTTGCG<br>CTCACTGCCCGCTTTCCAGTCGGGAAACCTGTCGTGCCAGCTGCATTA<br>ATGAATCGGCCAACGCGCGGGGAGAGGCGGTTTGCGTATTGGGCGCC<br>AGGGTGGTTTTTCTTTTCACCAGTGAGACGGGCAACAGCTGATTGCCC<br>TTCACCGCCTGGCCCTGAGAGAGTTGCAGCAAGCGGTCCACGCTGGTT<br>TGCCCCAGCAGGCGAAAATCCTGTTTGATGGTGGTTAACGGCGGGATA<br>TAACATGAGCTGTCTTCGGTATCGTCGTATCCCACTACCGAGATGTCC

	GCACCAACGCGCAGCCCGGACTCGGTAATGGCGCGCATTGCGCCCAG<br>CGCCATCTGATCGTTGGCAACCAGCATCGCAGTGGGAACGATGCCCTC<br>ATTCAGCATTTGCATGGTTTGTTGAAAACCGGACATGGCACTCCAGTC<br>GCCTTCCCGTTCCGCTATCGGCTGAATTTGATTGCGAGTGAGATATTTA<br>TGCCAGCCAGCCAGACGCAGACGCGCCGAGACAGAACTTAATGGGCC<br>CGCTAACAGCGCGATTTGCTGGTGACCCAATGCGACCAGATGCTCCAC<br>GCCCAGTCGCGTACCGTCTTCATGGGAGAAAATAATACTGTTGATGGG<br>TGTCTGGTCAGAGACATCAAGAAATAACGCCGGAACATTAGTGCAGG<br>CAGCTTCCACAGCAATGGCATCCTGGTCATCCAGCGGATAGTTAATGA<br>TCAGCCCACTGACGCGTTGCGCGAGAAGATTGTGCACCGCCGCTTTAC<br>AGGCTTCGACGCCGCTTCGTTCTACCATCGACACCACCACGCTGGCAC<br>CCAGTTGATCGGCGCGAGATTTAATCGCCGCGACAATTTGCGACGGCG<br>CGTGCAGGGCCAGACTGGAGGTGGCAACGCCAATCAGCAACGACTGT<br>TTGCCCGCCAGTTGTTGTGCCACGCGGTTGGGAATGTAATTCAGCTCC<br>GCCATCGCCGCTTCCACTTTTTCCCGCGTTTTCGCAGAAACGTGGCTGG<br>CCTGGTTCACCACGCGGGAAACGGTCTGATAAGAGACACCGGCATAC<br>TCTGCGACATCGTATAACGTTACTGGTTTCACATTCACCACCCTGAATT<br>GACTCTCTTCCGGGCGCTATCATGCCATACCGCGAAAGGTTTTGCGCC<br>ATTCGATGGTGTCCGGGATCTCGACGCTCTCCCTTATGAAGTCTAACG<br>CTGCTCTGGGCTAACTGTCGCGC
**Trigger**	
Theo_A2 (pT7-Theo_A2-T7term-AmpR-pBR322-LacI)	TAATACGACTCACTATAGGGATACACATAGAATCATGTGTATAACACT<br>ACTAAACTTCAACTATGATACCAGCATCGTCTTGATGCCCTTGGCAGC<br>ATAAACTATCATATTCAATCACTAGCATAACCCCTTGGGGCCTCTAAA<br>CGGGTCTTGAGGGGTTTTTTGCTGAAAGGAGGAACTATATCCGGATAT<br>CCCGCAAGAGGCCCGGCAGTACCGGCATAACCAAGCCTATGCCTACA<br>GCATCCAGGGTGACGGTGCCGAGGATGACGATGAGCGCATTGTTAGA<br>TTTCATACACGGTGCCTGACTGCGTTAGCAATTTAACTGTGATAAACT<br>ACCGCATTAAAGCTTATCGATGATAAGCTGTCAAACATGAGAATTCTT<br>GAAGACGAAAGGGCCTCGTGATACGCCTATTTTTATAGGTTAATGTCA<br>TGATAATAATGGTTTCTTAGACGTCAGGTGGCACTTTTCGGGGAAATG<br>TGCGCGGAACCCCTATTTGTTTATTTTTCTAAATACATTCAAATATGTA<br>TCCGCTCATGAGACAATAACCCTGATAAATGCTTCAATAATATTGAAA<br>AAGGAAGAGTATGAGTATTCAACATTTCCGTGTCGCCCTTATTCCCTTT<br>TTTGCGGCATTTTGCCTTCCTGTTTTTGCTCACCCAGAAACGCTGGTGA<br>AAGTAAAAGATGCTGAAGATCAGTTGGGTGCACGAGTGGGTTACATC<br>GAACTGGATCTCAACAGCGGTAAGATCCTTGAGAGTTTTCGCCCCGAA<br>GAACGTTTTCCAATGATGAGCACTTTTAAAGTTCTGCTATGTGGCGCG<br>GTATTATCCCGTGTTGACGCCGGGCAAGAGCAACTCGGTCGCCGCATA<br>CACTATTCTCAGAATGACTTGGTTGAGTACTCACCAGTCACAGAAAAG<br>CATCTTACGGATGGCATGACAGTAAGAGAATTATGCAGTGCTGCCATA<br>ACCATGAGTGATAACACTGCGGCCAACTTACTTCTGACAACGATCGGA<br>GGACCGAAGGAGCTAACCGCTTTTTTGCACAACATGGGGGATCATGTA<br>ACTCGCCTTGATCGTTGGGAACCGGAGCTGAATGAAGCCATACCAAA<br>CGACGAGCGTGACACCACGATGCCTGCAGCAATGGCAACAACGTTGC<br>GCAAACTATTAACTGGCGAACTACTTACTCTAGCTTCCCGGCAACAAT<br>TAATAGACTGGATGGAGGCGGATAAAGTTGCAGGACCACTTCTGCGC<br>TCGGCCCTTCCGGCTGGCTGGTTTATTGCTGATAAATCTGGAGCCGGT<br>GAGCGTGGGTCTCGCGGTATCATTGCAGCACTGGGGCCAGATGGTAA<br>GCCCTCCCGTATCGTAGTTATCTACACGACGGGGAGTCAGGCAACTAT<br>GGATGAACGAAATAGACAGATCGCTGAGATAGGTGCCTCACTGATTA<br>AGCATTGGTAACTGTCAGACCAAGTTTACTCATATATACTTTAGATTG<br>ATTTAAAACTTCATTTTTAATTTAAAAGGATCTAGGTGAAGATCCTTTT<br>TGATAATCTCATGACCAAAATCCCTTAACGTGAGTTTCGTTCCACTG<br>AGCGTCAGACCCCGTAGAAAAGATCAAAGGATCTTCTTGAGATCCTTT

	TTTTCTGCGCGTAATCTGCTGCTTGCAAACAAAAAAACCACCGCTACC<br>AGCGGTGGTTTGTTTGCCGGATCAAGAGCTACCAACTCTTTTTCCGAA<br>GGTAACTGGCTTCAGCAGAGCGCAGATACCAAATACTGTCCTTCTAGT<br>GTAGCCGTAGTTAGGCCACCACTTCAAGAACTCTGTAGCACCGCCTAC<br>ATACCTCGCTCTGCTAATCCTGTTACCAGTGGCTGCTGCCAGTGGCGA<br>TAAGTCGTGTCTTACCGGGTTGGACTCAAGACGATAGTTACCGGATAA<br>GGCGCAGCGGTCGGGCTGAACGGGGGGTTCGTGCACACAGCCCAGCT<br>TGGAGCGAACGACCTACACCGAACTGAGATACCTACAGCGTGAGCTA<br>TGAGAAAGCGCCACGCTTCCCGAAGGGAGAAAGGCGGACAGGTATCC<br>GGTAAGCGGCAGGGTCGGAACAGGAGAGCGCACGAGGGAGCTTCCA<br>GGGGGAAACGCCTGGTATCTTTATAGTCCTGTCGGGTTTCGCCACCTC<br>TGACTTGAGCGTCGATTTTTGTGATGCTCGTCAGGGGGGCGGAGCCTA<br>TGGAAAAACGCCAGCAACGCGGCCTTTTTACGGTTCCTGGCCTTTTGC<br>TGGCCTTTTGCTCACATGTTCTTTCCTGCGTTATCCCCTGATTCTGTGG<br>ATAACCGTATTACCGCCTTTGAGTGAGCTGATACCGCTCGCCGCAGCC<br>GAACGACCGAGCGCAGCGAGTCAGTGAGCGAGGAAGCGGAAGAGCG<br>CCTGATGCGGTATTTTCTCCTTACGCATCTGTGCGGTATTTCACACCGC<br>ATATATGGTGCACTCTCAGTACAATCTGCTCTGATGCCGCATAGTTAA<br>GCCAGTATACACTCCGCTATCGCTACGTGACTGGGTCATGGCTGCGCC<br>CCGACACCCGCCAACACCCGCTGACGCGCCCTGACGGGCTTGTCTGCT<br>CCCGGCATCCGCTTACAGACAAGCTGTGACCGTCTCCGGGAGCTGCAT<br>GTGTCAGAGGTTTTCACCGTCATCACCGAAACGCGCGAGGCAGCTGCG<br>GTAAAGCTCATCAGCGTGGTCGTGAAGCGATTCACAGATGTCTGCCTG<br>TTCATCCGCGTCCAGCTCGTTGAGTTTCTCCAGAAGCGTTAATGTCTGG<br>CTTCTGATAAAGCGGGCCATGTTAAGGGCGGTTTTTTCCTGTTTGGTCA<br>CTGATGCCTCCGTGTAAGGGGGATTTCTGTTCATGGGGGTAATGATAC<br>CGATGAAACGAGAGAGGATGCTCACGATACGGGTTACTGATGATGAA<br>CATGCCCGGTTACTGGAACGTTGTGAGGGTAAACAACTGGCGGTATG<br>GATGCGGCGGGACCAGAGAAAAATCACTCAGGGTCAATGCCAGCGCT<br>TCGTTAATACAGATGTAGGTGTTCCACAGGGTAGCCAGCAGCATCCTG<br>CGATGCAGATCCGGAACATAATGGTGCAGGGCGCTGACTTCCGCGTTT<br>CCAGACTTTACGAAACACGGAAACCGAAGACCATTCATGTTGTTGCTC<br>AGGTCGCAGACGTTTTGCAGCAGCAGTCGCTTCACGTTCGCTCGCGTA<br>TCGGTGATTCATTCTGCTAACCAGTAAGGCAACCCCGCCAGCCTAGCC<br>GGGTCCTCAACGACAGGAGCACGATCATGCGCACCCGTGGCCAGGAC<br>CCAACGCTGCCCGAGATGCGCCGCGTGCGGCTGCTGGAGATGGCGGA<br>CGCGATGGATATGTTCTGCCAAGGGTTGGTTTGCGCATTCACAGTTCT<br>CCGCAAGAATTGATTGGCTCCAATTCTTGGAGTGGTGAATCCGTTAGC<br>GAGGTGCCGCCGGCTTCCATTCAGGTCGAGGTGGCCCGGCTCCATGCA<br>CCGCGACGCAACGCGGGGAGGCAGACAAGGTATAGGGCGGCGCCTAC<br>AATCCATGCCAACCCGTTCCATGTGCTCGCCGAGGCGGCATAAATCGC<br>CGTGACGATCAGCGGTCCAGTGATCGAAGTTAGGCTGGTAAGAGCCG<br>CGAGCGATCCTTGAAGCTGTCCCTGATGGTCGTCATCTACCTGCCTGG<br>ACAGCATGGCCTGCAACGCGGGCATCCCGATGCCGCCGGAAGCGAGA<br>AGAATCATAATGGGGAAGGCCATCCAGCCTCGCGTCGCGAACGCCAG<br>CAAGACGTAGCCCAGCGCGTCGGCCGCCATGCCGGCGATAATGGCCT<br>GCTTCTCGCCGAAACGTTTGGTGGCGGGACCAGTGACGAAGGCTTGA<br>GCGAGGGCGTGCAAGATTCCGAATACCGCAAGCGACAGGCCGATCAT<br>CGTCGCGCTCCAGCGAAAGCGGTCCTCGCCGAAAATGACCCAGAGCG<br>CTGCCGGCACCTGTCCTACGAGTTGCATGATAAAGAAGACAGTCATAA<br>GTGCGGCGACGATAGTCATGCCCCGCGCCCACCGGAAGGAGCTGACT<br>GGGTTGAAGGCTCTCAAGGGCATCGGTCGAGATCCCGGTGCCTAATG<br>AGTGAGCTAACTTACATTAATTGCGTTGCGCTCACTGCCCGCTTTCCA<br>GTCGGGAAACCTGTCGTGCCAGCTGCATTAATGAATCGGCCAACGCGC<br>GGGGAGAGGCGGTTTGCGTATTGGGCGCCAGGGTGGTTTTTCTTTTCA

	CCAGTGAGACGGGCAACAGCTGATTGCCCTTCACCGCCTGGCCCTGAGAGAGTTGCAGCAAGCGGTCCACGCTGGTTTGCCCCAGCAGGCGAAAATCCTGTTTGATGGTGGTTAACGGCGGGATATAACATGAGCTGTCTTCGGTATCGTCGTATCCCACTACCGAGATATCCGCACCAACGCGCAGCCCGGACTCGGTAATGGCGCGCATTGCGCCCAGCGCCATCTGATCGTTGGCAACCAGCATCGCAGTGGGAACGATGCCCTCATTCAGCATTTGCATGGTTTGTTGAAAACCGGACATGGCACTCCAGTCGCCTTCCCGTTCCGCTATCGGCTGAATTTGATTGCGAGTGAGATATTTATGCCAGCCAGCCAGACGCAGACGCGCCGAGACAGAACTTAATGGGCCCGCTAACAGCGCGATTTGCTGGTGACCCAATGCGACCAGATGCTCCACGCCCAGTCGCGTACCGTCTTCATGGGAGAAAATAATACTGTTGATGGGTGTCTGGTCAGAGACATCAAGAAATAACGCCGGAACATTAGTGCAGGCAGCTTCCACAGCAATGGCATCCTGGTCATCCAGCGGATAGTTAATGATCAGCCCACTGACGCGTTGCGCGAGAAGATTGTGCACCGCCGCTTTACAGGCTTCGACGCCGCTTCGTTCTACCATCGACACCACCACGCTGGCACCCAGTTGATCGGCGCGAGATTTAATCGCCGCGACAATTTGCGACGGCGCGTGCAGGGCCAGACTGGAGGTGGCAACGCCAATCAGCAACGACTGTTTGCCCGCCAGTTGTTGTGCCACGCGGTTGGGAATGTAATTCAGCTCCGCCATCGCCGCTTCCACTTTTTCCCGCGTTTTCGCAGAAACGTGGCTGGCCTGGTTCACCACGCGGGAAACGGTCTGATAAGAGACACCGGCATACTCTGCGACATCGTATAACGTTACTGGTTTCACATTCACCACCCTGAATTGACTCTCTTCCGGGCGCTATCATGCCATACCGCGAAAGGTTTTGCGCCATTCGATGGTGTCCGGGATCTCGACGCTCTCCCTTATGCGACTCCTGCATTAGGAAGCAGCCCAGTAGTAGGTTGAGGCCGTTGAGCACCGCCGCCGCAAGGAATGGTGCATGCAAGGAGATGGCGCCCAACAGTCCCCCGGCCACGGGGCCTGCCACCATACCCACGCCGAAACAAGCGCTCATGAGCCCGAAGTGGCGAGCCCGATCTTCCCCATCGGTGATGTCGGCGATATAGGCGCCAGCAACCGCACCTGTGGCGCCGGTGATGCCGGCCACGATGCGTCCGGCGTAGAGGATCGAGATCTCGAACGTGTACGGGCTATCTGGCTTTCGTTGCGC

Table S3. Module sequences

DNA sequences of each module used in this study. Sequences can be constructed by combining the following modules according to the architectures in Table S1.

Name	Sequence
**Switch & trigger**	
**Switch**	
THS A	ATTGAATATGATAGAAGTTTAGTAGTAGACAATAGAACAGAGGAGATATTGATGACTACTAAACTAAACCTGGCGGCAGCGCAAAAG
THS A_ B0030	ATTGAATATGATAGAAGTTTAGTAGTAGACAATAGAACAAAGAGGGGACAATATTGATGACTACTAAACTAAACCTGGCGGCAGCGCAAAAG
THS A_ B0033	ATTGAATATGATAGAAGTTTAGTAGTAGACAATAGAACTCACACAGGACTATTGATGACTACTAAACTAAACCTGGCGGCAGCGCAAAAG
THS A_ B0034	ATTGAATATGATAGAAGTTTAGTAGTAGACAATAGAACAAAGAGGAGAAATATTGATGACTACTAAACTAAACCTGGCGGCAGCGCAAAAG
THS A_ J61100	ATTGAATATGATAGAAGTTTAGTAGTAGACAATAGAACGAAAGAGGGGACAATATTGATGACTACTAAACTAAACCTGGCGGCAGCGCAAAAG
THS A_ J61125	ATTGAATATGATAGAAGTTTAGTAGTAGACAATAGAACGAAAGAGCCGAGTTTATTGATGACTACTAAACTAAACCTGGCGGCAGCGCAAAAG
THS A_ J61132	ATTGAATATGATAGAAGTTTAGTAGTAGACAATAGAACGAAAGACAGGATTATATTGATGACTACTAAACTAAACCTGGCGGCAGCGCAAAAG
THS B	CTTATGAGTGTAATACGTTCTATGTCAGATTCAAGAACAGAGGAGATTGAAATGGACATAGAACGAAACCTGGCGGCAGCGCAAAAG

THS C	AGTAAGATAATGAAGGTAGGTATGTTAAACTTTAGAACAGAGGAGATAA AGATGAACATACCTACGAACCTGGCGGCAGCGCAAAAG
THS D	ACTGATTTGAATACACTGCTTCGTTCAAGATTCAGAACAGAGGAGATGAA TATGGAACGAAGCAGAAACCTGGCGGCAGCGCAAAAG
THS E	CTTGAATTTGAATTACGTCGTCTTATAGAATTCAGAACAGAGGAGATGAA TATGATAAGACGACGAAACCTGGCGGCAGCGCAAAAG
THS F	ACTACTATTGATTACACGCTTTACTTCGAAATTCATAATGAACAGAGGAG ATATGAAATGCGAAGTAAAGCGAACCTGGCGGCAGCGCAAAAG
THS G	ATCTATGTTCAATTCTATTCGTCGTCTCATGAGTATAGTAAACAGAGGAG ATATACTATGGAGACGACGAATAACCTGGCGGCAGCGCAAAAG
3WJ A	ACTAATCAGATCTACTTGTTATAGTTATGAACAGAGGAGACATAACATGA ACAAGCACCTAACAAGACTAATCAACCTGGCGGCAGCGCAAAAG
**THS trigger**	
THS Trigger A	ATACACATAGAATCATGTGTATAACACTACTAAACTTCTATCATATTCAA TCAC
THS Trigger B	AGTCTTCAAGATAATGAAGACTCTGGACATAGAACGTATTACACTCATAA GATA
THS Trigger C	CTCGATCACTAATCTGATCGAGACGAACATACCTACCTTCATTATCTTACT TGT
ScD	TCTCACGCCCTCAGCTGGGCGTGAGATGAGCCTCGTCTCCAGATGACGAG GCAACGTAGGATCTGACTGATCCTACTAT
Nonsense	ATACACATAGAATCATGTGTATAACTACTAAACTTCTACATTCTCGCAAA CTCCTACAAATCATCACACTTCCACCTCATTATCATATTCAC
**Apta-trigger**	
Theo_A1	ATACACATAGAATCATGTGTATAACACTACTAAACTTCAACGGTGATACC AGCATCGTCTTGATGCCCTTGGCAGCACCAACTATCATATTCAATCAC
Theo_A2	ATACACATAGAATCATGTGTATAACACTACTAAACTTCAACTATGATACC AGCATCGTCTTGATGCCCTTGGCAGCATAAACTATCATATTCAATCAC
Theo_A2_shift1	ATACACATAGAATCATGTGTATAACACTACTAAACAACTATGATACCAGC ATCGTCTTGATGCCCTTGGCAGCATAAACTTCTATCATATTCAATCAC
Theo_A2_shift2	ATACACATAGAATCATGTGTATAACACTACTAAACTAACTATGATACCAG CATCGTCTTGATGCCCTTGGCAGCATAAACTCTATCATATTCAATCAC
Theo_A2_shift3	ATACACATAGAATCATGTGTATAACACTACTAAACTTAACTATGATACCA GCATCGTCTTGATGCCCTTGGCAGCATAAACCTATCATATTCAATCAC
Theo_A2_shift4	ATACACATAGAATCATGTGTATAACACTACTAAACTTCTAACTATGATAC CAGCATCGTCTTGATGCCCTTGGCAGCATAAACATCATATTCAATCAC
Theo_A2_shift5	ATACACATAGAATCATGTGTATAACACTACTAAACTTCTAAACTATGATA CCAGCATCGTCTTGATGCCCTTGGCAGCATAAACTCATATTCAATCAC
Theo_A2_shift6	ATACACATAGAATCATGTGTATAACACTACTAAACTTCTATAACTATGAT ACCAGCATCGTCTTGATGCCCTTGGCAGCATAAACCATATTCAATCAC
Theo_A2_trim1	ATACACATAGAATCATGTGTATAACACTACTAAACTTCAACTATGATACC AGCATCGTCTTGATGCCCTTGGCAGCATAAACTATCATATTCACAC
Theo_A2_trim2	ATACACATAGAATCATGTGTATAACACTACTAAACTTCAACTATGATACC AGCATCGTCTTGATGCCCTTGGCAGCATAAACTATCATATTCAC
Theo_A2_trim3	ATACACATAGAATCATGTGTATAACTACTAAACTTCAACTATGATACCAG CATCGTCTTGATGCCCTTGGCAGCATAAACTATCATATTCAATCAC
Theo_A2_trim4	ATACACATAGAATCATGTGTATAACCTAAACTTCAACTATGATACCAGCA TCGTCTTGATGCCCTTGGCAGCATAAACTATCATATTCAATCAC
Theo_A3	ATACACATAGAATCATGTGTATAACACTACTAAACTTCAACGCGCATACC AGCATCGTCTTGATGCCCTTGGCAGGCGCAACTATCATATTCAATCAC
Theo_A4	ATACACATAGAATCATGTGTATAACACTACTAAACTTCAACACGCATACC AGCATCGTCTTGATGCCCTTGGCAGGCGTAACTATCATATTCAATCAC
Theo_A5	ATACACATAGAATCATGTGTATAACACTACTAAACTTCAACCCTAATACC AGCATCGTCTTGATGCCCTTGGCAGTAGGAACTATCATATTCAATCAC

Theo_A6	ATACACATAGAATCATGTGTATAACACTACTAAACTTCAACGCAATACCAGCATCGTCTTGATGCCCTTGGCAGTGCAACTATCATATTCAATCAC
Theo_A7	ATACACATAGAATCATGTGTATAACACTACTAAACTTCAACAGCATACCAGCATCGTCTTGATGCCCTTGGCAGGCTAACTATCATATTCAATCAC
Theo_A8	ATACACATAGAATCATGTGTATAACACTACTAAACTTCAACTATTTTATACCAGCATCGTCTTGATGCCCTTGGCAGAAAATAAACTATCATATTCAATCAC
Theo_A9	ATACACATAGAATCATGTGTATAACACTACTAAACTTCAACTATTTATACCAGCATCGTCTTGATGCCCTTGGCAGAAATAAACTATCATATTCAATCAC
Theo_A10	ATACACATAGAATCATGTGTATAACACTACTAAACTTCAACCGATACCAGCATCGTCTTGATGCCCTTGGCAGCGAACTATCATATTCAATCAC
Theo_A11	ATACACATAGAATCATGTGTATAACACTACTAAACTTCAACTATTATACCAGCATCGTCTTGATGCCCTTGGCAGAATAAACTATCATATTCAATCAC
Theo_A12	ATACACATAGAATCATGTGTATAACACTACTAAACTTCAACTTAATACCAGCATCGTCTTGATGCCCTTGGCAGTAAAACTATCATATTCAATCAC
Theo_A13	ATACACATAGAATCATGTGTATAACACTACTAAACTTCAACTAATACCAGCATCGTCTTGATGCCCTTGGCAGTAAACTATCATATTCAATCAC
Theo_A14	ATACACATAGAATCATGTGTATAACACTACTAAACTTCAACATATACCAGCATCGTCTTGATGCCCTTGGCAGATAACTATCATATTCAATCAC
Theo_A15	ATACACATAGAATCATGTGTATAACTACTAAACTTCAACTATGATACCAGCATCGTCTTGATGCCCTTGGCAGCATAAACTATCATATTCAC
Theo_A16	ATACACATAGAATCATGTGTATAACACTACTAAACTTCAACTATTATACCAGCATCGTCTTGATGCCCTTGGCAGAATAAACTATCATATTCAC
Theo_A17	ATACACATAGAATCATGTGTATAACTACTAAACTTCAACTATTATACCAGCATCGTCTTGATGCCCTTGGCAGAATAAACTATCATATTCAATCAC
Theo_A18	ATACACATAGAATCATGTGTATAACTACTAAACTTCAACTATTATACCAGCATCGTCTTGATGCCCTTGGCAGAATAAACTATCATATTCAC
Theo_B1	AGTCTTCAAGATAATGAAGACTCTGGACATAGAACGTACTCAAATATACCAGCATCGTCTTGATGCCCTTGGCAGATTTCCTTTACACTCATAAGATA
Theo_C1	CTCGATCACTAATCTGATCGAGACGAACATACCTACCTCTTAATTATACCAGCATCGTCTTGATGCCCTTGGCAGAATTTCCTCATTATCTTACTTGT
Theo_D1	AGTTGCGGCACGGACCGCAACTATAGAACGAAGCAGTGACGTATAATACCAGCATCGTCTTGATGCCCTTGGCAGTATACAATATTCAAATCAGTTAG
Theo_E1	ACGGTCCGGAGTGGCGGACCGTAAGATAAGACGACGTAAACTATGATACCAGCATCGTCTTGATGCCCTTGGCAGCATAAACATTCAAATTCAAGGTA
Theo_F1	CGTGAGATAAGCACATCTCACGAGACGAAGTAAAGCGTGAACTATTATACCAGCATCGTCTTGATGCCCTTGGCAGAATAAACTAATCAATAGTAGTAAG
Theo_G1	ACCGGTCTTTGCAGAGACCGGTGTAGAGACGACGAATAGAACTATGATACCAGCATCGTCTTGATGCCCTTGGCAGCATAAACAATTGAACATAGATAAG
Tetra_B1	AGTCTTCAAGATAATGAAGACTCTGGACATAGAACGTATAAGGCCTAAAACATACCAGATTTCGATCTGGAGAGGTGAAGAATACGACCACCTAGGCCAAATTACACTCATAAGATA
Tetra_B2	AGTCTTCAAGATAATGAAGACTCTGGACATAGAACGTATAAGGCAAAACATACCAGATTTCGATCTGGAGAGGTGAAGAATACGACCACCTGCCAAATTACACTCATAAGATA
Tetra_B3	AGTCTTCAAGATAATGAAGACTCTGGACATAGAACGTATAATCCAAAACATACCAGATTTCGATCTGGAGAGGTGAAGAATACGACCACCTGGAAAATTACACTCATAAGATA
Tetra_B4	AGTCTTCAAGATAATGAAGACTCTGGACATAGAACGTATAACATTAAAACATACCAGATTTCGATCTGGAGAGGTGAAGAATACGACCACCTAATGAAATTACACTCATAAGATA

Tetra_B5	AGTCTTCAAGATAATGAAGACTCTGGACATAGAACGTATAACTAAAACA TACCAGATTTCGATCTGGAGAGGTGAAGAATACGACCACCTAGAAATTA CACTCATAAGATA
MS2_B1	CTCGCCCGCCAAAGGGCGAGCAAAGACATAGAACGTACCAAACATGAGG ACCACCCATGTTAATTTACACTCATAAGCCC
MS2_C1	CTCGATCACTAATCTGATCGAGACGAACATACCTACCTACAAACATGAGG ACCACCCATGTTAACTCATTATCTTACTTGT
MS2_C2	CTCGATCACTAATCTGATCGAGACGAACATACCTACCTACAACATGAGGA CCACCCATGTAACTCATTATCTTACTTGT
MS2_C3	CTCGATCACTAATCTGATCGAGACGAACATACCTACCTACAAACATTAGG ACCACCAATGTTAACTCATTATCTTACTTGT
MS2_C4	CTCGATCACTAATCTGATCGAGACGAACATACCTACCTACAAACATAGGA CCACCATGTTAACTCATTATCTTACTTGT
MS2_C5	CTCGATCACTAATCTGATCGAGACGAACATACCTACCTACAAATGAGGAC CACCCATTAACTCATTATCTTACTTGT
Theo_A_AND	ATACACATAGAATCATGTGTATAATACTACTAAACTTCCCATATGATACC AGCATCGTCTTGATGCCCTTGGCAGCATATAGAGCAGAATTGAAATGTGA ACCC
MS2_A_AND	CTCGATCACTAATCTGATCGAGATATTCACATTTCAATTCTGCTCTAAAAC ATGAGGACCACCCATGTTACTTATCATATTCAATTTT
Theo_3WJ_NOT	ACCTAACATAAACTTGTTAGGTGCAAAATATACCAGCATCGTCTTGATGC CCTTGGCAGATTTCGTAGATCTGATTAGTGTGACC
**Other constructs**	
Theo_Riboswitch_ENYC3	GGTGATACCAGCATCGTCTTGATGCCCTTGGCAGCACCCTGCTAAGGAGG TAACAACAAG
Theo_A15_cis	ATACACATAGAATCATGTGTATAACTACTAAACTTCAACTATGATACCAG CATCGTCTTGATGCCCTTGGCAGCATAAACTATCATATTCACATAAGATT GAATATGATAGAAGTTTAGTAGTAGACAATAGAACAGAGGAGATATTGA TGACTACTAAACTAAACCTGGCGGCAGCGCAAAAG
Theo_A18_cis	ATACACATAGAATCATGTGTATAACTACTAAACTTCAACTATTATACCAG CATCGTCTTGATGCCCTTGGCAGAATAAACTATCATATTCACATAAGATT GAATATGATAGAAGTTTAGTAGTAGACAATAGAACAGAGGAGATATTGA TGACTACTAAACTAAACCTGGCGGCAGCGCAAAAG
Theo_B1_cis	AGTCTTCAAGATAATGAAGACTCTGGACATAGAACGTACTCAAATATACC AGCATCGTCTTGATGCCCTTGGCAGATTTCCTTTACACTCATAAGATAGA ACGCTTATGAGTGTAATACGTTCTATGTCAGATTCAAGAACAGAGGAGAT TGAAATGGACATAGAACGAAACCTGGCGGCAGCGCAAAAG
**Promoter**	
pT7	TAATACGACTCACTATAGGG
pLlacO	ATAAATGTGAGCGGATAACATTGACATTGTGAGCGGATAACAAGATACT GAGCAC
pJ23119	TTGACAGCTAGCTCAGTCCTAGGTATAATGCTAGC
pECF3726	GCCTCCACACCGCTCGTCACATCCTGTGATCCACTCTTCATCCCGCTACGT AACACCTCTGCATCGCGAACCAAAACCAG
**Protein**	
GFPmut3b	ATGCGTAAAGGAGAAGAACTTTTCACTGGAGTTGTCCCAATTCTTGTTGA ATTAGATGGTGATGTTAATGGGCACAAATTTTCTGTCAGTGGAGAGGGTG AAGGTGATGCAACATACGGAAAACTTACCCTTAAATTTATTTGCACTACT GGAAAACTACCTGTTCCGTGGCCAACACTTGTCACTACTTTCGGTTATGG TGTTCAATGCTTTGCGAGATACCCAGATCACATGAAACAGCATGACTTTT TCAAGAGTGCCATGCCCGAAGGTTACGTACAGGAAAGAACTATATTTTTC AAAGATGACGGGAACTACAAGACACGTGCTGAAGTCAAGTTTGAAGGTG ATACCCTTGTTAATAGAATCGAGTTAAAAGGTATTGATTTTAAAGAAGAT

	GGAAACATTCTTGGACACAAATTGGAATACAACTATAACTCACACAATGT<br>ATACATCATGGCAGACAAACAAAAGAATGGAATCAAAGTTAACTTCAAA<br>ATTAGACACAACATTGAAGATGGAAGCGTTCAACTAGCAGACCATTATC<br>AACAAAATACTCCGATTGGCGATGGCCCTGTCCTTTTACCAGACAACCAT<br>TACCTGTCCACACAATCTGCCCTTTCGAAAGATCCCAACGAAAAGAGAG<br>ACCACATGGTCCTTCTTGAGTTTGTAACCGCTGCTGGGATTACACATGGC<br>ATGGATGAACTATACAAAAGGCCTGCAGCAAACGACGAAAACTACGCTG<br>CATCAGTTTA
mCherry	ATGCGTAAAGTGAGCAAGGGCGAAGAAGATAACATGGCCATCATCAAGG<br>AGTTCATGCGCTTCAAGGTTCACATGGAGGGCTCCGTGAACGGCCACGA<br>GTTCGAGATCGAGGGCGAGGGCGAGGGCCGCCCCTACGAGGGCACCCAG<br>ACCGCCAAGCTGAAGGTGACCAAGGGTGGCCCCCTGCCCTTCGCCTGGG<br>ACATCCTGTCCCCTCAGTTCATGTACGGCTCCAAGGCCTACGTGAAGCAC<br>CCCGCCGACATCCCCGACTACTTGAAGCTGTCCTTCCCCGAGGGCTTCAA<br>GTGGGAGCGCGTGATGAACTTCGAGGACGGCGGCGTGGTGACCGTGACC<br>CAGGACTCCTCCCTGCAAGACGGCGAGTTCATCTACAAGGTGAAGCTGC<br>GCGGCACCAACTTCCCCTCCGACGGCCCCGTAATGCAGAAGAAGACTAT<br>GGGCTGGGAGGCCTCCTCCGAGCGGATGTACCCCGAGGACGGCGCGCTG<br>AAGGGCGAGATCAAGCAGAGGCTGAAGCTGAAGGACGGCGGCCACTAC<br>GACGCTGAGGTCAAGACCACCTACAAGGCCAAGAAGCCCGTGCAACTGC<br>CCGGCGCGTACAACGTCAACATCAAGTTGGACATCACCTCCCACAACGA<br>GGACTACACCATCGTGGAACAGTACGAACGCGCCGAGGGCCGCCACTCC<br>ACCGGCGGCATGGACGAGCTGTACAAGTAA
YFP	AGCAAGGGCGAGGAGCTGTTCACCGGGGTGGTGCCCATCCTGGTCGAGC<br>TGGACGGCGACGTAAACGGCCACAAGTTCAGCGTGTCCGGCGAGGGCGA<br>GGGCGATGCCACCTACGGCAAGCTGACCCTGAAGTTCATCTGCACCACC<br>GGCAAGCTGCCCGTGCCCTGGCCCACCCTCGTGACCACCTTCGGCTACGG<br>CCTGAAGTGCTTCGCCCGCTACCCCGACCACATGAAGCAGCACGACTTCT<br>TCAAGTCCGCCATGCCCGAAGGCTACGTCCAGGAGCGCACCATCTTCTTC<br>AAGGACGACGGCAACTACAAGACCCGCGCCGAGGTGAAGTTCGAGGGC<br>GACACCCTGGTGAACCGCATCGAGCTGAAGGGCATCGACTTCAAGGAGG<br>ACGGCAACATCCTGGGGCACAAGCTGGAGTACAACTACAACAGCCACAA<br>CGTCTATATCATGGCCGACAAGCAGAAGAACGGCATCAAGGTGAACTTC<br>AAGATCCGCCACAACATCGAGGACGGCAGCGTGCAGCTCGCCGACCACT<br>ACCAGCAGAACACCCCCATCGGCGACGGCCCCGTGCTGCTGCCCGACAA<br>CCACTACCTGAGCTACCAGTCCGCCCTGAGCAAAGACCCCAACGAGAAG<br>CGCGATCACATGGTCCTGCTGGAGTTCGTGACCGCCGCCGGGATCACTCT<br>CGGCATGGACGAGCTGTACAAGTAA
Cerulean	ATGGTGAGCAAGGGCGAGGAGCTGTTCACCGGGGTGGTGCCCATCCTGG<br>TCGAGCTGGACGGCGACGTTAACGGTCATAAATTCAGCGTTAGCGGAGA<br>AGGCGAAGGCGATGCGACTTACGGCAAACTGACTTTGAAATTTATCTGTA<br>CCACGGGTAAGCTTCCTGTGCCATGGCCAACGTTGGTCACCACCCTGACG<br>TGGGGTGTGCAGTGCTTTGCTCGCTATCCCGACCACATGAAACAACATGA<br>CTTTTTCAAATCCGCGATGCCTGAGGGCTATGTCCAGGAGCGTACGATCT<br>TTTTTAAAGATGATGGCAACTACAAAACGCGCGCGGAAGTGAAATTCGA<br>AGGCGATACCCTGGTAAACCGCATCGAACTGAAAGGGATCGACTTCAAA<br>GAGGATGGGAACATTCTCGGCCATAAACTGGAATATAATGCGATTAGCG<br>ACAATGTTTATATTACTGCTGATAAGCAGAAAAATGGTATCAAAGCTAAC<br>TTTAAAATTCGCCACAATATTGAAGATGGCTCCGTGCAGTTAGCAGATCA<br>TTACCAACAAAACACCCCAATTGGGGACGGCCCGGTGCTTCTGCCGGAT<br>AATCATTATCTGTCTACGCAATCCAAACTGAGTAAAGATCCAAATGAAAA<br>GCGCGATCATATGGTCCTGCTGGAGTTCGTGACCGCCGCCGGGATCACTC<br>TCGGCATGGACGAGCTGTACAAGTAATAA
BFP	ATGAGCGAGCTGATTAAGGAGAACATGCACATGAAGCTGTACATGGAGG<br>GCACCGTGGACAACCATCACTTCAAGTGCACATCCGAGGGCGAAGGCAA

	GCCCTACGAGGGCACCCAGACCATGAGAATCAAGGTGGTCGAGGGCGGC<br>CCTCTCCCCTTCGCCTTCGACATCCTGGCTACTAGCTTCCTCTACGGCAGC<br>AAGACCTTCATCAACCACACCCAGGGCATCCCCGACTTCTTCAAGCAGTC<br>CTTCCCTGAGGGCTTCACATGGGAGAGAGTCACCACATACGAAGACGGG<br>GGCGTGCTGACCGCTACCCAGGACACCAGCCTCCAGGACGGCTGCCTCA<br>TCTACAACGTCAAGATCAGAGGGGTGAACTTCACATCCAACGGCCCTGTG<br>ATGCAGAAGAAAACACTCGGCTGGGAGGCCTTCACCGAGACGCTGTACC<br>CCGCTGACGGCGGCCTGGAAGGCAGAAACGACATGGCCCTGAAGCTCGT<br>GGGCGGGAGCCATCTGATCGCAAACGCCAAGACCACATATAGATCCAAG<br>AAACCCGCTAAGAACCTCAAGATGCCTGGCGTCTACTATGTGGACTACAG<br>ACTGGAAAGAATCAAGGAGGCCAACAACGAGACCTACGTCGAGCAGCA<br>CGAGGTGGCAGTGGCCAGATACTGCGACCTCCCTAGCAAACTGGGGCAC<br>AAGCTTAATGCAGCAAACGACGAAAACTACGCTGCATCAGTTTAA
ECF987	ATGGGCAGCAGCCATCATCATCATCATCACAGCAGCGGCCTGGAAGTTCT<br>GTTCCAGGGGCCCCATATGATGAGCGATAGTCCGCAGAAACTGGGTCGT<br>AATGAATGGAATGCCTATATGGATAAAGTGAAAGCCAAAGATCGTGAAG<br>CCTTTGCATTTGTGTTTCGTTTTTATGCACCGAAACTGAAACAGTTCGCCT<br>ATAAACATGTGGGTAATGAACAGGTTGCCATGGAAATGGTTCAAGAAAC<br>CATGGCCACCGTTTGGCAGAAAGCACATCTGTATGATGGTAAAAAAAGC<br>GCACTGAGCACCTGGATTTATACCATTATTCGCAACCTGTGCTTTGATCTG<br>CTGCGTAAACAGAAAGGTAAAGAACTGCATATCCACTCCGATGATATTTG<br>GCCGAGCGAATATTATCCGCCTGATATGGTTGATCACTATAGTCCGGAAC<br>AGGATATGCTGAAAGAACAGGTGGTGAAATTTCTGGATATCCTGCCGAA<br>AAATCAGCGTGATGTTCTGCAGGCAGTTTATCTGGAAGAACTGCCGCATC<br>AGCAGGTTGCAGAACTGTTTGATATTCCGCTGGGCACCGTTAAAAGCCGT<br>CTGCGTCTGGCAGTTGAAAAACTGCGTCATAGCATGCATACCGAACAGCT<br>GAGGCCTGCAGCAAACGACGAAAACTACGCTGCTGCTGTTTAAGGATCC<br>AAGCTTGCGGCCGCACTCGAGCACCACCACCACCACCACTGA
**Terminator**	
T7term	TAGCATAACCCCTTGGGGCCTCTAAACGGGTCTTGAGGGGTTTTTTG
**Backbone elements**	
KanR	TTAGAAAAACTCATCGAGCATCAAATGAAACTGCAATTTATTCATATCAG<br>GATTATCAATACCATATTTTTGAAAAAGCCGTTTCTGTAATGAAGGAGAA<br>AACTCACCGAGGCAGTTCCATAGGATGGCAAGATCCTGGTATCGGTCTGC<br>GATTCCGACTCGTCCAACATCAATACAACCTATTAATTTCCCCTCGTCAA<br>AAATAAGGTTATCAAGTGAGAAATCACCATGAGTGACGACTGAATCCGG<br>TGAGAATGGCAAAAGTTTATGCATTTCTTTCCAGACTTGTTCAACAGGCC<br>AGCCATTACGCTCGTCATCAAAATCACTCGCATCAACCAAACCGTTATTC<br>ATTCGTGATTGCGCCTGAGCGAGACGAAATACGCGGTCGCTGTTAAAAG<br>GACAATTACAAACAGGAATCGAATGCAACCGGCGCAGGAACACTGCCAG<br>CGCATCAACAATATTTTCACCTGAATCAGGATATTCTTCTAATACCTGGA<br>ATGCTGTTTTCCCGGGGATCGCAGTGGTGAGTAACCATGCATCATCAGGA<br>GTACGGATAAAATGCTTGATGGTCGGAAGAGGCATAAATTCCGTCAGCC<br>AGTTTAGTCTGACCATCTCATCTGTAACATCATTGGCAACGCTACCTTTGC<br>CATGTTTCAGAAACAACTCTGGCGCATCGGGCTTCCCATACAATCGATAG<br>ATTGTCGCACCTGATTGCCCGACATTATCGCGAGCCCATTTATACCCATA<br>TAAATCAGCATCCATGTTGGAATTTAATCGCGGCCTAGAGCAAGACGTTT<br>CCCGTTGAATATGGCTCATACTCTTCCTTTTTCAATATTATTGAAGCATTT<br>ATCAGGGTTATTGTCTCATGAGCGGATACATATTTGAATGTATTTAGAAA<br>AATAAACAAA
AmpR	TCAAATATGTATCCGCTCATGAGACAATAACCCTGATAAATGCTTCAATA<br>ATATTGAAAAAGGAAGAGTATGAGTATTCAACATTTCCGTGTCGCCCTTA<br>TTCCCTTTTTTGCGGCATTTTGCCTTCCTGTTTTTGCTCACCCAGAAACGCT<br>GGTGAAAGTAAAAGATGCTGAAGATCAGTTGGGTGCACGAGTGGGTTAC<br>ATCGAACTGGATCTCAACAGCGGTAAGATCCTTGAGAGTTTTCGCCCCGA

	AGAACGTTTTCCAATGATGAGCACTTTTAAAGTTCTGCTATGTGGCGCGG<br>TATTATCCCGTGTTGACGCCGGGCAAGAGCAACTCGGTCGCCGCATACAC<br>TATTCTCAGAATGACTTGGTTGAGTACTCACCAGTCACAGAAAAGCATCT<br>TACGGATGGCATGACAGTAAGAGAATTATGCAGTGCTGCCATAACCATG<br>AGTGATAACACTGCGGCCAACTTACTTCTGACAACGATCGGAGGACCGA<br>AGGAGCTAACCGCTTTTTTGCACAACATGGGGGATCATGTAACTCGCCTT<br>GATCGTTGGGAACCGGAGCTGAATGAAGCCATACCAAACGACGAGCGTG<br>ACACCACGATGCCTGCAGCAATGGCAACAACGTTGCGCAAACTATTAAC<br>TGGCGAACTACTTACTCTAGCTTCCCGGCAACAATTAATAGACTGGATGG<br>AGGCGGATAAAGTTGCAGGACCACTTCTGCGCTCGGCCCTTCCGGCTGGC<br>TGGTTTATTGCTGATAAATCTGGAGCCGGTGAGCGTGGGTCTCGCGGTAT<br>CATTGCAGCACTGGGGCCAGATGGTAAGCCCTCCCGTATCGTAGTTATCT<br>ACACGACGGGGAGTCAGGCAACTATGGATGAACGAAATAGACAGATCGC<br>TGAGATAGGTGCCTCACTGATTAAGCATTGGTAA
SpR	TTATTTGCCGACTACCTTGGTGATCTCGCCTTTCACGTAGTGGACAAATTC<br>TTCCAACTGATCTGCGCGCGAGGCCAAGCGATCTTCTTCTTGTCCAAGAT<br>AAGCCTGTCTAGCTTCAAGTATGACGGGCTGATACTGGGCCGGCAGGCG<br>CTCCATTGCCCAGTCGGCAGCGACATCCTTCGGCGCGATTTTGCCGGTTA<br>CTGCGCTGTACCAAATGCGGGACAACGTAAGCACTACATTTCGCTCATCG<br>CCAGCCCAGTCGGGCGGCGAGTTCCATAGCGTTAAGGTTTCATTTAGCGC<br>CTCAAATAGATCCTGTTCAGGAACCGGATCAAAGAGTTCCTCCGCCGCTG<br>GACCTACCAAGGCAACGCTATGTTCTCTTGCTTTTGTCAGCAAGATAGCC<br>AGATCAATGTCGATCGTGGCTGGCTCGAAGATACCTGCAAGAATGTCATT<br>GCGCTGCCATTCTCCAAATTGCAGTTCGCGCTTAGCTGGATAACGCCACG<br>GAATGATGTCGTCGTGCACAACAATGGTGACTTCTACAGCGCGGAGAAT<br>CTCGCTCTCTCCAGGGGAAGCCGAAGTTTCCAAAAGGTCGTTGATCAAAG<br>CTCGCCGCGTTGTTTCATCAAGCCTTACGGTCACCGTAACCAGCAAATCA<br>ATATCACTGTGTGGCTTCAGGCCGCCATCCACTGCGGAGCCGTACAAATG<br>TACGGCCAGCAACGTCGGTTCGAGATGGCGCTCGATGACGCCAACTACC<br>TCTGATAGTTGAGTCGATACTTCGGCGATCACCGCTTCCCTCATACTCTTC<br>CTTTTTCAATATTATTGAAGCATTTATCAGGGTTATTGTCTCATGAGCGGA<br>TACATATTTGAATGTATTTAGAAAAATAAACAAA
ChlR	TTACGCCCCGCCCTGCCACTCATCGCAGTACTGTTGTAATTCATTAAGCAT<br>TCTGCCGACATGGAAGCCATCACAAACGGCATGATGAACCTGAATCGCC<br>AGCGGCATCAGCACCTTGTCGCCTTGCGTATAATATTTGCCCATGGTGAA<br>AACGGGGGCGAAGAAGTTGTCCATATTGGCCACGTTTAAATCAAAACTG<br>GTGAAACTCACCCAGGGATTGGCTGAGACGAAAAACATATTCTCAATAA<br>ACCCTTTAGGGAAATAGGCCAGGTTTTCACCGTAACACGCCACATCTTGC<br>GAATATATGTGTAGAAACTGCCGGAAATCGTCGTGGTATTCACTCCAGAG<br>CGATGAAAACGTTTCAGTTTGCTCATGGAAAACGGTGTAACAAGGGTGA<br>ACACTATCCCATATCACCAGCTCACCGTCTTTCATTGCCATACGGAATTC<br>CGGATGAGCATTCATCAGGCGGGCAAGAATGTGAATAAAGGCCGGATAA<br>AACTTGTGCTTATTTTTCTTTACGGTCTTTAAAAAGGCCGTAATATCCAGC<br>TGAACGGTCTGGTTATAGGTACATTGAGCAACTGACTGAAATGCCTCAAA<br>ATGTTCTTTACGATGCCATTGGGATATATCAACGGTGGTATATCCAGTGA<br>TTTTTTTCTCCATTTTAGCTTCCTTAGCTCCTGAAAATCTCGATAACTCAA<br>AAAATACGCCCGGTAGTGATCTTATTTCATTATGGTGAAAGTTGGAACCT<br>CTTACGTGCCGATCA
ColA	AAACGTCCTAGAAGATGCCAGGAGGATACTTAGCAGAGAGACAATAAGG<br>CCGGAGCGAAGCCGTTTTTCCATAGGCTCCGCCCCCCTGACGAACATCAC<br>GAAATCTGACGCTCAAATCAGTGGTGGCGAAACCCGACAGGACTATAAA<br>GATACCAGGCGTTTCCCCCTGATGGCTCCCTCTTGCGCTCTCCTGTTCCCG<br>TCCTGCGGCGTCCGTGTTGTGGTGGAGGCTTTACCCAAATCACCACGTCC<br>CGTTCCGTGTAGACAGTTCGCTCCAAGCTGGGCTGTGTGCAAGAACCCCC<br>CGTTCAGCCCGACTGCTGCGCCTTATCCGGTAACTATCATCTTGAGTCCA

	ACCCGGAAAGACACGACAAAACGCCACTGGCAGCAGCCATTGGTAACTG<br>AGAATTAGTGGATTTAGATATCGAGAGTCTTGAAGTGGTGGCCTAACAG<br>AGGCTACACTGAAAGGACAGTATTTGGTATCTGCGCTCCACTAAAGCCAG<br>TTACCAGGTTAAGCAGTTCCCCAACTGACTTAACCTTCGATCAAACCGCC<br>TCCCCAGGCGGTTTTTTCGTTTACAGAGCAGGAGATTACGACGATCGTAA<br>AAGGATCTCAAGAAGATCCTTTACGGATTCCCGACACCA
pBR322	TTGAGATCCTTTTTTTCTGCGCGTAATCTGCTGCTTGCAAACAAAAAAAC<br>CACCGCTACCAGCGGTGGTTTGTTTGCCGGATCAAGAGCTACCAACTCTT<br>TTTCCGAAGGTAACTGGCTTCAGCAGAGCGCAGATACCAAATACTGTCCT<br>TCTAGTGTAGCCGTAGTTAGGCCACCACTTCAAGAACTCTGTAGCACCGC<br>CTACATACCTCGCTCTGCTAATCCTGTTACCAGTGGCTGCTGCCAGTGGC<br>GATAAGTCGTGTCTTACCGGGTTGGACTCAAGACGATAGTTACCGGATAA<br>GGCGCAGCGGTCGGGCTGAACGGGGGGTTCGTGCACACAGCCCAGCTTG<br>GAGCGAACGACCTACACCGAACTGAGATACCTACAGCGTGAGCTATGAG<br>AAAGCGCCACGCTTCCCGAAGGGAGAAAGGCGGACAGGTATCCGGTAAG<br>CGGCAGGGTCGGAACAGGAGAGCGCACGAGGGAGCTTCCAGGGGGAAA<br>CGCCTGGTATCTTTATAGTCCTGTCGGGTTTCGCCACCTCTGACTTGAGCG<br>TCGATTTTTGTGATGCTCGTCAGGGGGGCGGAGCCTATGGAA
CloDF13	GCGCTGCGGACACATACAAAGTTACCCACAGATTCCGTGGATAAGCAGG<br>GGACTAACATGTGAGGCAAAACAGCAGGGCCGCGCCGGTGGCGTTTTTC<br>CATAGGCTCCGCCCTCCTGCCAGAGTTCACATAAACAGACGCTTTTCCGG<br>TGCATCTGTGGGAGCCGTGAGGCTCAACCATGAATCTGACAGTACGGGC<br>GAAACCCGACAGGACTTAAAGATCCCCACCGTTTCCGGCGGGTCGCTCCC<br>TCTTGCGCTCTCCTGTTCCGACCCTGCCGTTTACCGGATACCTGTTCCGCC<br>TTTCTCCCTTACGGGAAGTGTGGCGCTTTCTCATAGCTCACACACTGGTAT<br>CTCGGCTCGGTGTAGGTCGTTCGCTCCAAGCTGGGCTGTAAGCAAGAACT<br>CCCCGTTCAGCCCGACTGCTGCGCCTTATCCGGTAACTGTTCACTTGAGT<br>CCAACCCGGAAAAGCACGGTAAAACGCCACTGGCAGCAGCCATTGGTAA<br>CTGGGAGTTCGCAGAGGATTTGTTTAGCTAAACACGCGGTTGCTCTTGAA<br>GTGTGCGCCAAAGTCCGGCTACACTGGAAGGACAGATTTGGTTGCTGTGC<br>TCTGCGAAAGCCAGTTACCACGGTTAAGCAGTTCCCCAACTGACTTAACC<br>TTCGATCAAACCACCTCCCCAGGTGGTTTTTTCGTTTACAGGGCAAAAGA<br>TTACGCGCAGAAAAAAAGGATCTCAAGAAGATCCTTTGATC
p15A	TTTCCATAGGCTCCGCCCCCCTGACAAGCATCACGAAATCTGACGCTCAA<br>ATCAGTGGTGGCGAAACCCGACAGGACTATAAAGATACCAGGCGTTTCC<br>CCCTGGCGGCTCCCTCGTGCGCTCTCCTGTTCCTGCCTTTCGGTTTACCGG<br>TGTCATTCCGCTGTTATGGCCGCGTTTGTCTCATTCCACGCCTGACACTCA<br>GTTCCGGGTAGGCAGTTCGCTCCAAGCTGGACTGTATGCACGAACCCCCC<br>GTTCAGTCCGACCGCTGCGCCTTATCCGGTAACTATCGTCTTGAGTCCAA<br>CCCGGAAAGACATGCAAAAGCACCACTGGCAGCAGCCACTGGTAATTGA<br>TTTAGAGGAGTTAGTCTTGAAGTCATGCGCCGGTTAAGGCTAAACTGAAA<br>GGACAAGTTTTGGTGACTGCGCTCCTCCAAGCCAGTTACCTCGGTTCAAA<br>GAGTTGGTAGCTCAGAGAACCTTCGAAAAACCGCCCTGCAAGGCGGTTTT<br>TTCGTTTTCAGAGCAAGAGATTACGCGCAGACCAAAACGATCTCAA
LacI	TCACTGCCCGCTTTCCAGTCGGGAAACCTGTCGTGCCAGCTGCATTAATG<br>AATCGGCCAACGCGCGGGGAGAGGCGGTTTGCGTATTGGGCGCCAGGGT<br>GGTTTTTCTTTTCACCAGTGAGACGGGCAACAGCTGATTGCCCTTCACCG<br>CCTGGCCCTGAGAGAGTTGCAGCAAGCGGTCCACGCTGGTTTGCCCCAGC<br>AGGCGAAAATCCTGTTTGATGGTGGTTAACGGCGGGATATAACATGAGC<br>TGTCTTCGGTATCGTCGTATCCCACTACCGAGATATCCGCACCAACGCGC<br>AGCCCGGACTCGGTAATGGCGCGCATTGCGCCCAGCGCCATCTGATCGTT<br>GGCAACCAGCATCGCAGTGGGAACGATGCCCTCATTCAGCATTTGCATGG<br>TTTGTTGAAAACCGGACATGGCACTCCAGTCGCCTTCCCGTTCCGCTATC<br>GGCTGAATTTGATTGCGAGTGAGATATTTATGCCAGCCAGCCAGACGCAG<br>ACGCGCCGAGACAGAACTTAATGGGCCCGCTAACAGCGCGATTTGCTGG

	TGACCCAATGCGACCAGATGCTCCACGCCCAGTCGCGTACCGTCTTCATG GGAGAAAATAATACTGTTGATGGGTGTCTGGTCAGAGACATCAAGAAAT AACGCCGGAACATTAGTGCAGGCAGCTTCCACAGCAATGGCATCCTGGT CATCCAGCGGATAGTTAATGATCAGCCCACTGACGCGTTGCGCGAGAAG ATTGTGCACCGCCGCTTTACAGGCTTCGACGCCGCTTCGTTCTACCATCG ACACCACCACGCTGGCACCCAGTTGATCGGCGCGAGATTTAATCGCCGC GACAATTTGCGACGGCGCGTGCAGGGCCAGACTGGAGGTGGCAACGCCA ATCAGCAACGACTGTTTGCCCGCCAGTTGTTGTGCCACGCGGTTGGGAAT GTAATTCAGCTCCGCCATCGCCGCTTCCACTTTTTCCCGCGTTTTCGCAGA AACGTGGCTGGCCTGGTTCACCACGCGGGAAACGGTCTGATAAGAGACA CCGGCATACTCTGCGACATCGTATAACGTTACTGGTTTCACATTCACCAC CCTGAATTGACTCTCTTCCGGGCGCTATCATGCCATACCGCGAAAGGTTT TGCGCCATTCGATGGTGTC

Supplementary Figures

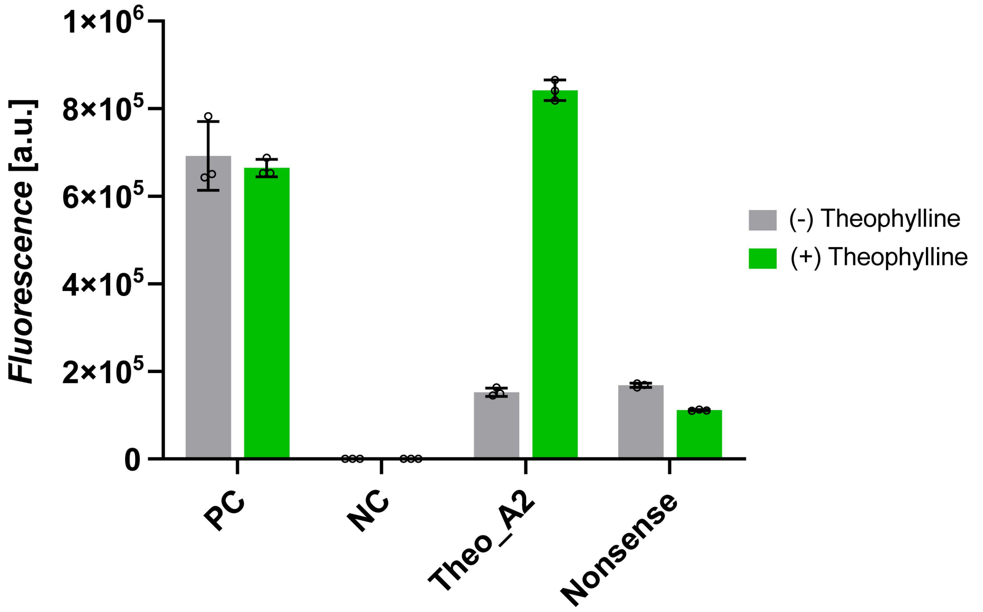

Figure S1. Comparative analysis with nonsense RNA construct

GFP fluorescence of Theo_A2 compared with nonsense RNA in the absence (gray bars) and presence (green bars) of 5 mM theophylline. Positive control (PC) used the toehold switch trigger A without the aptamer sequence, and negative control (NC) used the decoy RNA without any predicted interactions. Nonsense RNA construct contains a sequence of the same length as the theophylline aptamer but lacks ligand-binding capabilities, inserted into the trigger RNA. Error bars are the standard deviation (SD) from three biological replicates.

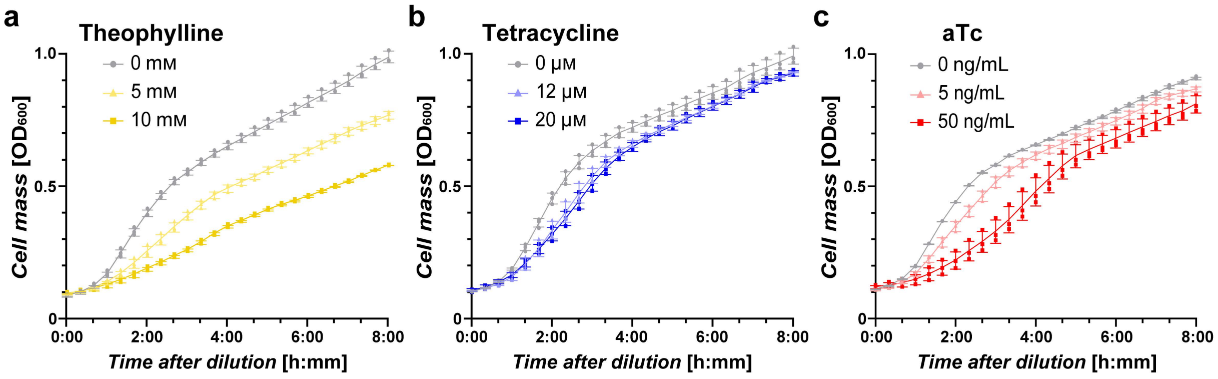

Figure S2. Growth curves of *E. coli* strain BL21 with ligand treatment

a-c) Growth curves of *E. coli* strain BL21 in presence or absence of (a) theophylline, (b) tetracycline, and (c) anhydrotetracycline (aTc). Growth was measured by tracking optical density at 600 nm wavelength (OD_{600}) over time. Error bars are the SD from three biological replicates.

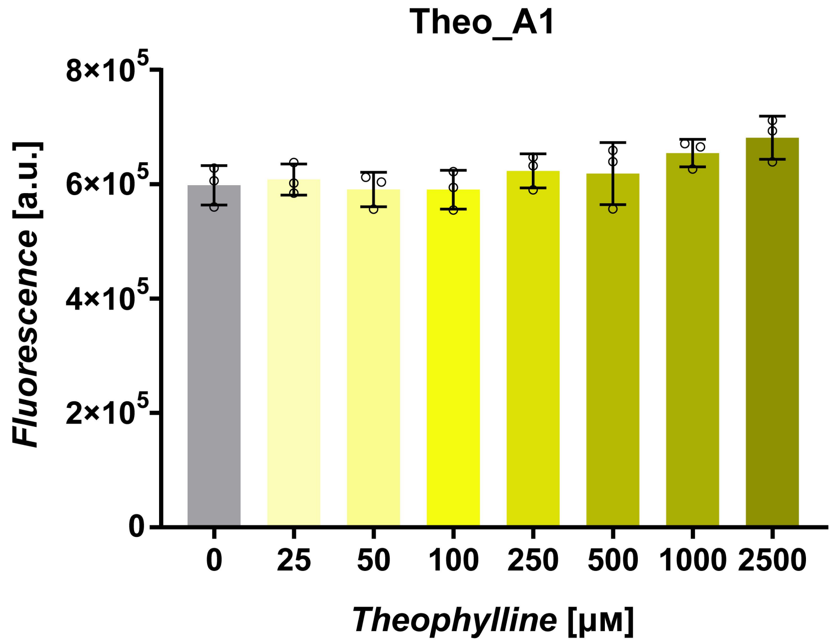

Figure S3. GFP fluorescence with theophylline Apta-trigger Theo_A1

GFP fluorescence of theophylline START with Theo_A1, utilizing the reported theophylline aptamer sequence[9] without any modification. Error bars are the standard deviation (SD) from three biological replicates.

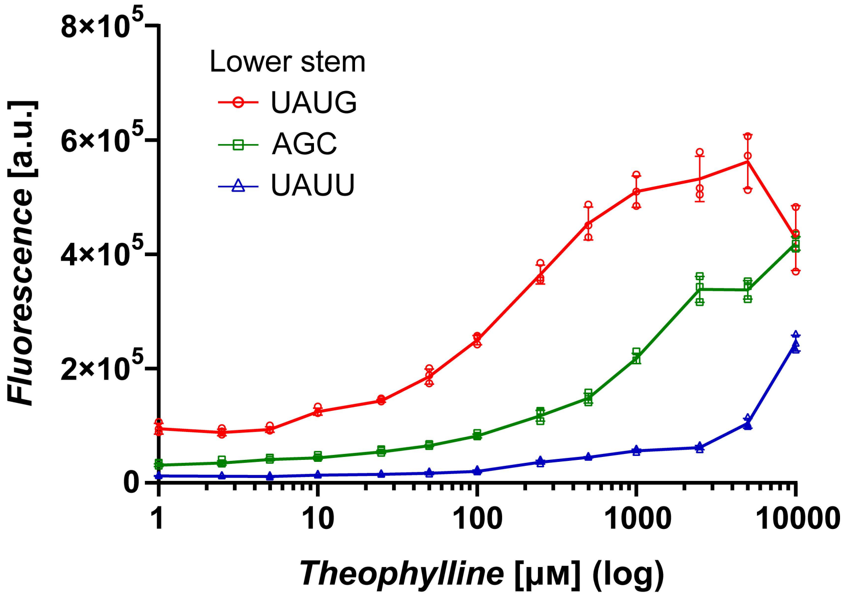

Figure S4. Dose-response curves of stem-modified theophylline Apta-triggers

Dose-response curves of Theo_A2 (UAUG), Theo_A7 (AGC), and Theo_A11 (UAUU). Results were min-max normalized in Figure 4d. Error bars are the SD from three biological replicates.

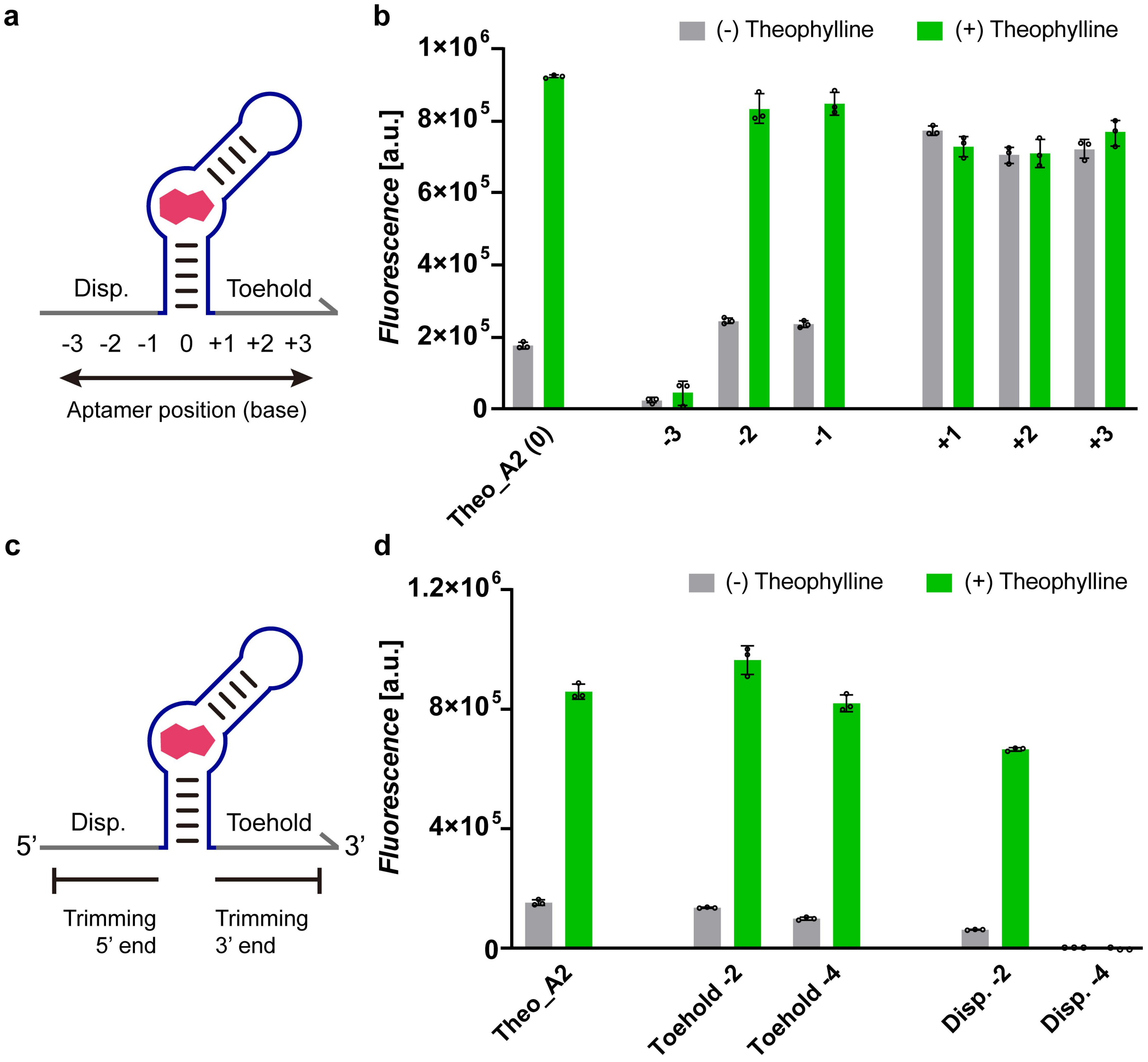

Figure S5. Design optimization of partial trigger domains for dynamic range improvement

a) Schematic for screening aptamer positions. The aptamer position was shifted by 1 base in each direction, up to a maximum of 3 bases, altering the length of displacement domain (Disp.) and toehold binding domain (Toehold) (e.g., -2 shift changes 13(Disp.)-13(Toehold) bases to 11-15 bases). b) GFP fluorescence of aptamer shifted Theo_A2 in the absence (gray bars) and presence (green bars) of 5 mM theophylline. Each index indicates the following constructs; -3: Theo_A2_shift1, -2: Theo_A2_shift2, -1: Theo_A2_shift3, +1: Theo_A2_shift4, +2: Theo_A2_shift5, +3: Theo_A2_shift6. c) Schematic for trimming the length of partial trigger domains. The toehold binding domain or displacement domain was trimmed at their 3' or 5' ends respectively, by 2-bases (-2) or 4-bases (-4). d) GFP fluorescence of trimmed Theo_A2 in the absence and presence of 5 mM theophylline. Each index indicates the following constructs; Toehold -2: Theo_A2_trim1, Toehold -4: Theo_A2_trim2, Disp. -2: Theo_A2_trim3, Disp. -4: Theo_A2_trim4. Error bars are the SD from three biological replicates.

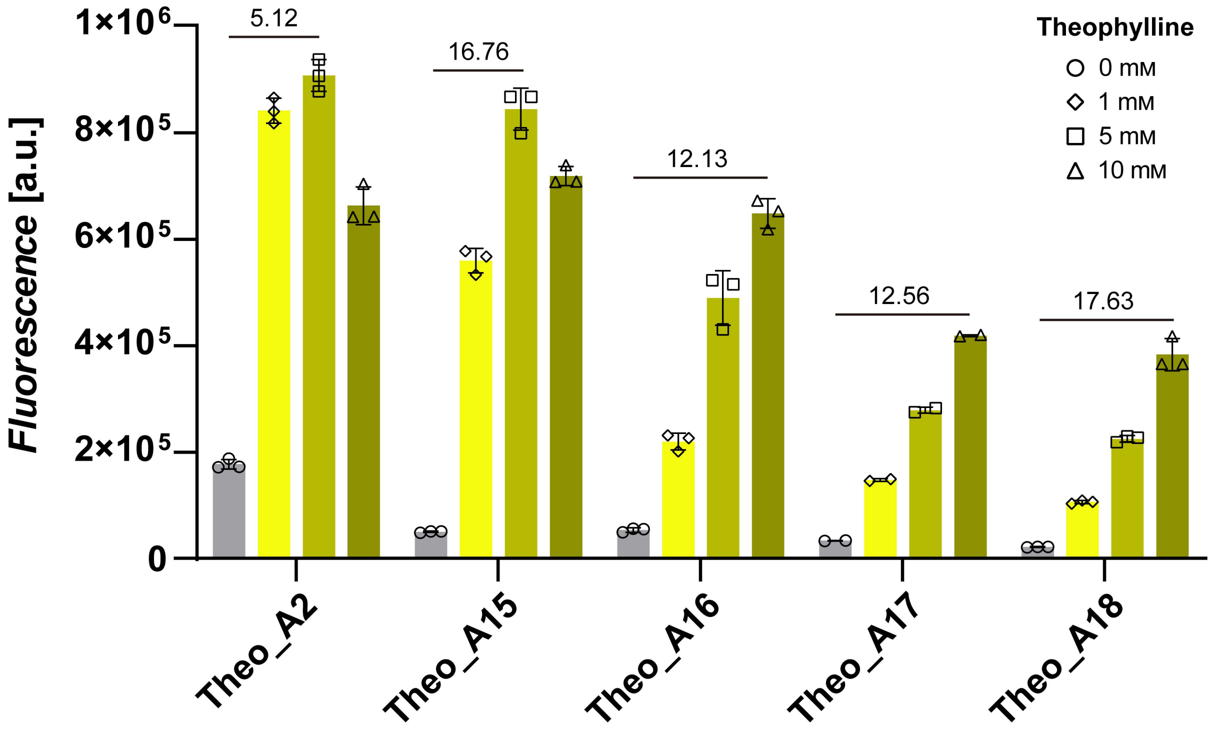

Figure S6. Optimized theophylline Apta-triggers and their dynamic range

GFP fluorescence with optimized theophylline Apta-triggers, Theo_A15 to Theo_A18. Modified features for each construct are as follows: Theo_A15-toehold domain 4-base trimming + displacement domain 2-base trimming, Theo_A16-toehold domain 4-base trimming + UAUU (lower stem modification), Theo_A17-displacement domain 2-base trimming + UAUU, Theo_A18-toehold domain 4-base trimming + displacement domain 2-base trimming + UAUU. Fold change (numbers above the bar graph) was calculated with the highest fluorescence observed under the given conditions. Error bars are the SD from three biological replicates.

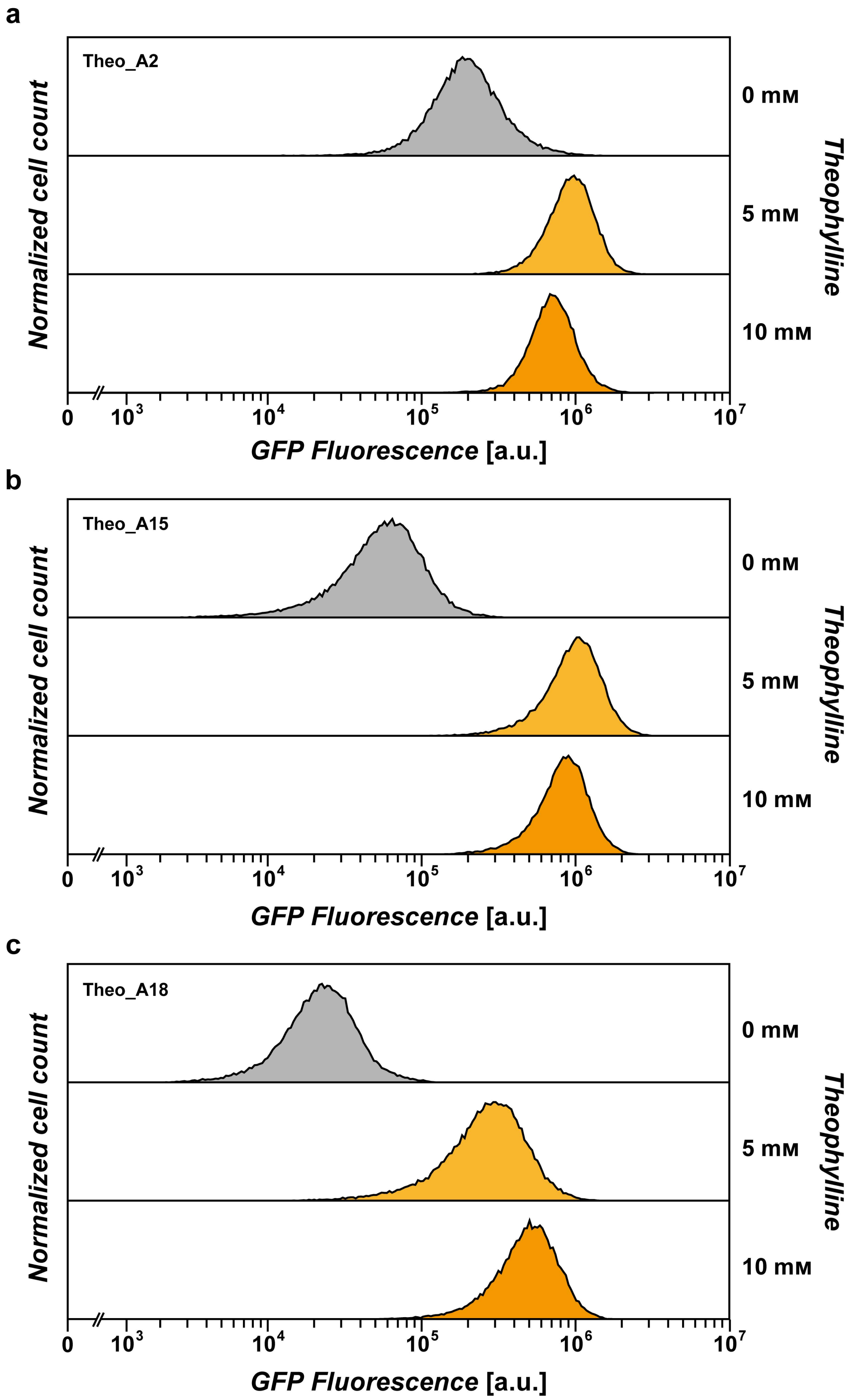

Figure S7. Flow cytometry histograms with the optimized theophylline Apta-triggers

a-c) Flow cytometry histograms of GFP fluorescence with (a) Theo_A2, (b) Theo_A15, and (c) Theo_A18. Cells were treated with 0 mM (top panel), 5 mM (middle panel), or 10 mM (bottom panel) theophylline, and a total ~ 50,000 individual cells analyzed per condition.

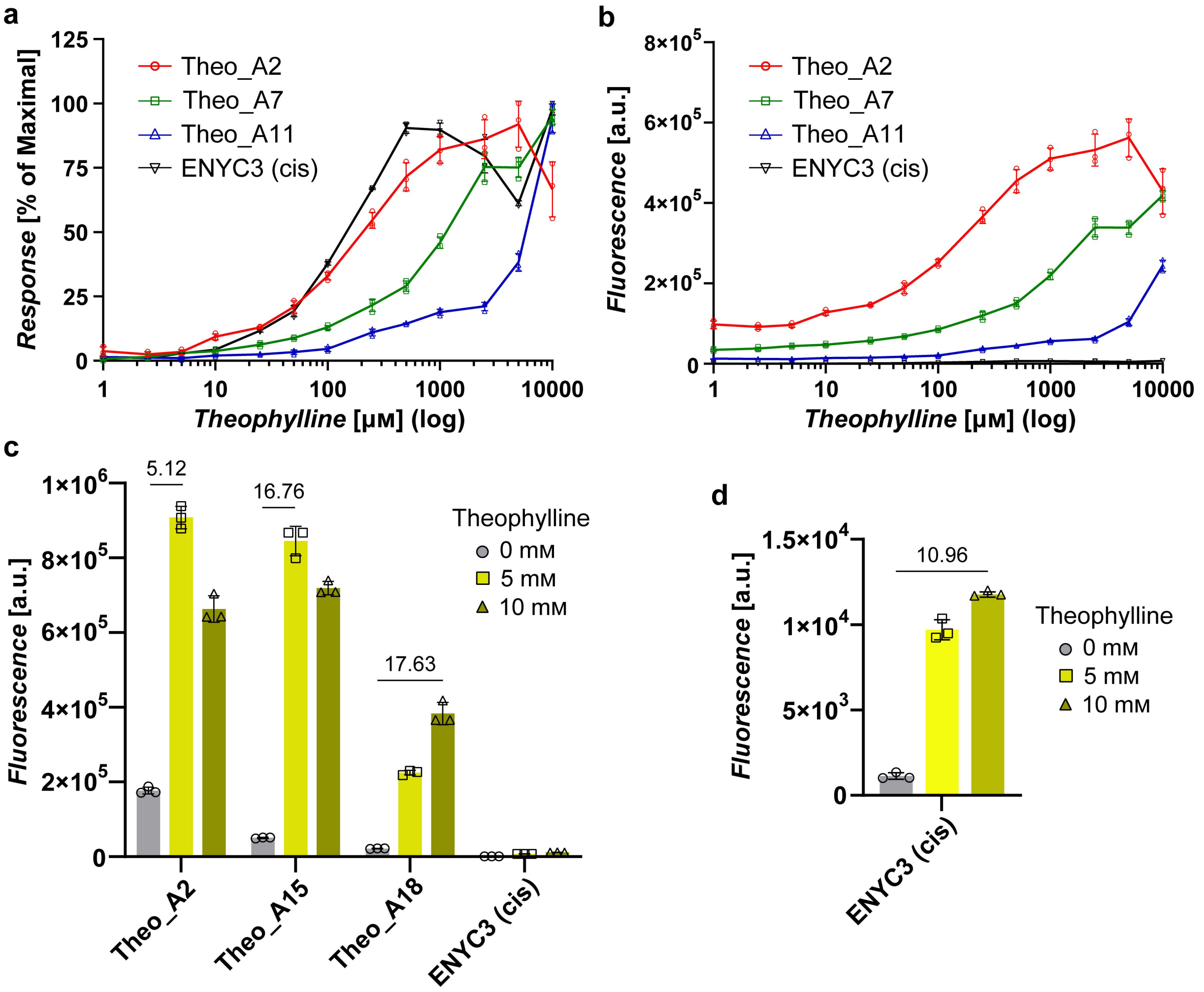

Figure S8. Comparative analysis with cis-acting theophylline riboswitch

a-b) Dose-response curves of theophylline Apta-trigger variants compared with cis-acting theophylline riboswitch (ENYC3) reported by Nakahira et al.,[10] with min-max normalization (a) and without normalization (b). c) GFP fluorescence of theophylline Apta-trigger variants compared with ENYC3. d) Fluorescence offset for ENYC3. Fold change (number above the bar graph) was calculated with the highest fluorescence observed under the given conditions. Error bars are the SD from three biological replicates.

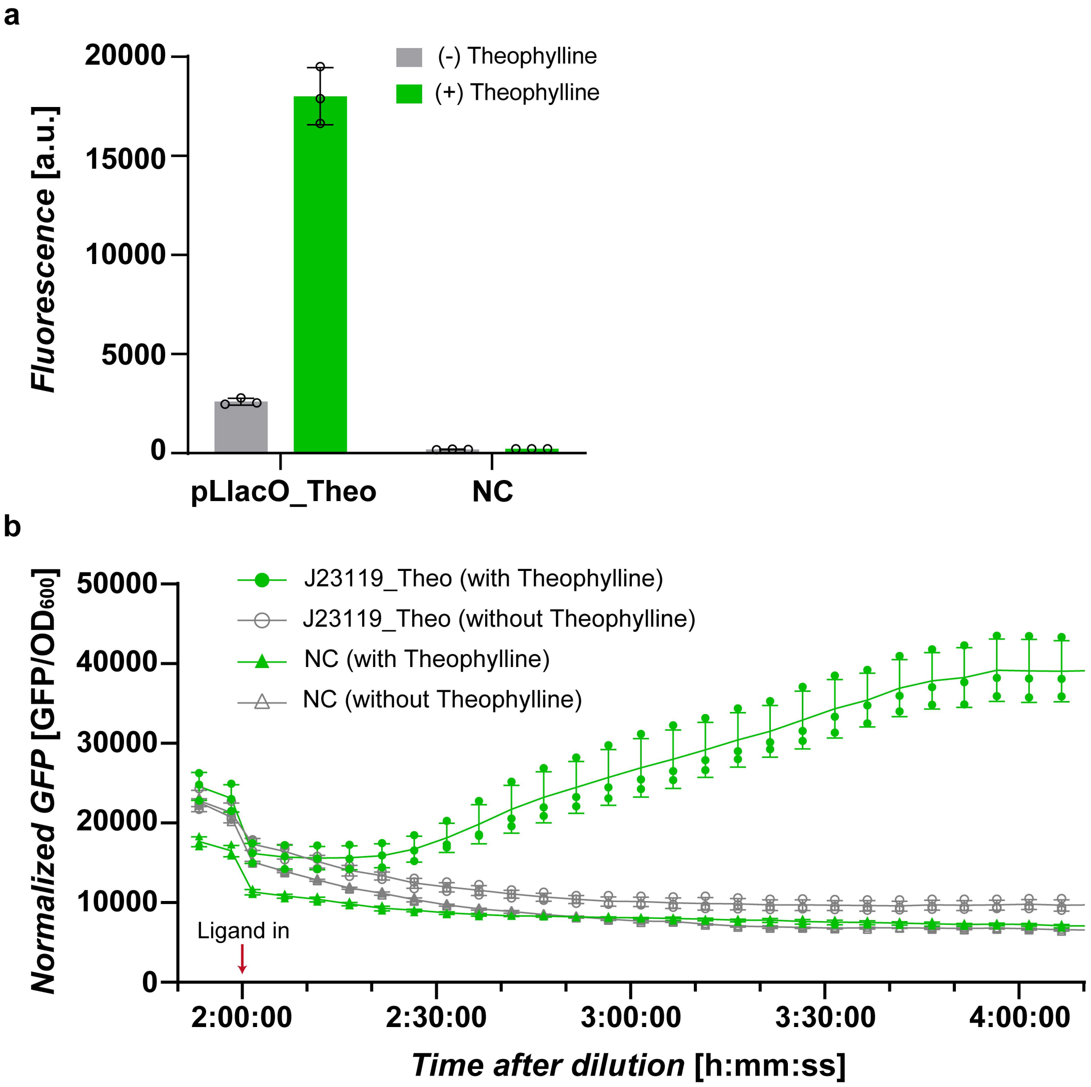

Figure S9. START characterization with different promoters

a) GFP fluorescence of theophylline START with pLlacO promoter (including both switch RNA and Apta-trigger) in the absence and presence of 10 mM theophylline. b) Time-course measurements of fluorescence with synthetic constitutive promoter, J23119.[11] 10 mM of theophylline was treated at the time-point of 2 hours after 1/100-fold dilution. Fluorescence was normalized with cell growth (OD_{600}). Error bars are the SD from three biological replicates.

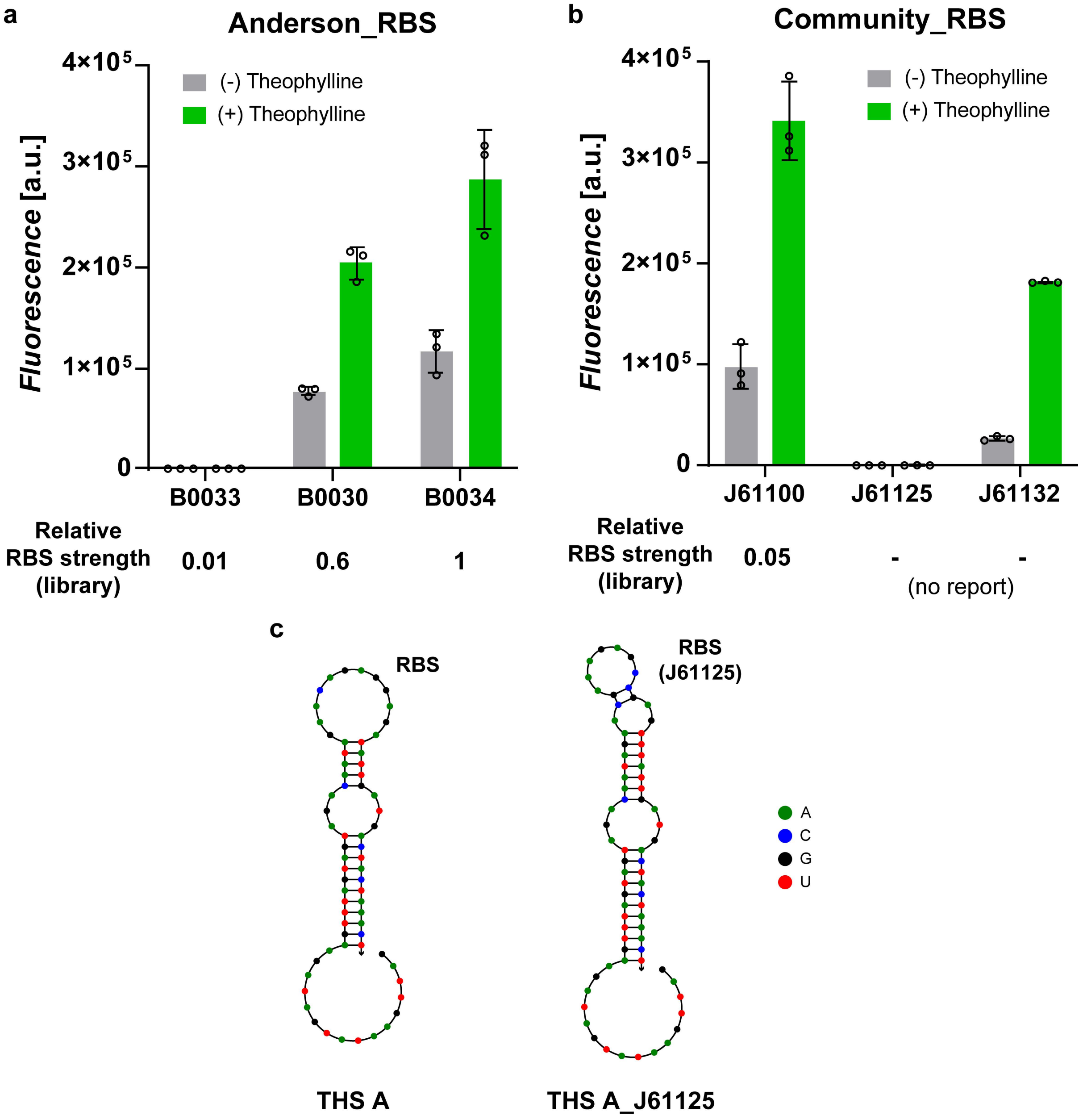

Figure S10. START characterization with different ribosome binding sites

a, b) GFP fluorescence of theophylline STARTs with (a) Anderson family ribosome binding site (RBS)[12] or (b) Community RBS.[13] Relative RBS strengths are indicated below the graph when the data was available. Gray and green bars indicate GFP expression in the absence and presence of 1 mM theophylline, respectively. Error bars are the SD from three biological replicates. c) Predicted minimum free-energy (MFE) structure of THS A with the original RBS (left panel) and with J61125 RBS (right panel), by NUPACK.

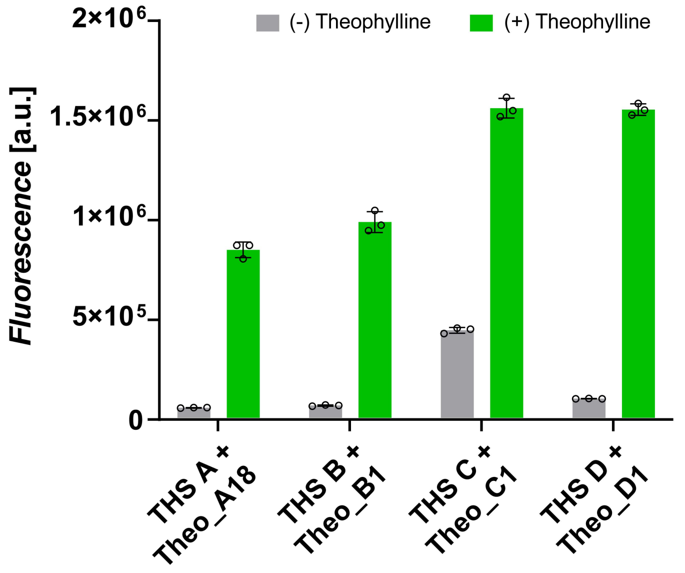

Figure S11. Theophylline START constructs utilizing toehold switch library

Characterization of theophylline START constructs utilizing switch and trigger RNA sequences from the toehold switch library reported by Kim et al.[5] Identical theophylline aptamer sequences where the lower stem is slightly modulated to prevent unwanted interactions were used. Gray and green bars indicate GFP fluorescence in the absence and presence of 10 mM theophylline, respectively. Error bars are the SD from three biological replicates.

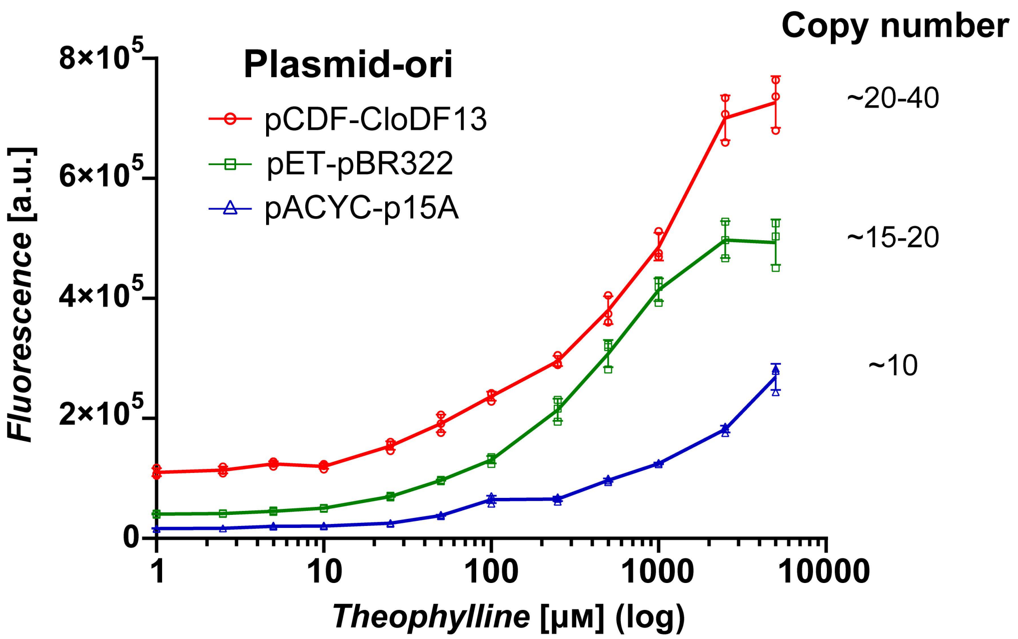

Figure S12. Characterization of theophylline Apta-triggers with different plasmid copy number

Dose-response curves of theophylline Apta-triggers (Theo_A15) in different plasmid backbones with different replicate origin. Estimated copy numbers for each plasmid backbone-replicate origin are indicated on the right side of corresponding curves.[14] Error bars are the SD from three biological replicates.

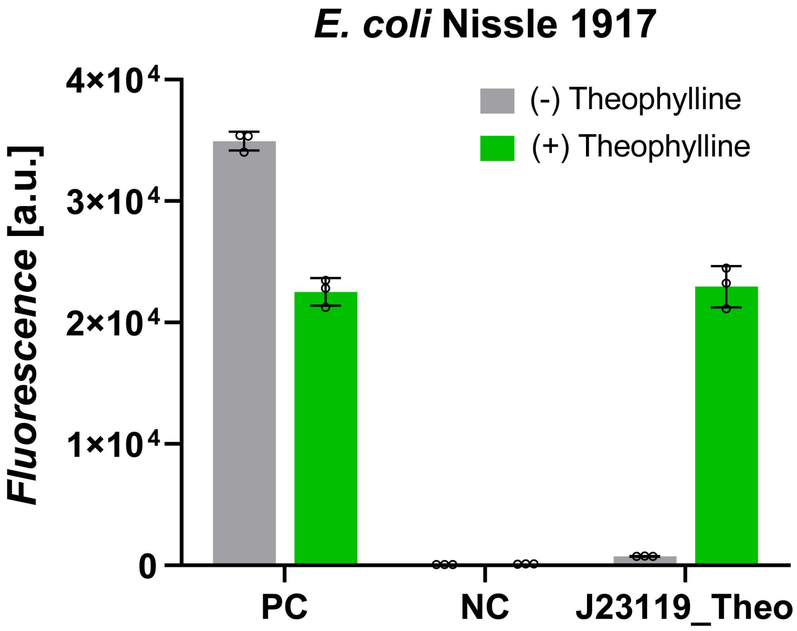

Figure S13. START characterization in *E. coli* strain Nissle 1917

GFP fluorescence of theophylline START in probiotic *E. coli* strain Nissle 1917 in the absence (gray bars) and presence (green bars) of 10 mM theophylline. Positive control (PC) used the toehold switch trigger without the aptamer sequence, and negative control (NC) used the decoy RNA without any predicted interactions. Error bars represent the SD from three biological replicates.

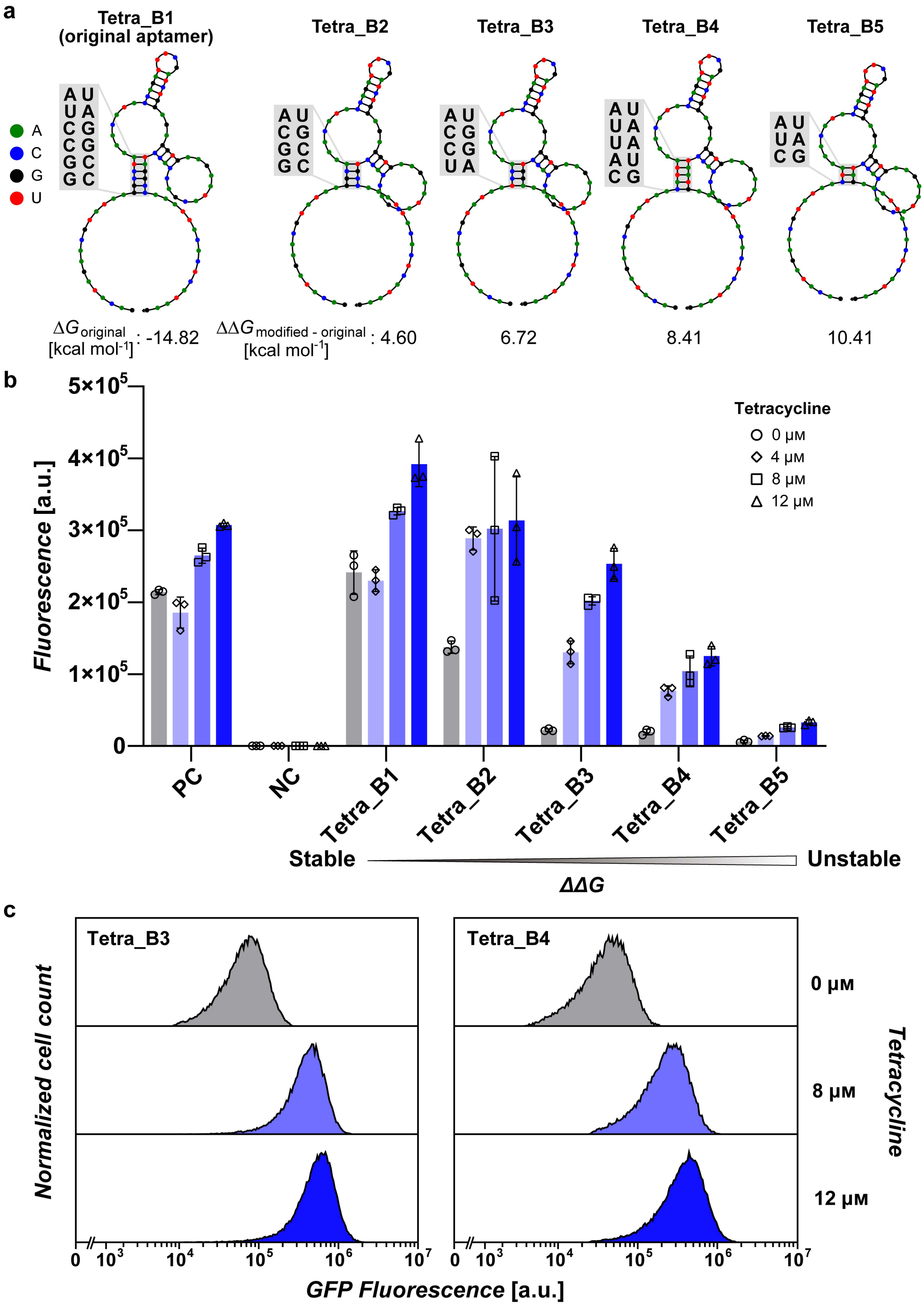

Figure S14. Design and characterization of tetracycline STARTs

a) Predicted secondary structure and free-energy difference of tetracycline Apta-triggers with previously reported tetracycline aptamer (Tetra_B1),[15] and lower stem-modified tetracycline aptamers (Tetra_B2~B5). Each lower stem sequence is indicated in a gray box. b) GFP fluorescence of tetracycline Apta-trigger variants with different concentrations of tetracycline. Positive control (PC) used the toehold switch trigger B without the aptamer sequence, while negative control (NC) used the decoy RNA without any predicted interactions. Error bars are the SD from three biological replicates. c) Flow cytometry histograms of GFP fluorescence with Tetra_B3 (left panel) and Tetra_B4 (right panel). Total ~ 50,000 individual cells were analyzed per condition.

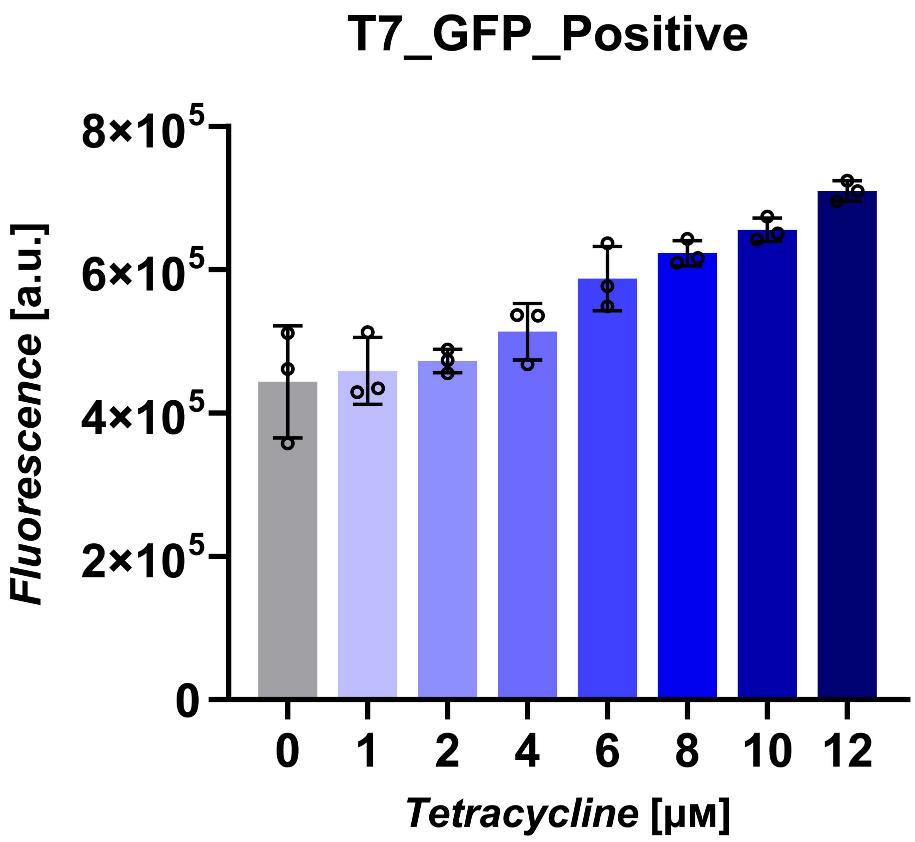

Figure S15. GFP positive control with tetracycline treatment

Fluorescence of GFP positive control in the presence of different concentrations of tetracycline. GFP positive control contained GFPmut3b with T7 promoter, RBS, and T7 terminator without switch sequence. Error bars are the SD from three biological replicates.

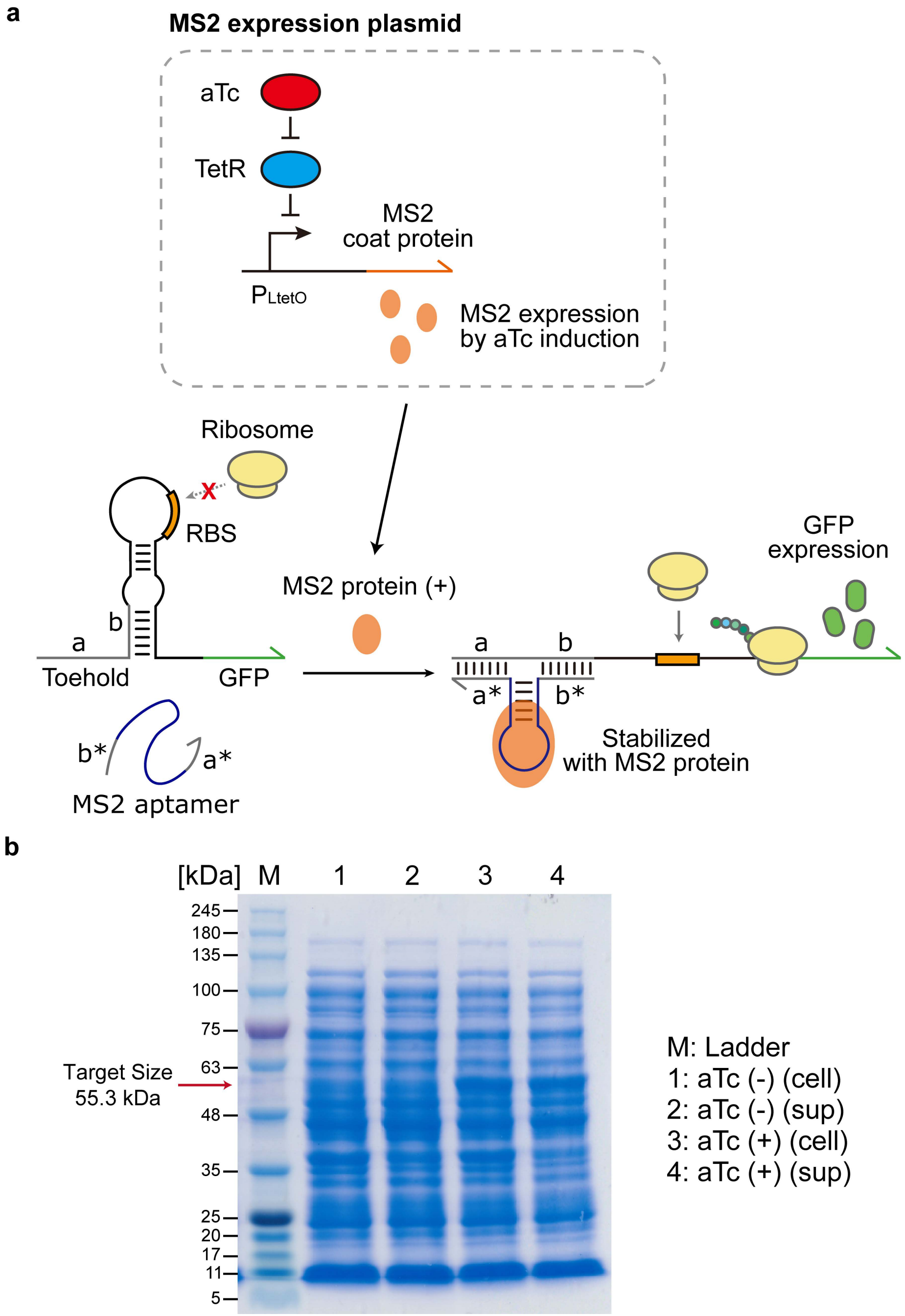

Figure S16. In vivo expression of MS2 coat protein

a) Experimental schematics of START for in vivo expressed MS2 coat protein. The promoter for MS2 coat protein expression is repressed by tetR. Upon treatment with anhydrotetracycline (aTc), tetR is inactivated, leading to in vivo MS2 protein expression. b) SDS-PAGE gel running results for the MS2 protein expression test. For the test, only the MS2 expression plasmid was introduced into BL21 DE3 strain without the switch and trigger constructs. 10 ng mL^{-1} aTc was treated and other culture conditions were the same as the MS2 START constructs characterization depicted in the main text. A total of 5 mL of cells were collected for each condition (aTc treated / not treated), diluted in 1 mL of lysis buffer, and sonicated. Each lysate was loaded as cell samples (in line 1 and 3), and its centrifuged supernatant was loaded as sup samples (in line 2 and 4).

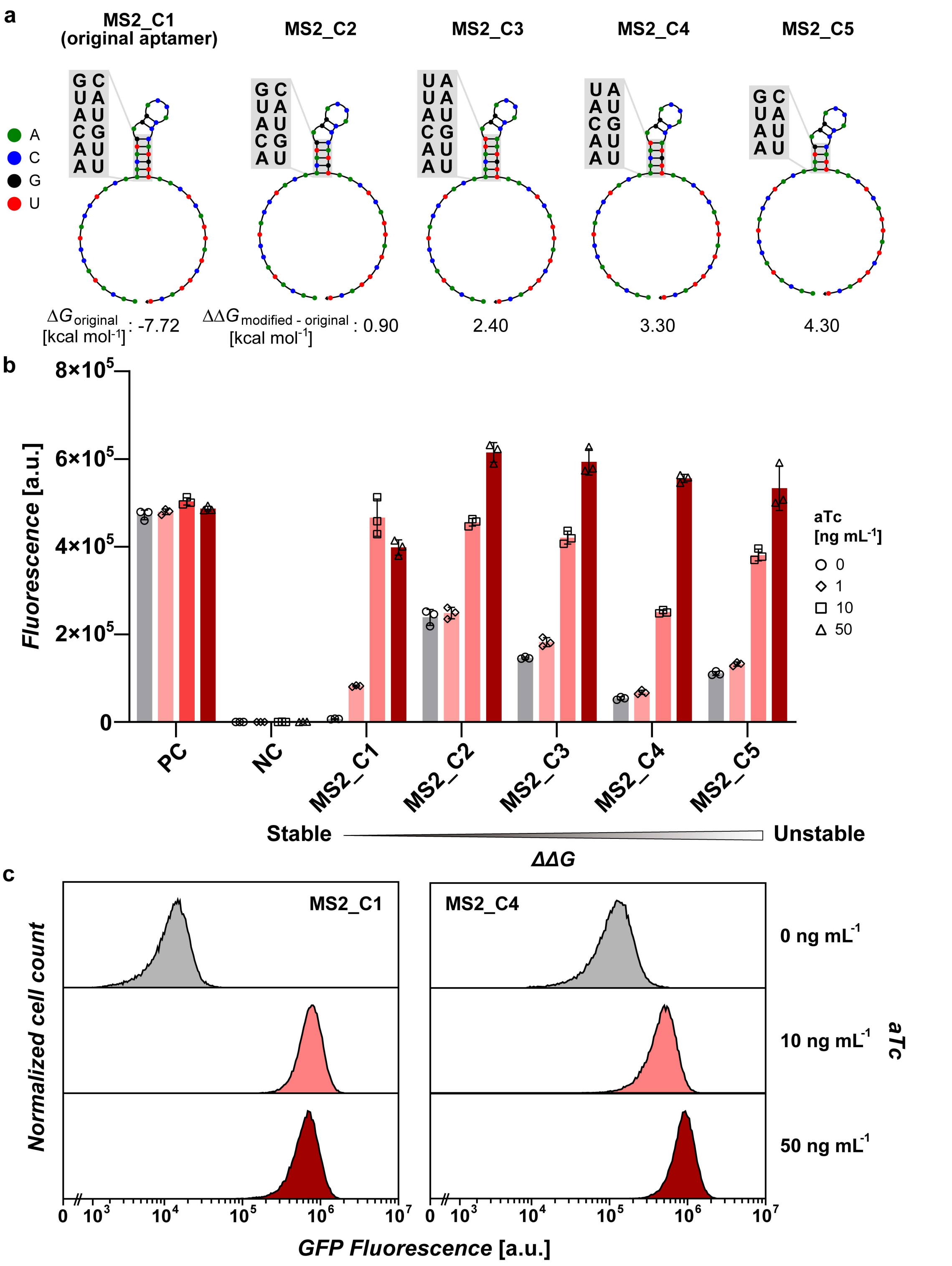

Figure S17. Design and characterization of MS2 STARTs

a) Predicted secondary structure and free-energy difference of MS2 Apta-triggers with previously reported MS2 coat protein aptamer (MS2_C1),[16] and lower stem-modified MS2 aptamers (MS2_C2~C5). Each lower stem sequence is indicated in a gray box. b) GFP fluorescence of MS2 Apta-trigger variants with different strength of MS2 induction. Positive control (PC) used the toehold switch trigger C without the aptamer sequence, while negative control (NC) used the decoy RNA without any predicted interactions. Error bars are the SD from three biological replicates. c) Flow cytometry histograms of GFP fluorescence with MS2_C1 (left panel) and MS2_C4 (right panel). Total ~ 50,000 individual cells were analyzed per condition.

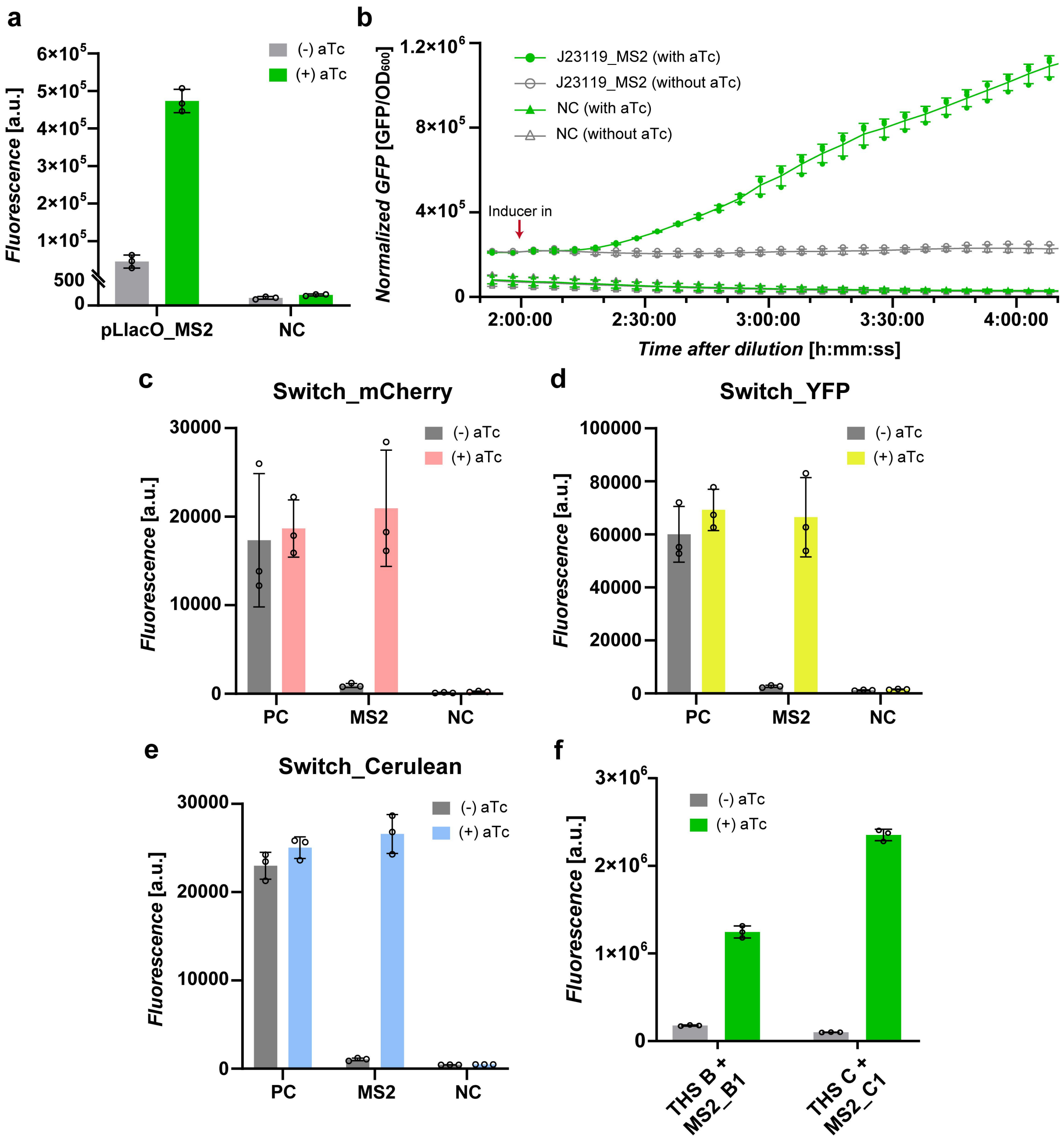

Figure S18. Characterization of MS2 START constructs across different genetic contexts

a) GFP fluorescence of MS2 START construct with the pLlacO promoter in the absence and presence of MS2 protein expression. b) Time-course measurements of fluorescence with the J23119 promoter. MS2 protein induction was conducted at the time-point of 2 hours after 100-fold dilution. Fluorescence was normalized with OD_{600} value. c-e) Downstream gene of switch RNA was replaced to (c) mCherry, (d) yellow fluorescent protein (YFP), or (e) Cerulean. f) MS2 START constructs utilizing the toehold switch library. Throughout all experiments, aTc for MS2 expression was treated at 10 ng mL^{-1}. Error bars are the SD from three biological replicates.

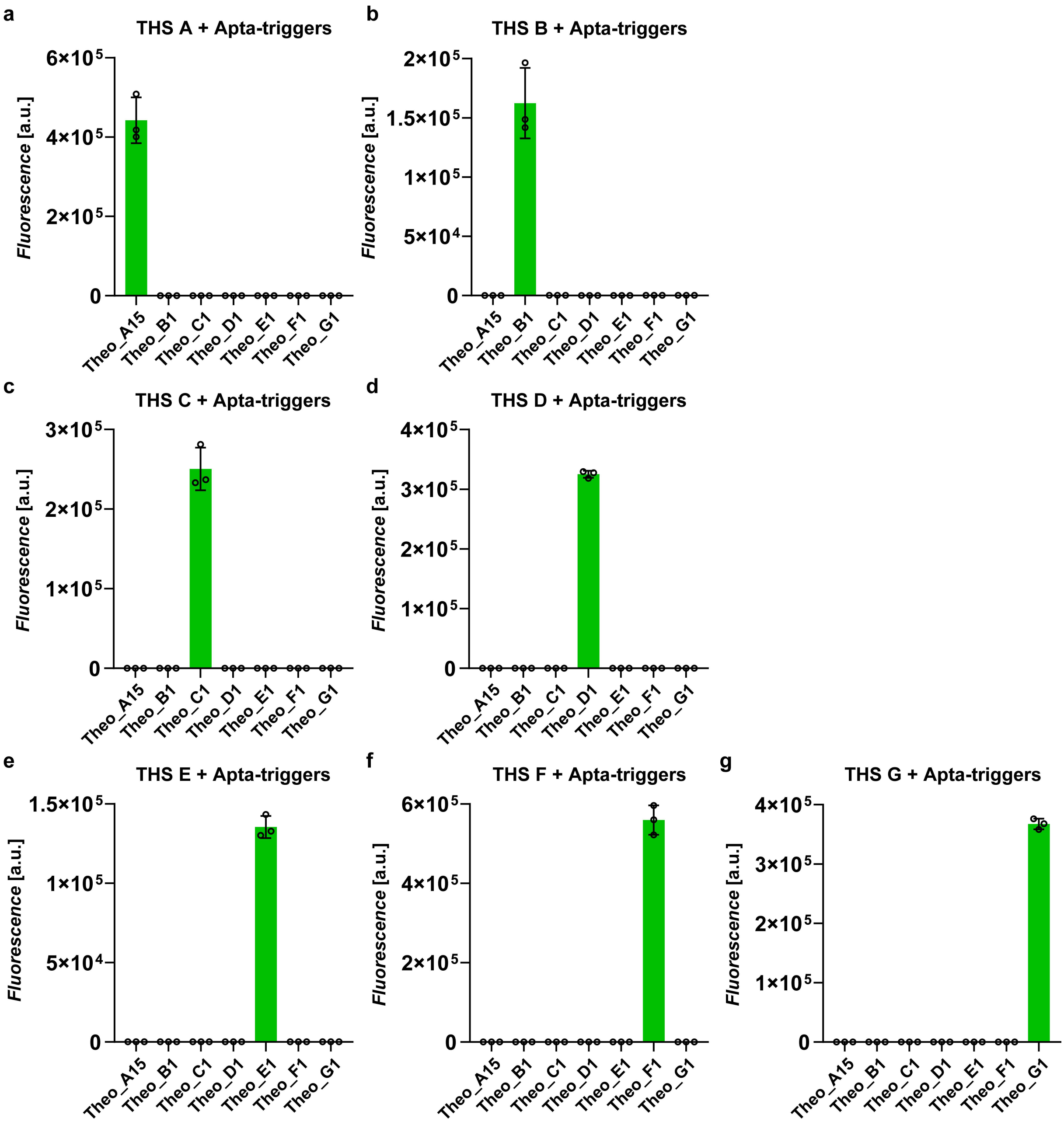

Figure S19. Orthogonality between switch RNA and partial trigger domains

a-g) GFP fluorescence of theophylline Apta-triggers with distinct partial trigger domains paired with different switch RNAs: (a) THS A, (b) THS B, (c) THS C, (d) THS D, (e) THS E, (f) THS F, and (g) THS G. Theophylline was treated at 10 mM throughout all experiments. Error bars are the SD from three biological replicates.

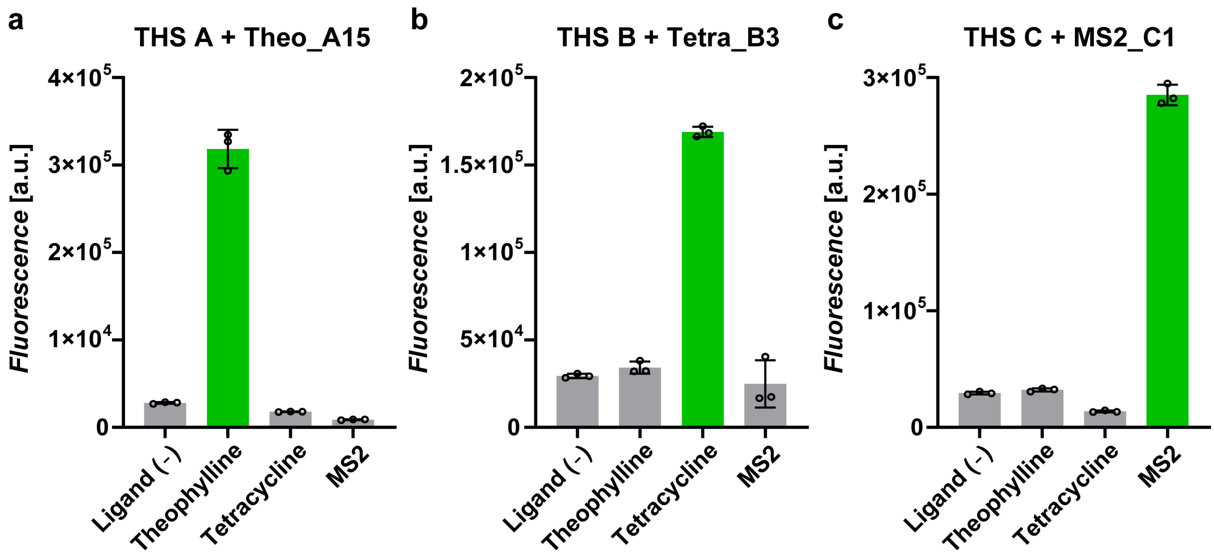

Figure S20. Orthogonality between STARTs and ligands

a-c) GFP fluorescence in response to the treatment of different input ligands for each START construct: START for (a) theophylline, (b) tetracycline, and (c) MS2 coat protein. Theophylline was treated at 10 mM, tetracycline was treated at 12 μM, and MS2 protein was induced with 10 ng mL^{-1} aTc treatment. Error bars are the SD from three biological replicates.

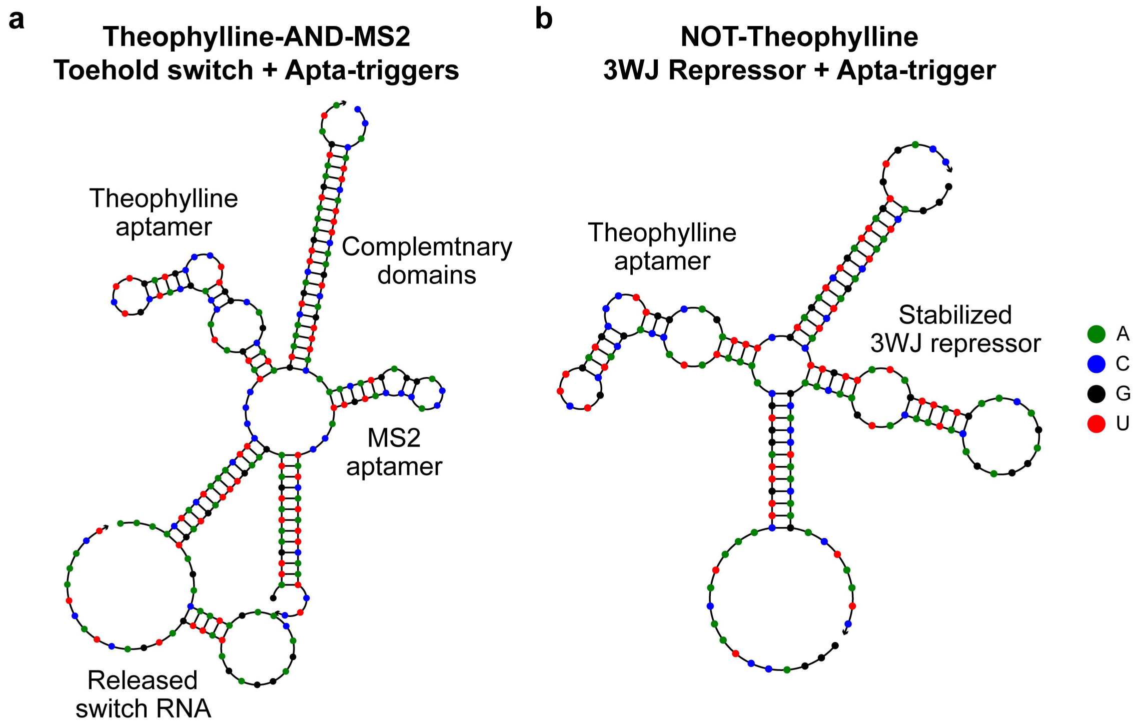

Figure S21. Predicted secondary structures of logic gate complex.

a, b) Predicted secondary structure of (a) Theophylline-AND-MS2 and (b) NOT-Theophylline logic gate complex, by NUPACK.

References

[1] J. N. Zadeh, C. D. Steenberg, J. S. Bois, B. R. Wolfe, M. B. Pierce, A. R. Khan, R. M. Dirks, N. A. Pierce, *J Comput Chem.* **2011**, *32* (1), 170.
[2] D. H. Mathews, J. Sabina, M. Zuker, D. H. Turner, *J Mol Biol.* **1999**, *288* (5), 911.
[3] Z. J. Lu, D. H. Turner, D. H. Mathews, *Nucleic Acids Res.* **2006**, *34* (17), 4912.
[4] a) T. Xia, J. SantaLucia, Jr., M. E. Burkard, R. Kierzek, S. J. Schroeder, X. Jiao, C. Cox, D. H. Turner, *Biochemistry.* **1998**, *37* (42), 14719; b) D. H. Turner, D. H. Mathews, *Nucleic Acids Res.* **2010**, *38* (Database issue), D280; c) M. J. Serra, D. H. Turner, *Methods Enzymol.* **1995**, *259*, 242; d) M. Zuker, *Nucleic Acids Res.* **2003**, *31* (13), 3406.
[5] A. A. Green, J. Kim, D. Ma, P. A. Silver, J. J. Collins, P. Yin, *Nature.* **2017**, *548* (7665), 117.
[6] a) R. Lorenz, S. H. Bernhart, C. Höner Zu Siederdissen, H. Tafer, C. Flamm, P. F. Stadler, I. L. Hofacker, *Algorithms Mol Biol.* **2011**, *6*, 26; b) J. S. Reuter, D. H. Mathews, *BMC bioinformatics.* **2010**, *11*, 1.
[7] a) Q.-L. Zhang, L.-L. Wang, Y. Liu, J. Lin, L. Xu, *Nature Communications.* **2021**, *12* (1), 4654; b) A. T. Catherine, S. N. Shishido, G. A. Robbins-Welty, A. Diegelman-Parente, *FEBS Open Bio.* **2014**, *4*, 788.
[8] J. Kim, Y. Zhou, P. D. Carlson, M. Teichmann, S. Chaudhary, F. C. Simmel, P. A. Silver, J. J. Collins, J. B. Lucks, P. Yin, A. A. Green, *Nat Chem Biol.* **2019**, *15* (12), 1173.
[9] G. R. Zimmermann, C. L. Wick, T. P. Shields, R. D. Jenison, A. Pardi, *Rna.* **2000**, *6* (5), 659.
[10] Y. Nakahira, A. Ogawa, H. Asano, T. Oyama, Y. Tozawa, *Plant Cell Physiol.* **2013**, *54* (10), 1724.
[11] D. F. Browning, R. E. Godfrey, K. L. Richards, C. Robinson, S. J. W. Busby, *Biochem Soc Trans.* **2019**, *47* (2), 755.
[12] G. Peters, B. De Paepe, L. De Wannemaeker, D. Duchi, J. Maertens, J. Lammertyn, M. De Mey, *Biotechnol Bioeng.* **2018**, *115* (7), 1855.
[13] R. Cuero, J. Lilly, D. S. McKay, *J Biotechnol.* **2012**, *158* (1-2), 1.
[14] a) A. C. Chang, S. N. Cohen, *J Bacteriol.* **1978**, *134* (3), 1141; b) V. Hershfield, H. W. Boyer, C. Yanofsky, M. A. Lovett, D. R. Helinski, *Proc Natl Acad Sci U S A.* **1974**, *71* (9), 3455; c) D. Held, K. Yaeger, R. Novy, *InNovations.* **2003**, *18* (3).
[15] J. Lloyd, C. H. Tran, K. Wadhwani, C. Cuba Samaniego, H. K. K. Subramanian, E. Franco, *ACS Synth Biol.* **2018**, *7* (1), 30.
[16] L. Qi, J. B. Lucks, C. C. Liu, V. K. Mutalik, A. P. Arkin, *Nucleic Acids Res.* **2012**, *40* (12), 5775.
